# Identifying critical age and gender-based metabolomic shifts in a Japanese population of the Tohoku Medical Megabank cohort

**DOI:** 10.1038/s41598-024-66180-0

**Published:** 2024-07-08

**Authors:** Miyuki Sakurai, Ikuko N. Motoike, Eiji Hishinuma, Yuichi Aoki, Shu Tadaka, Mana Kogure, Masatsugu Orui, Mami Ishikuro, Taku Obara, Naoki Nakaya, Kazuki Kumada, Atsushi Hozawa, Shinichi Kuriyama, Masayuki Yamamoto, Seizo Koshiba, Kengo Kinoshita

**Affiliations:** 1 Tohoku Medical Megabank Organization, Tohoku University, Sendai, Japan; 2 Graduate School of Information Sciences, Tohoku University, Sendai, Japan; 3 Advanced Research Center for Innovations in Next-Generation Medicine, Tohoku University, Sendai, Japan; 4 Graduate School of Medicine, Tohoku University, Sendai, Japan; 5 Tohoku University Hospital, Tohoku University, Sendai, Japan; 6 International Research Institute of Disaster Science, Tohoku University, Sendai, Japan

**Keywords:** Biomarkers, Metabolomics

## Abstract

Understanding the physiological changes associated with aging and the associated disease risks is essential to establish biomarkers as indicators of biological aging. This study used the NMR-measured plasma metabolome to calculate age-specific metabolite indices. In doing so, the scope of the study was deliberately simplified to capture general trends and insights into age-related changes in metabolic patterns. In addition, changes in metabolite concentrations with age were examined in detail, with the period from 55–59 to 60–64 years being a period of significant metabolic change, particularly in men, and from 45–49 to 50–54 years in females. These results illustrate the different variations in metabolite concentrations by sex and provide new insights into the relationship between age and metabolic diseases.

## Introduction

Aging is a universal phenomenon among all living organisms, changing individual biological and physical characteristics in humans. These manifest as a decrease in function at cellular to systemic levels and overall physiological changes. Additionally, aging increases the risk of disease onset, affecting overall health^1–4^. Therefore, from the perspective of preventing diseases whose incidence increases with age, there is a need for biomarkers that give information about the likelihood of being biologically old and criteria to determine whether the body is experiencing functional decline compared to others of the same age, i.e., a biological age index.

Blood is a good reflection of the state of health at the time of collection, of which metabolomic analysis is the subject of extensive basic, clinical, and epidemiological research into the mechanisms of the relationship between metabolites and disease^5–12^. Metabolic disorders often lie dormant for years before clinical symptoms appear, indicating a strong correlation between aging and metabolic diseases, such as diabetes and hypertension^13–18^. As a result, conditions such as cardiovascular diseases (CVDs) and complex disorders of the heart and vessels induced by atherosclerosis are often diagnosed. Furthermore, it is suggested that disturbances and changes in lipid and glucose metabolism, which typically begin in females in their mid-forties during menopause and the pre-menopausal transition, have significant health effects, with a close association also suggested with CVDs. This association is thought to occur not because hormone levels independently predict dyslipidemia or CVD onset but because the hormonal changes of menopause impact body fat increase and CVD risk through alterations in lipid profiles^19,20^.

Many studies have shown that their age and sex influence the way individuals metabolize lipids and amino acids^21–26^. Moreover, genetic and molecular differences also impact how people respond to medication and its side effects. To achieve personalized medicine, it’s essential to understand the unique characteristics of specific groups^27–29^. Thus, one of the goals of the Tohoku Medical Megabank (TMM) Cohort Project is to develop age-specific indicators of omics data. This project is Japan’s first and largest population-based prospective cohort study of healthy volunteers^30–32^.

The omics analysis of metabolites can be influenced by factors such as age, sex, body shape (Body Mass Index (BMI)), disease, diet, genetic polymorphism, and the time and conditions of specimen collection^9,33–38^. Therefore, it’s necessary to collect detailed information on these factors to evaluate their influence on omics data and consider them appropriately during analysis. This research aimed to simplify these factors, target many participants, derive more general trends and conclusions, and gain insights from the perspective of aging-related health risks and disease prevention in the entire population or specific groups. The obtained results were compiled and summarized as the “jMorp: Japanese Multi-Omics Reference Panel” on the web, which serves as a source of reference data for the scientific community as open data for the Japanese population^39^.

## Results and discussion

### Stratifications of metabolite concentrations by age and sex

**Figure 1:**
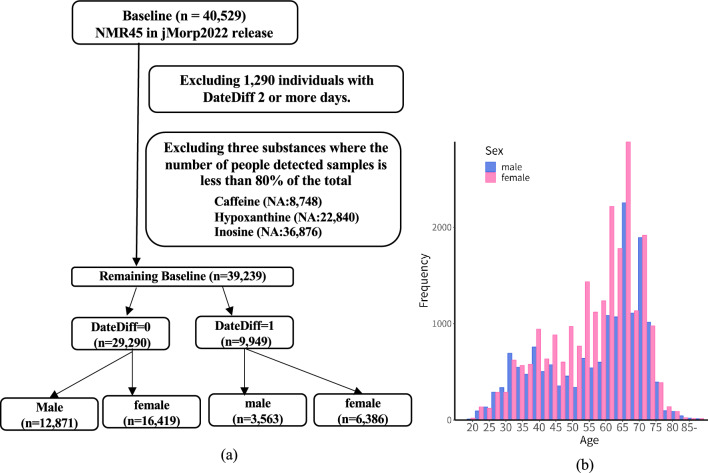
Design for the generation of age-specific metabolite concentration indices using NMR-derived plasma metabolomics data; (**a**) shows the stratification flow and (**b**) shows the age distribution of study participants.

We examined the two significant factors affecting metabolite concentration: the number of days from blood collection to processing to storage (Same Day: DateDiff0 or Next Day: DateDiff1) and sex (Male or female), as in the flow shown in Fig. 1a. If significant differences were found between these, we would divide the groups by each factor for the analysis. Our test results are summarized in Supplementary Table S1 and described in detail below.

First, we checked for any systematic differences between DateDiff0 and DateDiff1. Since the results of the K-S test showed that metabolites lacked normality, a Wilcoxon rank sum test was performed to check for differences in metabolite concentrations between DateDiff0 and DateDiff1. As shown in Supplementary Table S1, all metabolite concentrations significantly differ between DateDiff0 and DateDiff1 samples (*p* < 0.0001); therefore, separate statistics are used in the following analysis.

Next, we checked the difference in the coefficient of variation, and there were 17 types where DateDiff0 < DateDiff1, two types where DateDiff0 = DateDiff1, and 23 types where DateDiff0 > DateDiff1, indicating that there were many metabolites where the coefficient of variation of DateDiff0 was larger than that of DateDiff1. Specifically, among the metabolites with a difference of 0.01 or more, the metabolites with a larger coefficient of variation in DateDiff0 than in DateDiff1 were Acetone, Succinate, Threonine, Alanine, Leucine, 3-Hydroxybutyrate, Lactate, Glycerol, and Isoleucine. There could be several reasons for these differences. First, when the number of participants is larger, the likelihood of outliers increases, which could have been a factor in increasing the variability of the data. The presence of different subgroups can also increase variability. Sex differences (male–female differences) are one example of this, and in this study, significant differences were found in all metabolites in both DateDiff0 and DateDiff1 because of statistical testing. In Rist et al.^25^, BCAA (branched-chain amino acids) and their metabolites have been shown to have higher concentrations in males than females, contributing to sex differences. In this study, BCAA (Valine, Leucine, Isoleucine) and Glutamine, a metabolite of Isoleucine, showed higher concentrations in all age groups in males than in females in DateDiff0. This suggests that the variability within DateDiff0 may be influenced by the presence of different subgroups due to sex differences. Similar results regarding sex differences have been reported in many previous studies^11,21–24^, and it is widely known that there are differences between males and females in metabolite concentrations.

From these results, the analysis was conducted separately for DateDiff0 and DateDiff1 data and divided by sex. To confirm the differences due to sex, we again used the Wilcoxon rank-sum test to the separated date set. As expected, significant differences were found in all metabolites (*p* < 0.0001).

### Change of the concentration of each metabolite against age

For the divided data sets, we observed trend changes for each age group, and a metabolite index was calculated as the mean and the standard deviation of metabolite concentration (Supplementary Table S22). From the viewpoint of the diversity and reliability of the concentrations, the standard deviation for each metabolite is focused, and the trend by age is considered. The smaller the standard deviation, the higher the reliability of the data in the age group, and it is deemed to have stability in interpretation and prediction.

**Figure 2:**
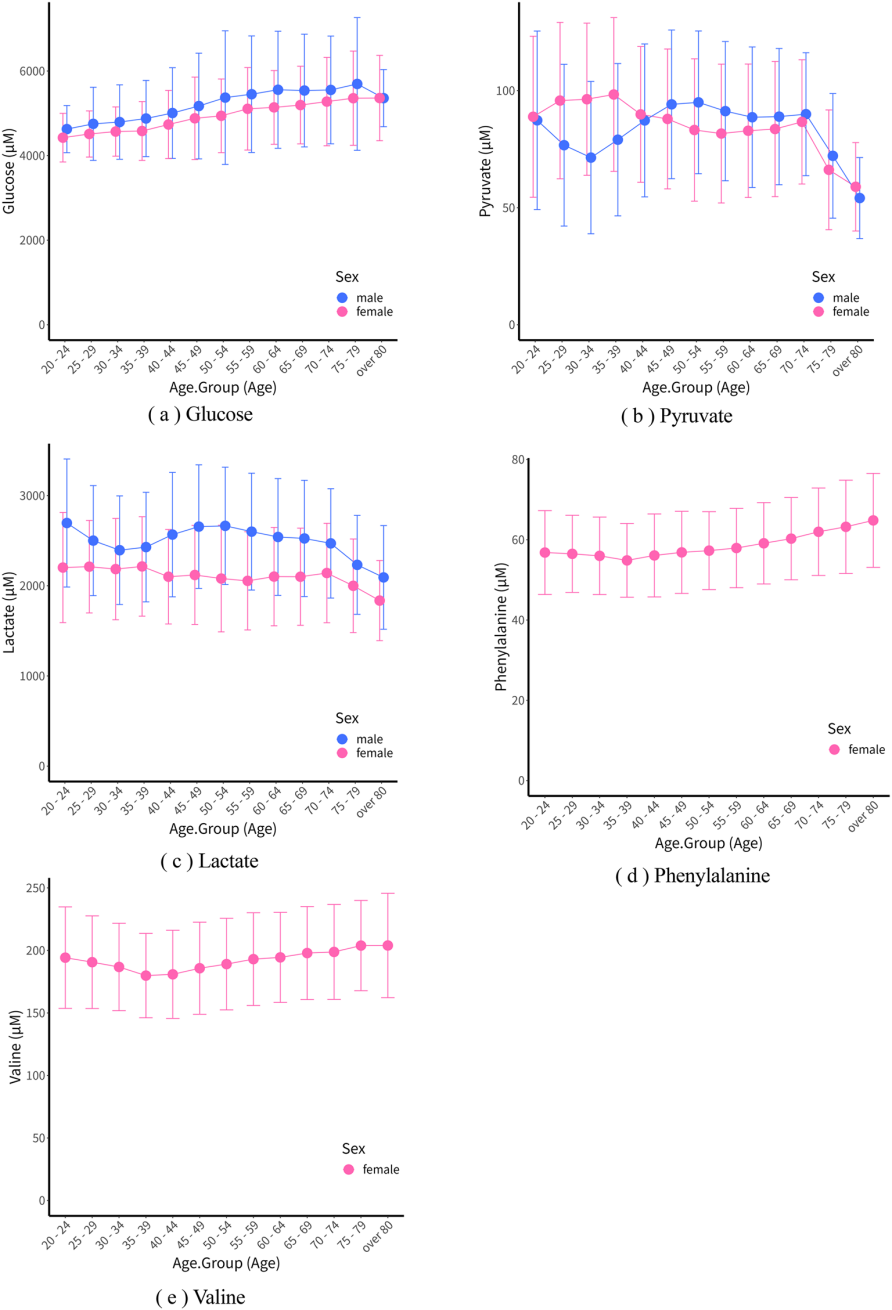
Blue for males and pink for females, From left to right: glucose (**a**) and pyruvate (**b**), respectively; bottom left: lactate (**c**); (**d**) shows mean phenylalanine values for females by age group; (**e**) shows mean valine values for females by age group (error bars are standard deviations).

First, the metabolome indexes were compared in the male age groups from 20 to over 80. As a result, the metabolites Acetate, Glucose, Glutamine, Tyrosine, and Lysine showed the most minor standard deviations (SD) in the 20–24 age group for both DateDiff0 and DateDiff1. Notably, Glucose (Fig. 2a) was found to have the lowest mean value across all age groups. This suggests a consistent trend of lower values for these metabolites in the early 20 s. Also, the age group with the most significant SD for Glucose was 50–54 for DateDiff0 and over 80 for DateDiff1. On the other hand, the highest mean value was seen in the 75–79 age group for both DateDiff0 and DateDiff1. Similarly, Glycerol shows the most minor standard deviation and mean values in the 25–29 age group for both DateDiff0 and DateDiff1.

In the typical middle age of humans, from 40 to 60 years old, very few metabolites showed the most minor standard deviation in DateDiff0. Creatine showed the slightest standard deviation for ages 45–49 in both DateDiff0 and DateDiff1. For ages 55–59, 3-Hydroxyisobutyrate only showed the slightest deviation in DateDiff0, while many metabolites exhibited the highest standard deviation. For example, in the 50–54 age group, Acetate, Glucose, 2-hydroxybutyrate, Cysteine, Alanine, Methionine, Glutamate, Leucine, and Phenylalanine showed the highest standard deviation. This age group tended to have the highest standard deviation in many metabolites, second only to the group over 80.

In the age group 75–79 for males, the metabolites with the most minor standard deviation in both DateDiff0 and DateDiff1 were Lactate, 2-Oxoisocaproate, and Proline. Among these, the average values of 2-Oxoisocaproate and Proline in DateDiff0 were the smallest among the age groups, while Lactate had the smallest average value in DateDiff1. Although the number of males in the age group over 80 years old was small (63 for DateDiff0, 105 for DateDiff1), Glutamate, Threonine, N, N-Dimethylglycine, and Pyruvate showed the most minor standard deviation in both DateDiff0 and DateDiff1. Glutamate had the smallest average value in both DateDiff0 and DateDiff1.

The study by Ko et al.^40^ investigated the relationship between age and blood glucose levels. The results indicated that blood glucose levels gradually increase with age, as age increases. Generally, younger people tend to have higher metabolic activity, with more active synthesis and breakdown of substances. As a result, metabolites such as Glucose may be consumed more quickly, leading to lower concentrations. On the one hand, it has been reported that glucose tolerance is reduced and glucose metabolism is decreased in the elderly^13,41,42^. According to Basu et al.^14^, the mechanism of age-related impaired glucose tolerance is that older people generally tend to have reduced muscle mass and increased body fat mass, and these body composition. These changes in body composition tend to improve insulin resistance and decrease insulin secretion with aging. This age-related decline in glucose tolerance is also a concern for the increased incidence of metabolic diseases such as diabetes^42,43^. For these reasons, indicators of metabolites associated with the glycolytic system, such as glucose and pyruvate, are essential in old age (Fig. 2a-c).

Next, the trends of metabolites according to age in females were evaluated. The SD of Proline and Glycine was the smallest in the 20–24 age group in both DateDiff0 and DateDiff1. Furthermore, regarding average values, Proline had the most significant value in the age group, while Glycine had the smallest. For Glycine, the age group with the highest standard deviation and average values was 55–59 in both DateDiff0 and DateDiff1. For Glucose, which showed the most minor standard deviation in males in the 20–24 age group, females showed the most minor standard deviation and average values in DateDiff1 only (in DateDiff0, the most minor standard deviation was in the 25–29 age group). For Acetate, the most minor standard deviation and average value were shown in DateDiff0 for females.

In females aged 35–39, 2-aminobutyrate, Isoleucine, Phenylalanine, and Valine showed the most minor standard deviation in both DateDiff0 and DateDiff1. Among these, Phenylalanine and Valine also had the smallest average values in both DateDiff0 and DateDiff1 (Fig. 2d-e). The reasons for the decrease in the metabolite concentrations of phenylalanine and valine in females during this age period could be changes in nutrient intake. Phenylalanine and Valine are essential amino acids in foods such as soy, dairy products, fish, meat, and eggs. Changes in dietary habits, appetite, nutrient intake, and meal content could decrease the intake of these amino acids. Moreover, Phenylalanine and Valine are components of proteins and are involved in protein synthesis and energy production in the body. Therefore, decreasing metabolite concentrations may suggest protein and energy metabolism changes (Supplementary Fig. S1-1,2).

Also, in the age group 70–74, 3-Hydroxybutyrate showed the smallest standard deviation in both DateDiff0 and DateDiff1. In the age group 75–79, the standard deviation of 2-Oxoisocaproate was the smallest. In the group over 80 years old, the standard deviation of Pyruvate and Carnitine was smallest in both DateDiff0 and DateDiff1. Compared to males, the same results were seen in females, with the smallest standard deviation of 2-Oxoisocaproate in the 75–79 age group and Pyruvate in the group over 80.

In this way, trends by age became clear for each metabolite. The trend of small standard deviations in metabolite concentration represents the degree of individual differences or outliers among the participants in that age range, and generally, the smaller the standard deviation, the less variation in metabolite concentrations, indicating a more stable state. This suggests metabolite concentrations fluctuate within a specific range, showing relatively consistent values. When comparing metabolite concentrations by age group, the age groups with smaller standard deviations showed common trends in both DateDiff0 and DateDiff1. In this way, similar results and trends were obtained in the datasets of DateDiff0 and DateDiff1, which have separate distributions, which can be said to support the validity of the data. This is because consistent results were obtained regarding the values of metabolite concentrations despite the different datasets. This suggests the reliability and consistency of the data, demonstrating the robustness of our research results. This has clarified the similarities and expected trends in metabolite concentrations between the datasets of DateDiff0 and DateDiff1. This information will enhance the reproducibility and generalizability of our research results. From these results, it was shown that the datasets used in our research have high validity. This indicates that our research results are reliable and can be expected to provide helpful information for other research and clinical applications.

### The transition of concentrations among age

In our analysis of age-related alterations in metabolite concentration, we segmented our study population into specific age groups. We conducted a comparative analysis using the Wilcoxon Rank Sum Test (with Bonferroni-adjusted *p*-value < 0.05).

To underscore the significance of these age-driven metabolic shifts, we limited our analysis to comparisons between consecutive age groups (e.g., between the [20–24 years group and the 25–29 years group]). Instances of significant differences are interpreted as drastic transitions in metabolite concentration attributable to aging. This approach allows us to pinpoint precise age intervals where substantial metabolic alterations occur, thereby spotlighting the pervasive impact of aging on metabolite levels. Since the results of DateDiff0 and DateDiff1 are quite small, we will focus on the description for DateDiff0 hereafter.

As a result of the Wilcoxon rank sum test, there were 22 metabolites where significant differences were seen in any age group comparisons for males in DateDiff0. Supplementary Table S3-1 summarizes the results of the age group comparison in DateDiff0 for males. Wilcoxon test p-values for age groups where significant differences were seen are shown in red, and effect sizes are displayed in green. Similarly, Supplementary Table S3-2 summarizes the results for females in DateDiff0. In DateDiff0 for females, there were 37 metabolites where significant differences were seen in any age group comparisons.

**Figure 3:**
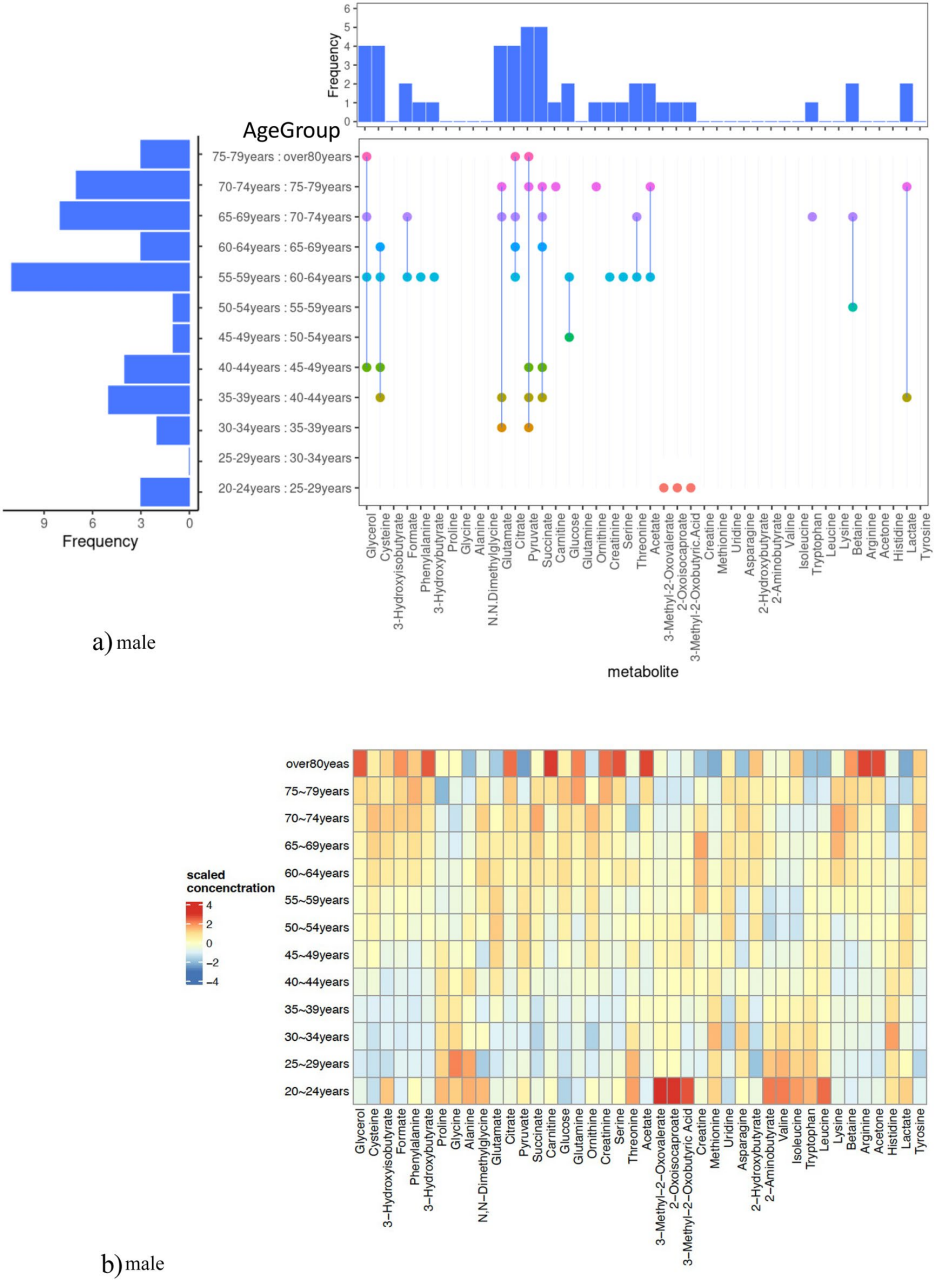

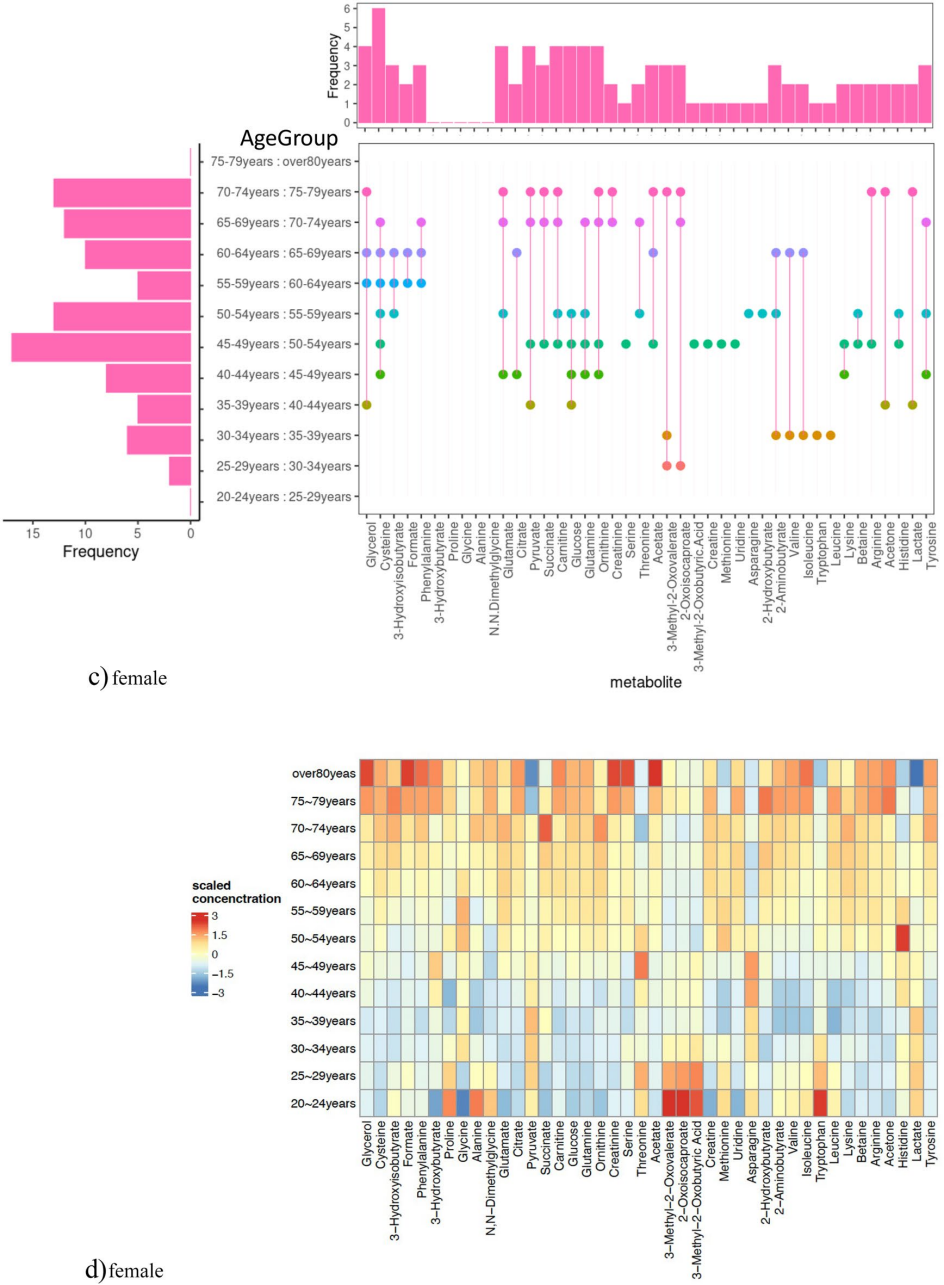
(**a**) shows the presence or absence of significant differences among age groups for each metabolite in males, while (**c**) does the same for females. The left side of the figure displays the number of metabolites with significant differences among the compared age groups, and the right side shows the frequency of significant differences observed for each metabolite. The top part of the figure indicates the frequency of significant differences, and the bottom part includes markers denoting significant differences among the compared age groups. (**b**) presents a heatmap of the metabolite concentrations across different age groups in males, and (**d**) displays a similar heatmap for females.

Figure 3a(left side) and c(left side) detail the significant metabolite differences across age groups. For males, the most drastic metabolic changes were observed between ages 55–59 and 60–64 with 11 important metabolites, followed by ages 65–69 and 70–74 with eight, and 70–74 and 75–79 with seven. Meanwhile, females showed the most substantial changes between ages 45–49 and 50–54, with 17 significant metabolites, and between 50–54 and 55–59, with 13. Notably, the male-dominant age range of 55–59 to 60–64 showed five significant metabolites (Glycerol, Cysteine, 3-Hydroxyisobutyrate, Formate, and Phenylalanine) in females, four of which excluding 3-Hydroxyisobutyrate, were also effective in males. Older female age groups also exhibited many significant changes, with 10, 12, and 13 important metabolites in the age range 60–64 to 65–69, 65–69 to 70–74, and 70–74 to 75–79, respectively. They are listed in Tables 1 and 2. Interestingly, while 20 metabolites showed no age group differences in males, only five metabolites (3-Hydroxybutyrate, Proline, Glycine, Alanine, N.N-Dimethylglycine) in females displayed similar traits. Four metabolites other than 3-Hydroxybutyrate were common to both sexes, indicating they might be less influenced by aging. For proline and glycine, the MGWAS analysis of Koshiba et al.^9^ showed significant associations between genetic factors and these metabolites. Therefore, the effects of this genetic regulation may have a moderating effect on age-related changes. Figure 3a(right side) and c(right side) show the frequency of significant differences for each metabolite at the top and between age groups where significant differences were found at the bottom. Males had ten and females nine metabolites, with significant differences in only one age group. Glutamate, Glycerol, Citrate, Cysteine, Pyruvate, and Succinate in males, and Glutamate, Glycerol, Carnitine, Glucose, Glutamine, Ornithine, Pyruvate, and Cysteine in females demonstrated significant differences in four or more times, suggesting these metabolites are likely age sensitive.

**Table 1:** A list of metabolites with significant concentration changes observed across different age categories and their respective frequencies.

Frequency	Male	Female
1	3-Methyl-2-Oxobutyric Acid Carnitine Serine Tryptophan 2-Oxoisocaproate 3-Hydroxybutyrate 3-Methyl-2-Oxovalerate Creatinine Ornithine Phenylalanine	3-Methyl-2-Oxobutyric Acid Creatine Serine Tryptophan 2-Hydroxybutyrate Asparagine Leucine Methionine Uridine
2	Acetate Betaine Formate Lactate Threonine Glucose	Acetone Betaine Formate Lactate Threonine Arginine Citrate Creatinine Histidine Isoleucine Lysine Valine
3	–	2-Aminobutyrate 2-Oxoisocaproate 3-Hydroxyisobutyrate 3-Methyl-2-Oxovalerate Acetate Phenylalanine Succinate Tyrosine
4	Glutamate Glycerol Citrate Cysteine	Glutamate Glycerol Carnitine Glucose Glutamine Ornithine Pyruvate
5	Pyruvate Succinate	–
6	–	Cysteine

**Table 2:** A few examples of metabolites with concentrations change across different age categories.

		Male				Female		
Comparison age groups	No	Name	Low Age Group Mean	High Age Group Mean	No	Name	Low Age Group Mean	High Age Group Mean
45–49: 50–54 years	**1**				**17**	Acetate	32.74	35.11
						Arginine	45.60	48.86
						Betaine	41.09	44.29
						Carnitine	34.69	35.99
						Creatine	37.81	39.95
						Cysteine	51.09	53.23
		**Glucose**	5171.74	5371.06		**Glucose**	4881.40	4940.30
						Glutamine	459.38	475.07
						Histidine	83.22	84.86
						Lysine	122.72	127.79
						Methionine	25.54	26.23
						Ornithine	63.78	66.50
						Pyruvate	87.92	83.20
						Serine	111.68	114.24
						Succinate	7.49	7.73
						Uridine	4.00	4.15
						3.Methyl.2.Oxobutyric.Acid	8.73	8.41
55–59: 60–64 years	**11**	Acetate	36.21	36.26	**5**			
		Citrate	93.22	98.67				
		Creatinine	66.83	68.84				
		**Cysteine**	62.61	65.27		**Cysteine**	55.95	58.70
		**Formate**	11.25	11.73		**Formate**	10.94	11.29
		Glucose	5451.28	5556.63				
		**Glycerol**	61.33	64.64		**Glycerol**	73.16	77.46
		**Phenylalanine**	62.98	64.89		**Phenylalanine**	57.93	59.12
		Serine	111.96	114.32				
		Threonine	184.50	188.88				
		3.Hydroxybutyrate	70.22	79.65		3.Hydroxyisobutyrate	10.57	10.90
70–74: 75–79 years	**7**	**Acetate**	37.24	40.35	**13**	**Acetate**	37.01	40.92
						Acetone	6.58	8.64
						Arginine	49.94	52.85
		**Carnitine**	39.20	40.89		**Carnitine**	36.92	39.36
						Creatinine	54.83	58.58
		**Glutamate**	58.04	53.91		**Glutamate**	49.62	45.93
						Glycerol	83.10	95.12
		**Lactate**	2469.85	2231.89		**Lactate**	2141.32	1999.75
		**Ornithine**	75.93	72.80		**Ornithine**	70.98	66.85
		**Pyruvate**	89.95	72.15		**Pyruvate**	86.66	66.19
		**Succinate**	8.49	8.10		**Succinate**	8.34	7.74
						2.Oxoisocaproate	24.00	25.19
						3.Methyl.2.Oxovalerate	18.32	19.17

Common metabolites in both males and females are shown in boldface.

Focusing on the male age range [55–59 years: 60–64 years], 22 metabolites showed significant differences in some age groups, and half showed differences in this age group. Five metabolites (Glucose, Creatinine, Phenylalanine, 3-hydroxybutyrate, and Serine) showed no significant changes after this range. Six metabolites (Threonine, Glycerol, Citrate, Formate, Cysteine, and Acetate) showed significant differences in some older age groups. The remaining 11 metabolites demonstrated differences in older age ranges after this group, except 3-methyl-2-oxovalerate, 2-oxoisocaproate, and 3-methyl-2-oxobutyricacid.

In females, the age range [45–49 years: 50–54 years] was notable for metabolic shifts. Of the 37 metabolites with differences across age groups, 17 showed significant differences in this range. Five metabolites (Creatine, Methionine, Serine, Uridine, and 3-methyl-2-oxobutyricacid) showed changes unique to this range. Other metabolites began to change significantly after this range or showed long-term changes before and after. A few metabolites (Asparagine, 2-hydroxybutyrate, 3-hydroxyisobutyrate, and 2-aminobutyrate) showed significant differences only in specific ranges. In contrast, others (Phenylalanine, Glycerol, Formate, Valine, Isoleucine, Tryptophan, Leucine, and 3-methyl-2-oxovalerate) exhibited unique trends in specific age ranges. The period from the late 40 s to early 50 s, where the most significant differences in metabolite concentrations were seen, typically corresponds to the perimenopause period in females. Many preceding studies have established the influence of hormonal changes on metabolites during this period^18,20,36^. In Watanabe et al.^18^, a study was conducted comparing plasma metabolite concentrations between premenopausal and postmenopausal females aged 40–60. The trends observed in this age group (45–49 and 50–54 years) in our study were found to be like those in Watanabe et al.^18^. The metabolites were Ornithine, Glutamine, Lysine, Carnitine, Betaine, and Arginine. All these metabolites showed higher average values in the postmenopausal group in Watanabe et al.^18^; in our study, the age group of 50–54 showed significantly higher average values than the age group of 45–49. Furthermore, while Ornithine, Glutamine, and Lysine showed significant differences between premenopausal and MT (transition period) groups, no significant difference was observed with Carnitine, Betaine, and Arginine in the MT group, which lies between premenopause and postmenopause. In our study, Ornithine, Glutamine, and Lysine showed significant differences when comparing the age groups 40–44 and 45–49, and Betaine and Carnitine showed significant differences when comparing the age groups 50–54 and 55–59. These results suggest that our study has detailed the influence of age-related and longitudinal changes on these metabolites. From these results, similar trends were confirmed with the study by Tsuruoka (Watanabe et al.^18^), which supports the objectivity and reliability of our study and demonstrates that our study results can capture age-related changes. Comparing males and females in Tables 2, we could observe more age groups where changes occur in females. One reason for the increase in the number of metabolites that change specifically in females between the late 40 s and early 50 s could be the influence of hormones, as similar results have been obtained by Watanabe et al.^18^. Additionally, in the older age groups (70–74 years and 75–79 years), the number of metabolites with similar types and trends of change (whether average values increase or decrease with age) has increased, suggesting a potential relationship with metabolic diseases such as diabetes and hypertension (13–18).

### Impact of BMI

We analyzed how age and BMI affect metabolite levels using multiple regression. Age was independent, and BMI was an adjustment variable. Where interaction effects between age and BMI were found, they were interpreted as interacting with each other, and a Simple Slope Analysis was performed.

Supplementary Table S4-1 shows that in males, 17 metabolites were affected by age and BMI interactions. Among them, 11 metabolites, namely 3-Hydroxyisobutyrate, Cysteine, Creatinine, Tyrosine, Glycerol, 3-Hydroxybutyrate, Serine, Betaine, 2-Oxoisocaproate, Tryptophan, and 3-Methyl-2-Oxobutyricacid, showed significant differences across all BMI ranges. Three metabolites, including 2-oxoisocaproate, Tryptophan, and 3-methyl-2-oxobutyricacid, decreased with age. The four metabolites 2-Hydroxybutyrate, Threonine, Proline, and Glycine differed only in the low BMI range, while Threonine, Proline, and Glycine decreased with age. Finally, Asparagine and Lactate only differed in the higher BMI range, with Lactate decreasing with age.

As in Supplementary Table S4-2 in females, nine metabolites showed significant effects and interactions between age and BMI. Seven metabolites (2-aminobutyrate, Cysteine, Glutamate, Leucine, Serine, Valine, and Citrate) differed across all BMI ranges and increased with age. Asparagine, only differing in the lower BMI range, and Lactate, only differing in the higher BMI range, decreased with age (Supplementary Fig. S2-1,2).

In terms of the effect of BMI, we evaluated the trend of the variable under conditions where the interaction effect with age was confirmed by performing a simple slope analysis (Supplementary Fig. S2-1,2). In Moore et al.^35^, a linear regression analysis was conducted after adjusting for age, sex, and smoking status, and 37 types of metabolites were reported to be related to BMI. Of the metabolites that were related to BMI in that study, those common to our study were eight types in males (3-hydroxyisobutyrate, 3-methyl-2-oxobutyrate, Glycerol, Tyrosine, Asparagine, lactate, 2-hydroxybutyrate, Glycine) and three types in females (Glutamate, Leucine, Valine). Our study also compared the partial regression coefficients (effect sizes) of BMI in the high BMI group for these metabolites in Supplementary Table S4-1,2 (only metabolites with significant main effects and interactions are listed). The contributions of these metabolites to the model (signs of β) all matched. Also, in Ottosson et al.^37^, a linear regression analysis was performed after adjusting for age and sex, and 19 types of metabolites were reported to have a significant association with BMI. Of these, the metabolites common to our study were four types in males (Tyrosine, Serine, Proline, Asparagine) and six types in females (Glutamate, Leucine, Serine, Valine, Citrate, Asparagine). All these metabolites matched the signs of the partial regression coefficients in the high BMI group in our study. From these, it is suggested that BMI consistently influences the fluctuations of these metabolites, indicating that our study has obtained highly reliable results. Our study clarified how the interaction of BMI and age regulates the concentrations of metabolites. For example, Tyrosine (in males) and Glutamate (in females), which showed a relationship with BMI in all the studies compared, and Lactate, which showed similar results in males and females in our study, are further explained.

In males, the Tyrosine concentration was higher in the high BMI group than in the low BMI group (mean + 1SD, β = 0.176, *p* < 0.001) and showed an increasing trend with age. Similarly, in females, Glutamate concentration was higher in the high BMI group compared to the low BMI group (mean + 1SD, β = 0.124, *p* < 0.001) and increased with age. As for Lactate, significant differences were observed between the age groups of 35–39 and 40–44 in both males and females, with an increase observed in males and a decrease in females. Subsequently, females reached a concentration peak in the age range of 35–39, while males had the highest peak between the ages of 50 and 54. Significant differences were observed between the age groups of 70–74 and 75–79 in both males and females, where Lactate concentration significantly decreased. Moreover, in both males and females, higher BMI values combined with increasing age showed a tendency for Lactate concentration to decrease (Males: mean + 1SD, β = − 4.253, *p* < 0.001; Females: mean + 1SD, β = − 3.629, *p* < 0.001).

By elucidating the detailed impact of BMI and its interaction with age on metabolites, we can gain insights into the biological processes and disease mechanisms associated with these metabolites. For instance, the variations in Tyrosine and Glutamate concentrations influenced by the interaction of BMI and age suggest their potential relevance to weight management and age-related diseases. Furthermore, when metabolite changes manifest more prominently under specific combinations of BMI and age, it indicates a higher likelihood of specific health risks for individuals. Such information can contribute to individual risk prediction and aid in developing tailored treatment and management strategies, fostering the advancement of personalized medicine. We have summarized the metabolites exhibiting the main effects and interaction effects of BMI and age in Tables 3 and 4, categorized by the sign of their regression coefficients. The metabolic concentration indices we have established can accommodate various conditions and variables in research and can be generalized to other studies and population groups, ensuring flexibility and generality.

**Table 3:** A summary of multivariate analyses of each metabolite with age and BMI in males.

Male	Main effect only in age	Main effect only in BMI	Main effects both in age and BMI	Main and interaction effects in age and BMI
Positive correlations both with age and BMI	Formate	2.Aminobutyrate Alanine Isoleucine	Arginine Creatine Glucose Lysine Ornithine Phenylalanine	2.Hydroxybutyrate 3.Hydroxyisobutyrate Creatinine Cysteine Glycerol Tyrosine
Positive correlations only in age	Acetone Glutamine Succinate		Acetate Citrate	Asparagine 3.Hydroxybutyrate Serine Betaine
Positive correlations only in BMI		Valine	3.Methyl .2.Oxovalerate Methionine	2.Oxoisocaproate Tryptophan Proline Lactate 3.Methyl.2.Oxobutyric.Acid
Negative correlations both with age and BMI				Threonine Glycine

**Table 4:** A summary of multivariate analyses of each metabolite with age and BMI in females.

Female	Only main effect of age	Only main effect of BMI	Main effects of age and BMI	Main effects and interaction of age and BMI
Positive correlations both with age and BMI	Succinate Arginine	Proline	3.Hydroxyisobutyrate Methionine Creatine 2.Hydroxybutyrate Carnitine Isoleucine Phenylalanine Ornithine Tyrosine Glycerol Lysine Alanine Glutamine Glucose N.N.Dimethylglycine	2.Aminobutyrate Cysteine Glutamate Leucine Valine
Positive correlations only in age			Acetone Acetate 3.Hydroxybutyrate Betaine	Serine Citrate
Positive correlations only in BMI			3.Methyl.2.Oxovalerate 2.Oxoisocaproate Tryptophan Pyruvate Histidine 3.Methyl.2.Oxobutyric.Acid	Lactate
Negative correlations both with age and BMI				Asparagine

### Impact of meals

We have included an analysis of postprandial time in our study. Due to our cohort study’s recruitment methods, the data collection protocols, and the settings varying, the distinction in postprandial time was either continuously recorded or categorized as binary (fasted or not). Among our analyzed data, participants with recorded blood collection times included 1549 men out of 11,393 (AM: 9:23–11:59 n = 770, PM: 12:00–16:35 n = 779) and 2510 women out of 14,663 (AM: 8:34–11:59 n = 1,178, PM: 12:00–17:30 n = 1,332). Those with continuously recorded postprandial times were classified into two groups: ‘anytime’ (less than 10 h) and ‘fasted’ (10 h or more). A multiple regression analysis was performed with each metabolite concentration as the dependent variable. The independent variables are age and two groups based on the ‘Empty stomach group’ postprandial time (Empty stomach and any time), incorporating interaction terms for the two factors of age and postprandial time.

As a result, there were seven types of metabolites in males where significant main effects and interactions were observed between age groups and post-mealtime (Supplementary Table S5-1); Formate, Pyruvate, 2-oxoisocaproate, 3-methyl-2-oxovalerate, 3-methyl-2-oxobutyricacid, Citrate, and Methionine. In females (Supplementary Table S5-2), 15 types of metabolites showed significant main effects and interactions between age groups and post-mealtime; Acetate, Betaine, Formate, Glutamine, Leucine, Ornithine, Phenylalanine, Succinate, Tyrosine, Valine, 2-Aminobutyrate, 3-hydroxybutyrate, 3-methyl-2-oxovalerate, 3-methyl-2-oxobutyricacid, and Creatine.

Among these are formate, 3-methyl-2-oxovalerate, and 3-methyl-2-oxobutyric.Acid were the three metabolites that showed significant differences in both males and females.

## Conclusions

In conclusion, our study offers an in-depth analysis of age-specific metabolite concentration indices using plasma metabolomics data obtained via NMR. We deliberately simplified the scope of our research to capture generalized trends and insights into the changes in metabolic patterns associated with aging.

A significant feature of our data set is the high density of age strata within the 60 to 75 years range. This offers a unique opportunity to investigate the fluctuations of metabolites and metabolic pathway information relevant to aging. Segmenting age groups into 5-year intervals allows us to understand concentration changes and characteristics more effectively.

It’s essential to note the limitations of our study. Our initial research primarily focused on polar metabolites, which were analyzed using NMR techniques. This methodological approach aimed at capturing the general trends in age-related metabolic changes during early stages of our study. As a result, this approach excluded lipids that play a crucial role in metabolic health, especially with increasing age, where there is a higher dependency on them for energy. This exclusion may limit a comprehensive understanding of the metabolic profile. The regulation of metabolism based on the body’s internal clock is a key factor in determining levels of metabolites in the blood. The timing of blood sampling can significantly influence the metabolic profile, particularly because of the body’s internal rhythms. In our data collection process, we only had precise blood collection times for some participants. Our analysis showed that there is a connection between the time after eating and age in relation to the metabolic profile. However, we recognize that our analysis of post-mealtime does not fully consider the influence of the body’s internal rhythms. This limitation impacts how we interpret our results, as we were unable to conduct detailed analyses based on the body’s internal rhythms. The results are not based on longitudinal evaluations of the same individuals, indicating a need for further examination in future studies. For a more precise understanding of changes in human metabolites, it is necessary to track longitudinal variations through repeated measurements. Future studies could focus on a more comprehensive analysis using longitudinal data, enhancing the understanding of individual fluctuations and long-term trends. This approach will be a key focus for future research, contributing to the advancement of personalized medicine and disease prediction.

## Materials and methods

### Subjects for analysis

The subjects for our analysis were chosen from the Baseline survey participants (referred to hereafter as “Baseline”) of Tohoku Medical Megabank Cohorts, and we used 40,529 individuals (male: n = 16,923, female: n = 23,606, all non-pregnant) who had plasma metabolome analysis results measured by NMR method (Supplementary Table S6)^30–32^. The Tohoku Medical Megabank Project (TMM) is underway in Miyagi and Iwate prefectures, comprising the TMM Community-Based Cohort Study (TMM CommCohort Study), an adult cohort study involving 80,000 participants, and the TMM Birth and Three-Generation Cohort Study (TMM BirThree Cohort Study) with 70,000 participants, encompassing pregnant individuals, their fetuses, parents, siblings, grandparents, and relatives. In this investigation, data analysis is restricted exclusively to the Tohoku University Tohoku Medical Megabank Organization (ToMMo), furthermore, as amino acid concentrations are known to change during pregnancy compared to non-pregnant females, pregnant females were excluded from the data analysis^44^. The period for these data measurements spanned from May 2013 to May 2017. A summary of the data is available from the “jMorp: Japanese Multi-Omics Reference Panel (released on September 29, 2022)”. The study was approved by the Ethics Committee of Tohoku Medical Megabank Organization (ToMMo), Tohoku University (approval number 2018-4-014; approved on July 24, 2013, approval number 2021-4-129; approved on January 22, 2021, approval number 2023-4-159; approved on November 7, 2016). Our studies were carried out in accordance with the approved guidelines. The written informed consent was obtained from all participants.

### NMR measurements and quality control

The details of the preparation of samples and NMR metabolome analysis following established protocols were already described in Koshiba et al.^27^. Plasma samples collected from cohort participants were stored at -80C. Metabolites were extracted from 200 μl of plasma samples using a standard methanol extraction procedure and were suspended in a sodium phosphate buffer. All NMR experiments were conducted at 298 K using a Bruker 600 MHz spectrometer (Bruker BioSpin, Germany). Data analysis was performed using the ChenomxNMRSuite (Chenomx, Edmonton, Canada). Automatic metabolite quantification was performed using in-house software.

Regarding quality control of samples, we examined the concentrations of certain metabolites (especially glucose and lactate) in each plasma sample and excluded the data if the sample quality was inadequate. First, we identified samples where the glucose concentration from NMR data was lower than that from blood tests (less than 0.7), and lactate concentration was higher than average (more than 2SD). Then, after investigating the history of anomalies and identifying the cause of an anomaly, those samples were excluded from the database (Koshiba et al.^27^, Saigusa et al.^45^). Before analysis, samples (n = 1290) where the days from blood collection to processing and storage were on the same day (hereafter called “DateDiff0”) or the following day (hereafter called “DateDiff1”) were excluded, and only high-quality samples were used for analysis. Furthermore, among the 45 measured metabolites, missing values were removed for each metabolite, and metabolites that did not meet the criteria of covering 80% of all subjects (Caffeine: NA:8748, Hypoxanthine: NA:22,840, Inosine: NA:36,876) were excluded from the analysis. Notably, all samples with DateDiff1 or more were excluded as missing values for Hypoxanthine and Inosine. Consequently, the final analysis included 39,239 subjects (Age; Mean 56.68, SD 13.84, male: n = 16,434, female: n = 22,805), and 42 metabolites were analyzed (Fig. 1a).

### Age grouping

The age is calculated at the blood collection date (Range 20.0–91.0, 1st quantile 46, Median 61, 3rd quantile 67). To create metabolic markers for each age, we decided to group the ages into several-year intervals and use their summary values as representative values for the indicators. We divided ages into the following 13 groups (group1: 20–24, group2: 25–29, group3: 30–34, group4: 35–39, group5: 40–44, group6: 45–49, group7: 50–54, group8: 55–59, group9: 60–64, group10: 65–69, group11: 70–74, group12: 75–79, group13: over80), where we classified over 80 s into one to prevent the number of people from being too small compared to other groups. Also, looking at the distribution of the number of people in Fig. 1b, the 60 s age group is significantly larger. In this case, there is an advantage that finer features such as patterns and peaks of concentration changes can be easily grasped before the older age when metabolic diseases increase, especially in the range from 60 to 75 years old, by calculating the values in 5-year intervals. Also, to estimate the age at which changes in metabolites occur, we determined that a 5-year interval was appropriate with the effect of equalizing the number of people in each group as much as possible when comparing the difference in metabolite concentration with the neighboring group and eliminating extreme differences in sample size.

### Body mass index

BMI group as an internal factor other than age, it has been reported that body shape (obesity) affects metabolites^21,35,37^. Therefore, we divided into three groups of low, medium, and high using the BMI value and examined the effect of BMI on metabolites. The BMI values used for the analysis were calculated by dividing body weight (Kg) by height (m) squared. The TMM cohort study has two cohort studies, each recruiting participants through multiple approaches^30–32^, so the items surveyed may vary depending on the participant. The height and weight values used to calculate BMI were measured at Community Support Centers. If there were no measured values, we used the BMI calculated from the self-reported values on the survey form, and if these values were missing, they were excluded from the analysis. Therefore, the number of people analyzed was n = 28,083 (male: n = 11,904, female: n = 16,179).

Regarding outliers, since a high BMI does not necessarily mean obesity (for example, in the case of a large amount of muscle mass), we did not establish an exclusion criterion based on the definition of abnormal values, and all values were analyzed by introducing stratification into three groups. For the cut-off values for stratification, the distribution and definition of BMI vary by country, so we used quartiles for each data set and defined less than the 1st quantile as the Low group, more than the 1st quantile but less than the 3rd quantile as the Middle group, and more than the 3rd quantile as the High group (Supplementary Table S7-1,2).

### Time after the last meals

The difference in time from the last meal to blood collection is a well-known factor that affects metabolite concentrations. Thus, we examined the effect of meals on metabolites (Hereafter, we refer to it as the postprandial time). It is not easy to define postprandial time uniformly because the survey items and locations differ for each participant. Still, we followed the discriminant method created in the regional residents’ cohort survey^32^. Specifically, people who have a continuous value for postprandial time were divided into two groups: “any time (less than 10 h)” and “empty stomach (10 h or more)”. If neither of this information was recorded, it was considered missing data and excluded from the analysis. As a result, the number of people analyzed was n = 26,056 (male: empty stomach n = 3168, any time n = 8225, Total n = 11,393, female: empty stomach n = 5226, any time n = 9437, Total n = 14,663).

### Statistical analyses

Although metabolite data often exhibit a ‘positive skewness’ with a distribution that drags on the right side or can follow a log-normal distribution, and sometimes we verify after log transformation, here we decided that there is significance in using the mean as an indicator of metabolite concentration summary values in age groups. We used a one-sample t-test (mean test) and corresponding interval estimation to support this approach. After that, we describe the mean and its 95% confidence interval as the estimation results. Additionally, we completed the age index table by concurrently listing standard deviations, medians, and quartiles.

Other tests used included the Kolmogorov–Smirnov Test (K–S Test) for normality, with a significance level of less than 5% (*p* < 0.05). For testing differences between groups, we applied nonparametric methods with high robustness, which are not influenced by the shape of the distribution. The statistical significance test for metabolite concentration differences between age groups was conducted using the Wilcoxon ranK–Sum test. The Bonferroni method was used for multiple comparison procedures (adjustment for multiplicity). We adopted a significance level of adjusted *p* < 0.05. Further, for the analysis between age groups, effect size, and 95% confidence intervals were calculated and listed for all tests. The effect size index adopted was Cliff’s delta (calculated by Z-transforming the test statistic) in non-parametric tests, calculated for each group combination. The calculations used the “effsize” package in R, and the interpretation standards for Cliff’s delta were adopted as “negligible < 0.147, small size < 0.330, medium size < 0.474, large size > 0.474”, which are general interpretation standards in “effsize”.

Finally, we verified the relationship between the variables of “BMI” or “postprandial time”, “age”, and “metabolite concentration” by conducting multiple regression analyses with each metabolite concentration as the dependent variable. At that time, as the number of people used for analysis differed between “BMI” and “postprandial time,” they were analyzed separately. The independent variable used was the continuous value of age. BMI values were divided into three groups: low, intermediate, and high, and the intermediate and high groups were used for analysis, setting the “intermediate group” as the base of the two-level categorical variable. Also, when the independent variables were age and postprandial time, postprandial time was divided into two levels: Empty stomach (10 h or more) and any time (less than 10 h), here setting the “Empty stomach group” as the base of the categorical variable. To avoid multicollinearity, age was centered, and interaction terms of age × BMI and age × postprandial time were introduced. Statistical analyses were conducted using R version 4.1.2.

### Supplementary Information

Supplementary Information.

## Data Availability

Summary statistics are publicly available at the Japanese Multi Omics Reference Panel website (URL: https://jmorp.megabank.tohoku.ac.jp/). Individual metabolite data used for the study are available upon request after approval of the Ethical Committee and the Materials and Information Distribution Review Committee of Tohoku Medical Megabank Organization. In response to reasonable requests for these data (contact us at dist@megabank.tohoku.ac.jp), we will share the stored data after assembling the data set after approval of the Ethics Committee and the Materials and Information Distribution Review Committee of Tohoku Medical Megabank Organization.
