# Supplementary material for: Identifying critical age and gender-based metabolomic shifts in a Japanese population of the Tohoku Medical Megabank cohort
Source: Sci Rep. 2024 Jul 8;14:15681. doi: 10.1038/s41598-024-66180-0 (PMC11231361; doi:10.1038/s41598-024-66180-0)
Supplement: Supplementary file 1 — Supplementary Information. [file 41598_2024_66180_MOESM1_ESM.pdf]

Supplementary Materials

Identifying critical age and gender-based metabolomic shifts in a Japanese population of the TMM cohort

Miyuki Sakurai, Ikuko N. Motoike, Eiji Hishinuma, Yuichi Aoki, Shu Tadaka, Mana Kogure, Masatsugu Orui, Mami Ishikuro, Taku Obara, Naoki Nakaya, Kazuki Kumada, Atsushi Hozawa, Shinichi Kuriyama, Masayuki Yamamoto, Seizo Koshiba, and Kengo Kinoshita

Supplementary Materials

CONTENTS

SUPPLEMENTARY FIGURE		Number of sheets
**Supplementary Figure S1**	Supplementary Figure S1-1 : male	2
	Supplementary Figure S1-2 : female	2
**Supplementary Figure S2**	Supplementary Figure S2-1 : male	1
	Supplementary Figure S2-2 : female	1

SUPPLEMENTARY FIGURE		Number of sheets
**Supplementary Table S1**	Supplementary Table S1	1
**Supplementary Table S2**	Supplementary Table S2-1(male)	7
	Supplementary Table S2-2(male)	7
	Supplementary Table S2-3(female)	7
	Supplementary Table S2-4(female)	7
**Supplementary Table S3**	Supplementary Table S3-1(male)	3
	Supplementary Table S3-2(female)	3
**Supplementary Table S4**	Supplementary Table S4-1(male)	2
	Supplementary Table S4-2(female)	1
**Supplementary Table S5**	Supplementary Table S5-1(male)	1
	Supplementary Table S5-2(female)	1
**Supplementary Table S6**	Supplementary Table S6	1
**Supplementary Table S7**	Supplementary Table S7-1(male)	1
	Supplementary Table S7-2(female)	

Supplementary Figure S1-1 : male (1/2)

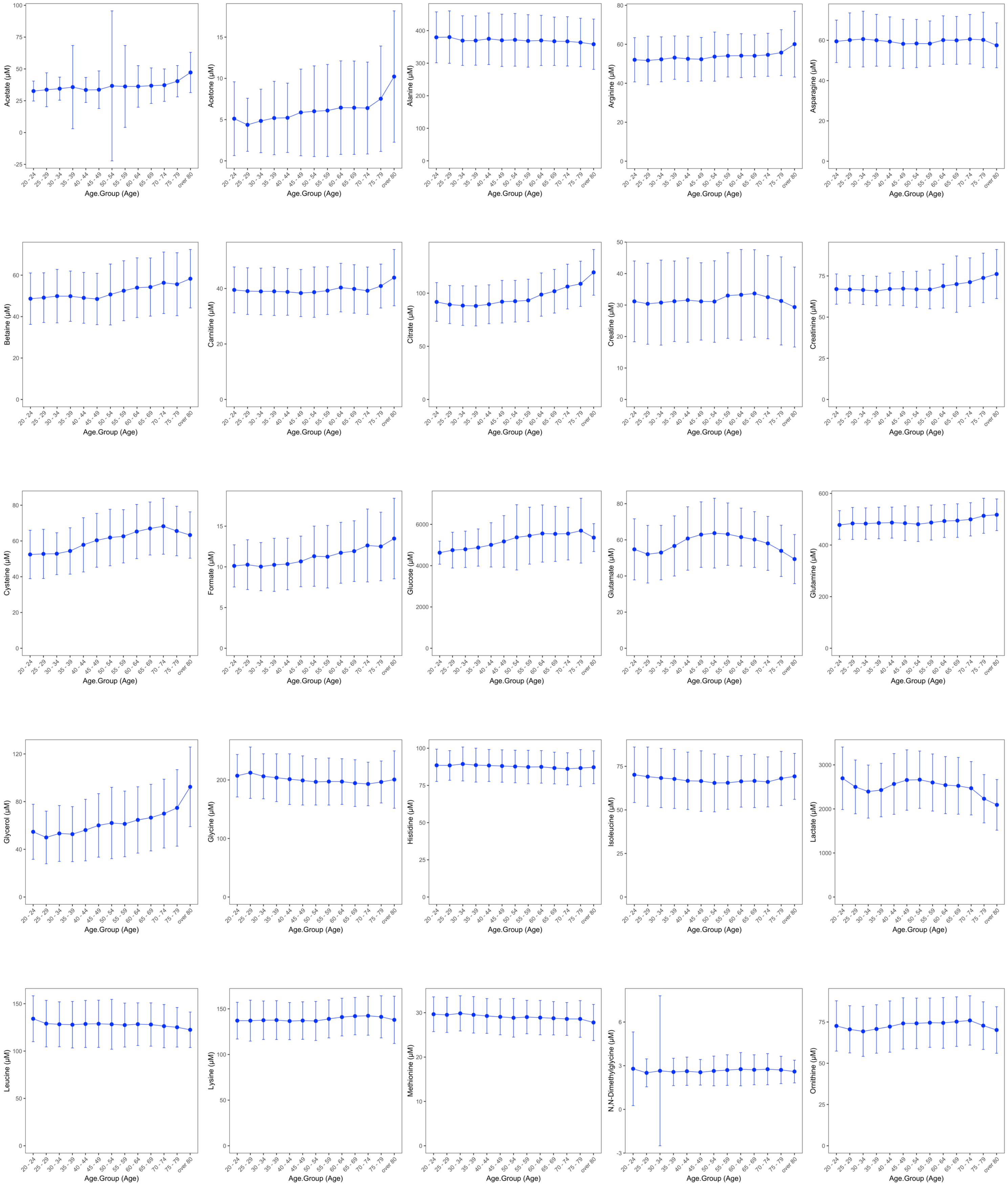

Supplementary Figure S1-1 : male (2/2)

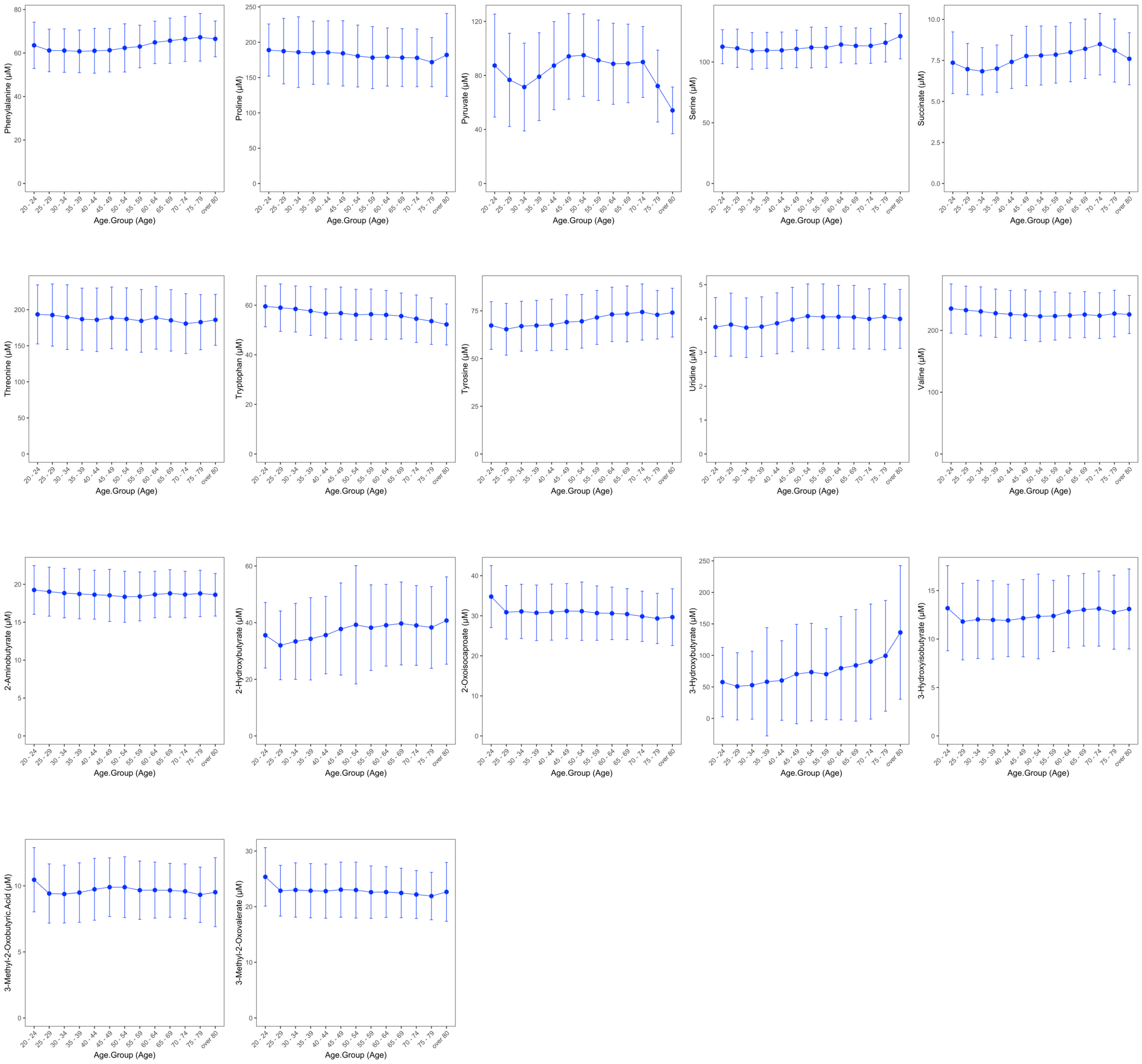

Supplementary Figure S1-2 : female (1/2)

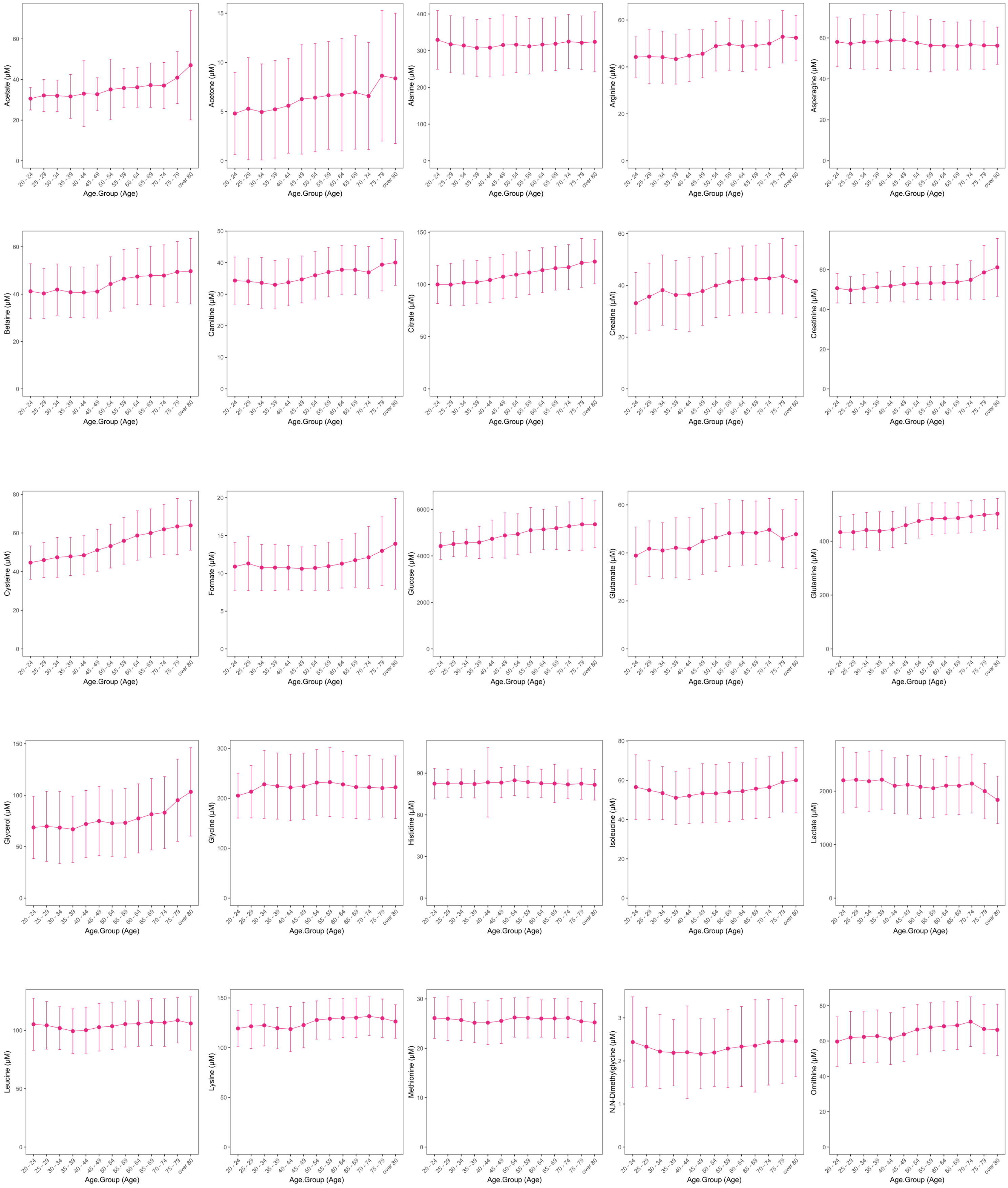

Supplementary Figure S1-2 : female (2/2)

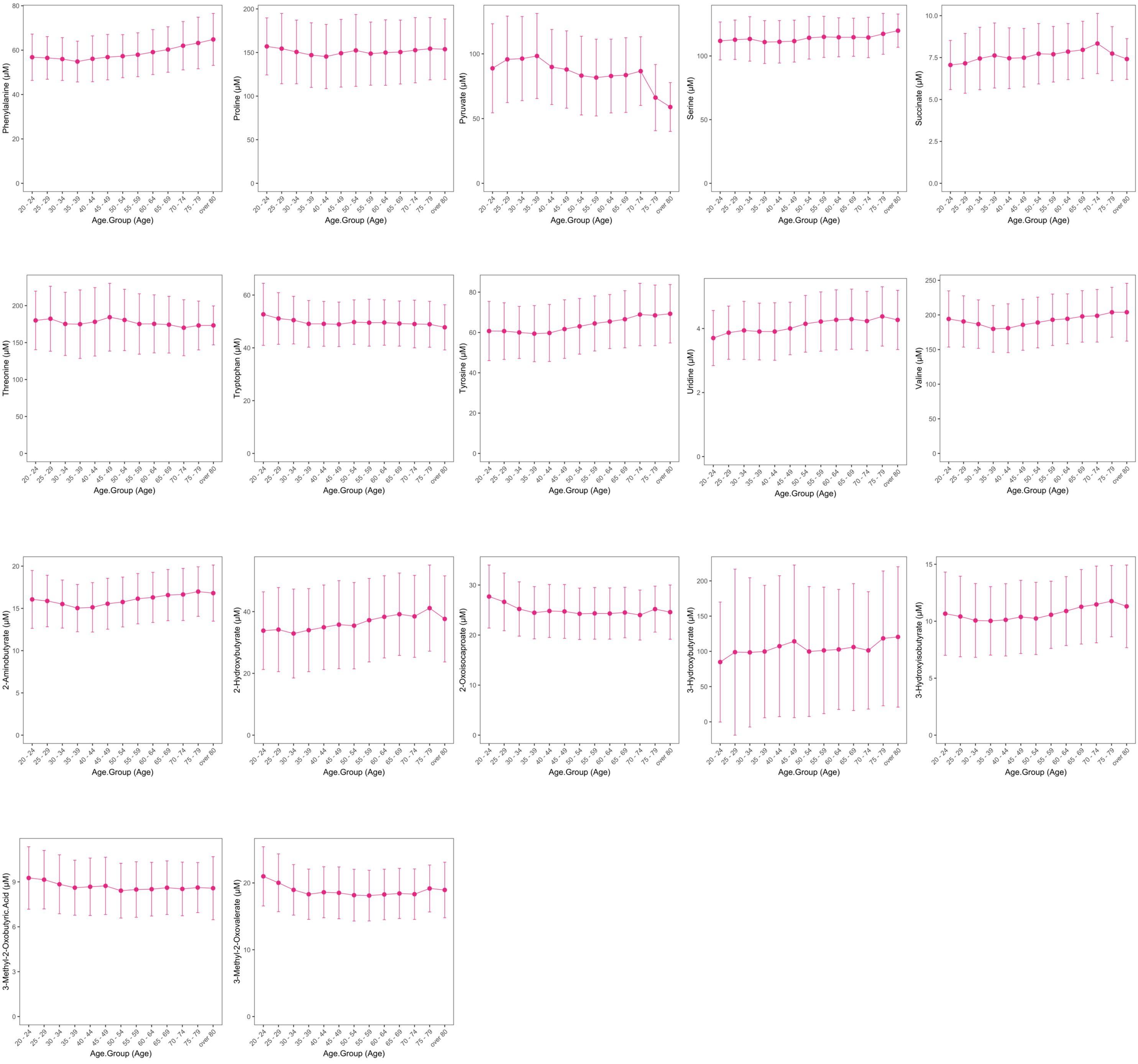

Supplementary Figure S1 : Mean values for 42 metabolites by age group (error bars are standard deviations). All graphs are values at DateDiff0, Supplementary Fig. S1-1 for males and Supplementary Fig. S1-2 for females.

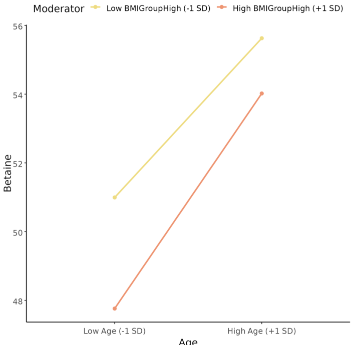

(A) Betaine

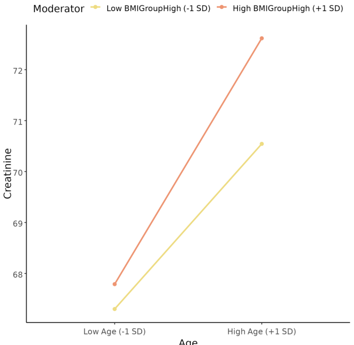

(B) Creatinine

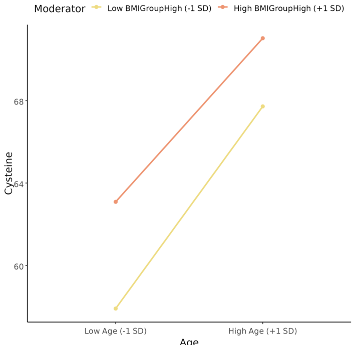

(C) Cysteine

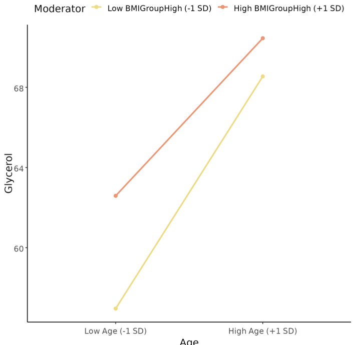

(D) Glycerol

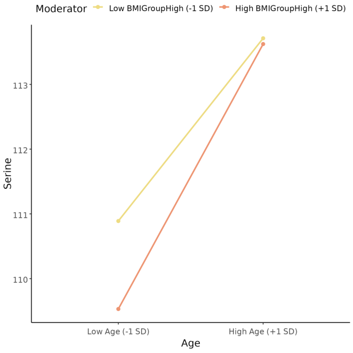

(E) Serine

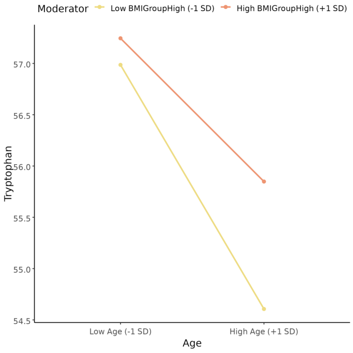

(F) Tryptophan

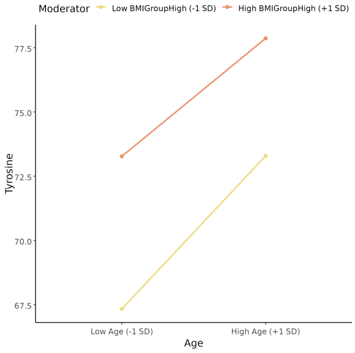

(G) Tyrosine

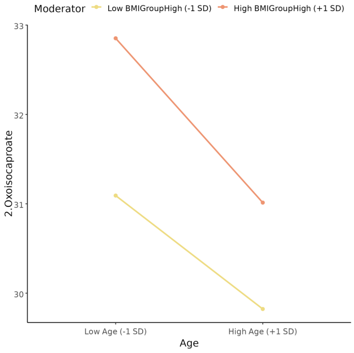

(H) 2-Oxoisocaproate

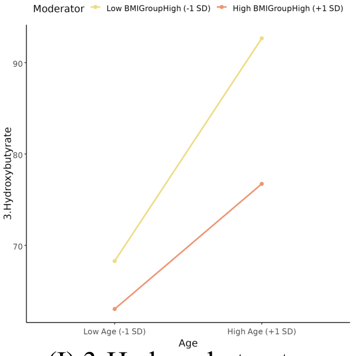

(I) 3-Hydroxybutyrate

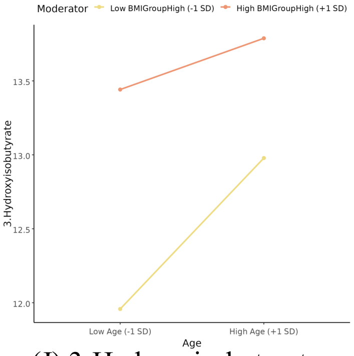

(J) 3-Hydroxyisobutyrate

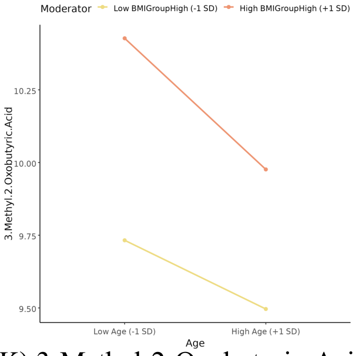

(K) 3-Methyl-2-Oxobutyric-Acid

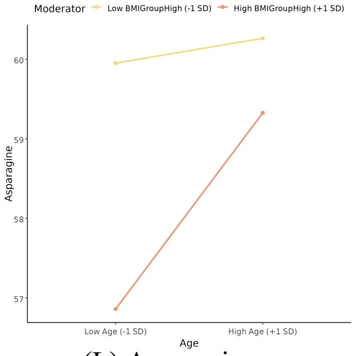

(L) Asparagine

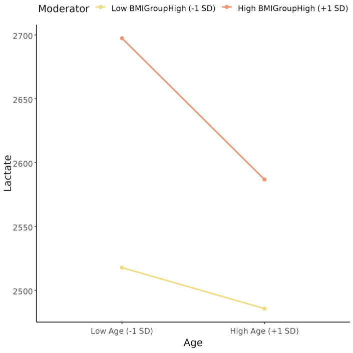

(M) Lactate

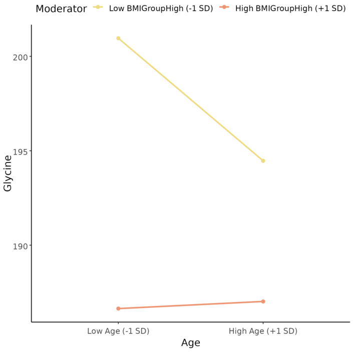

(N) Glycine

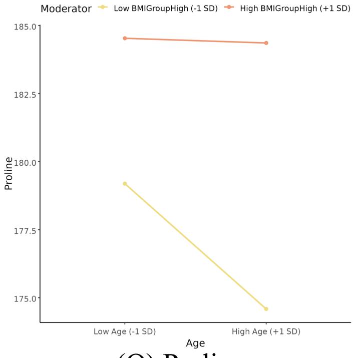

(O) Proline

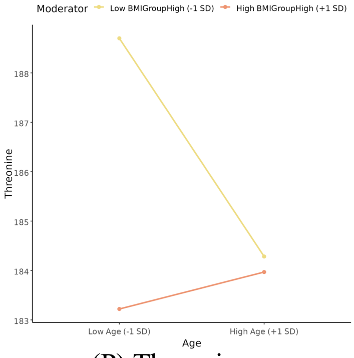

(P) Threonine

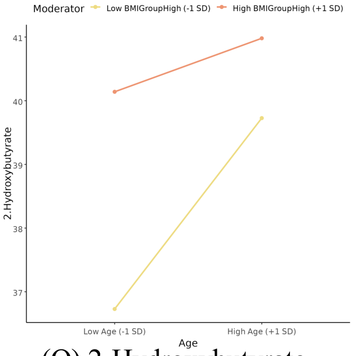

(Q) 2-Hydroxybutyrate

Supplementary Figure S2-1 : male

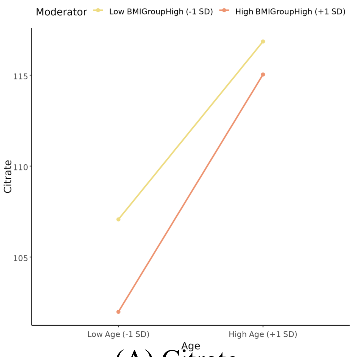

(A) Citrate

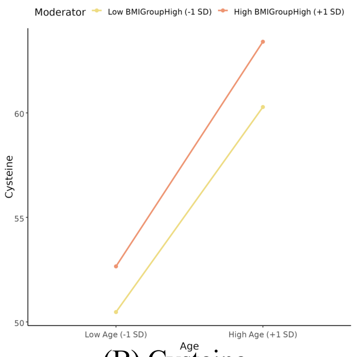

(B) Cysteine

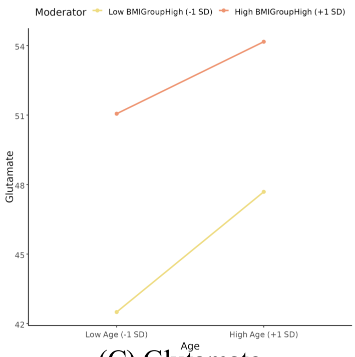

(C) Glutamate

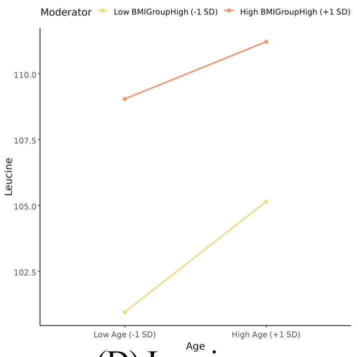

(D) Leucine

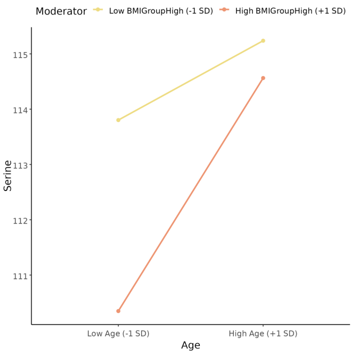

(E) Serine

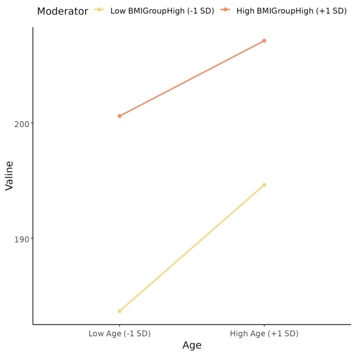

(F) Valine

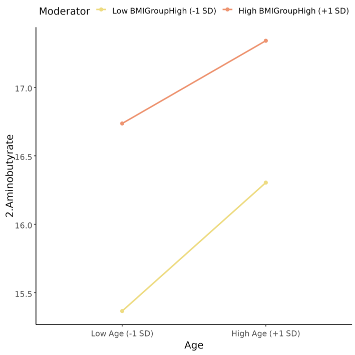

(G) 2-Aminobutyrate

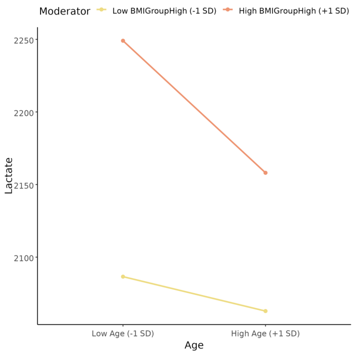

(H) Lactate

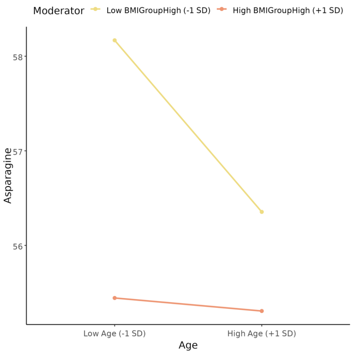

(I) Asparagine

Supplementary Figure S2-2 : female

Supplementary Figure S2 : Age and BMI interaction effects on metabolites. Values of metabolite concentrations when taking high/low (±1SD) age and high/low (±1SD) BMI values are shown. Supplementary Fig. S2-1 is for males and Supplementary Fig. S2-2 is for females. Supplementary Fig. S2-1 (A)-(K) show significant differences across all BMI ranges. (L)(M) showed differences only in the high BMI range; (N)-(Q) showed significant differences only in the low BMI range. Supplementary Fig. S2-2 (A)-(G) showed significant differences in all BMI ranges. (H) showed differences only in the high BMI range and (I) showed significant differences only in the low BMI range.

Supplementary Table S1 : Overview of DateDiff0 and DateDiff1

Name	Total	DateDiff		Mean	SD	ks.p.value	wilcox. p.value	DateDiff0. Mean	DateDiff1. Mean	DateDiff0. SD	DateDiff1. SD	DateDiff0. CV	DateDiff1. CV	Diff0 - Diff1. Mean	Diff0 - Diff1. SD	Diff0 .CV- Diff1.CV
	N	DateDiff0. N	Date Diff1. N													
Acetate	39172	29228	9944	34.679	17.262	0.0000	0.0000	35.716	31.630	17.245	16.950	0.483	0.536	4.086	0.295	-0.053
Acetone	39232	29283	9949	6.166	5.312	0.0000	0.0000	6.244	5.938	5.446	4.891	0.872	0.824	0.306	0.555	0.049
Alanine	39239	29290	9949	337.515	79.024	0.0000	0.0000	339.708	331.059	80.603	73.805	0.237	0.223	8.649	6.798	0.014
Arginine	39236	29288	9948	50.700	11.498	0.0000	0.0000	50.541	51.166	11.436	11.669	0.226	0.228	-0.625	-0.233	-0.002
Asparagine	39231	29285	9946	57.887	12.599	0.0000	0.0000	58.206	56.948	12.527	12.761	0.215	0.224	1.258	-0.234	-0.009
Betaine	39236	29288	9948	48.668	13.578	0.0000	0.0000	48.695	48.590	13.612	13.479	0.280	0.277	0.105	0.133	0.002
Carnitine	39029	29166	9863	37.991	8.548	0.0000	0.0000	37.689	38.885	8.407	8.893	0.223	0.229	-1.196	-0.486	-0.006
Citrate	39238	29289	9949	105.822	22.782	0.0000	0.0000	105.094	107.966	22.643	23.053	0.215	0.214	-2.872	-0.410	0.002
Creatine	39239	29290	9949	37.282	14.102	0.0000	0.0000	36.977	38.182	14.066	14.170	0.380	0.371	-1.205	-0.104	0.009
Creatinine	39222	29276	9946	59.776	13.546	0.0000	0.0000	60.003	59.105	13.500	13.658	0.225	0.231	0.898	-0.158	-0.006
Cysteine	39238	29289	9949	57.790	14.645	0.0000	0.0000	58.937	54.411	14.648	14.104	0.249	0.259	4.526	0.544	-0.011
Formate	39124	29178	9946	11.533	3.795	0.0000	0.0000	11.372	12.005	3.626	4.216	0.319	0.351	-0.633	-0.590	-0.032
Glucose	39217	29270	9947	4991.337	1142.178	0.0000	0.0000	5163.718	4484.089	1115.532	1066.282	0.216	0.238	679.629	49.250	-0.022
Glutamate	39238	29289	9949	52.840	16.591	0.0000	0.0000	52.253	54.569	16.195	17.594	0.310	0.322	-2.316	-1.399	-0.012
Glutamine	39239	29290	9949	482.373	64.493	0.0000	0.0000	481.810	484.032	64.925	63.177	0.135	0.131	-2.222	1.748	0.004
Glycerol	39239	29290	9949	72.143	32.832	0.0000	0.0000	70.591	76.714	32.481	33.431	0.460	0.436	-6.123	-0.950	0.024
Glycine	39239	29290	9949	213.972	57.495	0.0000	0.0000	213.277	216.018	57.313	57.984	0.269	0.268	-2.741	-0.671	0.000
Histidine	39225	29279	9946	84.572	12.416	0.0000	0.0000	84.875	83.682	12.089	13.293	0.142	0.159	1.193	-1.204	-0.016
Isoleucine	39239	29290	9949	60.442	16.427	0.0000	0.0000	59.823	62.265	16.391	16.396	0.274	0.263	-2.442	-0.005	0.011
Lactate	39239	29290	9949	2512.434	753.424	0.0000	0.0000	2290.830	3164.838	625.286	719.433	0.273	0.227	-874.008	-94.147	0.046
Leucine	39239	29290	9949	115.807	24.335	0.0000	0.0000	114.873	118.556	24.464	23.739	0.213	0.200	-3.683	0.725	0.013
Lysine	39239	29290	9949	131.442	21.777	0.0000	0.0000	132.865	127.255	21.811	21.131	0.164	0.166	5.61	0.680	-0.002
Methionine	39239	29290	9949	26.801	4.260	0.0000	0.0000	27.257	25.458	4.277	3.914	0.157	0.154	1.799	0.363	0.003
N.N-Dimethylglycine	38941	29027	9914	2.488	1.742	0.0000	0.0000	2.472	2.534	1.336	2.585	0.540	1.020	-0.062	-1.249	-0.480
Ornithine	39239	29290	9949	68.653	15.046	0.0000	0.0000	70.003	64.679	15.192	13.864	0.217	0.214	5.324	1.328	0.003
Phenylalanine	39239	29290	9949	61.067	10.737	0.0000	0.0000	60.988	61.298	10.728	10.760	0.176	0.176	-0.31	-0.032	0.000
Proline	39236	29288	9948	164.715	42.960	0.0000	0.0000	163.539	168.178	42.817	43.194	0.262	0.257	-4.639	-0.377	0.005
Pyruvate	39238	29290	9948	75.881	32.698	0.0000	0.0000	86.375	44.986	30.243	15.666	0.350	0.348	41.389	14.577	0.002
Serine	39180	29242	9938	112.458	16.057	0.0000	0.0000	113.205	110.261	15.693	16.895	0.139	0.153	2.944	-1.202	-0.015
Succinate	39238	29290	9948	8.019	1.812	0.0000	0.0000	7.833	8.565	1.793	1.755	0.229	0.205	-0.732	0.038	0.024
Threonine	39239	29290	9949	181.305	41.753	0.0000	0.0000	180.140	184.736	42.160	40.340	0.234	0.218	-4.596	1.820	0.016
Tryptophan	39238	29289	9949	52.204	9.771	0.0000	0.0000	52.364	51.732	9.807	9.650	0.187	0.187	0.632	0.157	0.001
Tyrosine	39238	29289	9949	67.135	14.723	0.0000	0.0000	67.488	66.097	14.821	14.381	0.220	0.218	1.391	0.440	0.002
Uridine	38629	28766	9863	4.154	0.954	0.0000	0.0000	4.075	4.386	0.932	0.981	0.229	0.224	-0.311	-0.049	0.005
Valine	39239	29290	9949	207.924	40.870	0.0000	0.0000	206.911	210.905	40.995	40.354	0.198	0.191	-3.994	0.641	0.007
2-Aminobutyrate	39239	29290	9949	17.292	3.346	0.0000	0.0000	17.222	17.499	3.353	3.316	0.195	0.189	-0.277	0.037	0.005
2-Hydroxybutyrate	39237	29288	9949	37.879	14.495	0.0000	0.0000	37.423	39.220	14.286	15.014	0.382	0.383	-1.797	-0.728	-0.001
2-Oxoisocaproate	39239	29290	9949	26.674	6.578	0.0000	0.0000	27.168	25.220	6.607	6.268	0.243	0.249	1.948	0.339	-0.005
3-Hydroxybutyrate	39233	29284	9949	94.221	90.294	0.0000	0.0000	91.328	102.735	89.042	93.370	0.975	0.909	-11.407	-4.328	0.066
3-Hydroxyisobutyrate	39239	29290	9949	11.418	3.623	0.0000	0.0000	11.581	10.938	3.647	3.510	0.315	0.321	0.643	0.137	-0.006
3-Methyl-2-Oxobutyric.Acid	39239	29290	9949	8.527	2.195	0.0000	0.0000	9.053	6.977	2.057	1.824	0.227	0.261	2.076	0.233	-0.034
3-Methyl-2-Oxovalerate	39239	29290	9949	20.249	4.648	0.0000	0.0000	20.273	20.178	4.694	4.509	0.232	0.223	0.095	0.185	0.008

Supplementary Table S1 : The entries in this table show the following information from left to right. Number of people (overall, DateDiff0, DateDiff1), mean, standard deviation, ks.p.value: p-value for normality test, wilcox.p.value : p-value for difference test between DateDiff0 and DateDiff1, mean, standard deviation and coefficient of variation for each DateDiff group, Difference in mean, standard deviation and coefficient of variation for DateDiff0 minus DateDiff1.

Supplementary Table S2-1 : DateDiff0 male 20’s
(1/7)

DateDiff0 male	20 - 24						25 - 29					
Name	N	Mean	95%CI	SD	Median	Quantile [lower, upper]	N	Mean	95%CI	SD	Median	Quantile [lower, upper]
Acetate	123	32.610	[31.210 , 34.010]	7.844	31.030	[26.955 , 35.910]	363	33.654	[32.280 , 35.028]	13.311	31.560	[28.275 , 36.205]
Acetone	123	5.107	[4.310 , 5.904]	4.465	3.940	[2.735 , 5.885]	363	4.365	[4.033 , 4.697]	3.219	3.430	[2.360 , 5.265]
Alanine	123	379.419	[365.467 , 393.371]	78.167	380.740	[326.585 , 423.585]	363	380.085	[371.772 , 388.398]	80.544	377.330	[324.660 , 434.920]
Arginine	123	52.050	[50.023 , 54.076]	11.353	51.620	[45.815 , 59.005]	363	51.724	[50.441 , 53.008]	12.436	50.840	[44.115 , 57.690]
Asparagine	123	59.477	[57.594 , 61.360]	10.551	58.870	[51.570 , 67.035]	363	60.129	[58.739 , 61.520]	13.472	58.390	[50.050 , 67.590]
Betaine	123	48.653	[46.438 , 50.868]	12.407	47.470	[39.660 , 56.890]	363	49.150	[47.913 , 50.387]	11.987	47.540	[40.420 , 56.130]
Carnitine	123	39.495	[38.021 , 40.970]	8.261	38.610	[34.520 , 45.515]	362	39.056	[38.187 , 39.925]	8.410	38.915	[33.355 , 44.445]
Citrate	123	91.733	[88.482 , 94.984]	18.213	89.990	[79.235 , 106.810]	363	89.283	[87.434 , 91.133]	17.919	87.030	[78.030 , 98.200]
Creatine	123	31.172	[28.882 , 33.462]	12.830	27.820	[21.560 , 38.135]	363	30.407	[29.079 , 31.735]	12.867	27.270	[21.245 , 36.355]
Creatinine	123	67.015	[65.373 , 68.656]	9.197	66.680	[60.575 , 72.090]	363	66.787	[65.936 , 67.638]	8.247	66.770	[61.605 , 71.485]
Cysteine	123	52.453	[50.036 , 54.870]	13.540	51.820	[43.385 , 58.930]	363	52.762	[51.341 , 54.182]	13.759	51.190	[44.725 , 59.445]
Formate	121	10.120	[9.653 , 10.587]	2.593	9.830	[8.560 , 11.750]	360	10.270	[9.952 , 10.587]	3.060	9.970	[8.328 , 11.565]
Glucose	123	4626.419	[4526.909 , 4725.930]	557.499	4551.130	[4273.895 , 4869.540]	363	4750.731	[4661.466 , 4839.996]	864.837	4666.840	[4247.265 , 5146.315]
Glutamate	123	54.751	[51.737 , 57.765]	16.887	51.320	[42.435 , 64.475]	363	52.062	[50.416 , 53.709]	15.953	49.360	[40.165 , 61.265]
Glutamine	123	477.718	[467.811 , 487.626]	55.508	477.840	[440.575 , 511.755]	363	483.696	[477.305 , 490.087]	61.916	481.970	[443.060 , 522.400]
Glycerol	123	54.696	[50.576 , 58.816]	23.080	50.590	[40.285 , 62.630]	363	49.956	[47.677 , 52.234]	22.074	44.490	[34.885 , 61.215]
Glycine	123	207.240	[200.767 , 213.713]	36.264	200.630	[180.910 , 226.485]	363	212.348	[207.818 , 216.877]	43.880	207.500	[182.305 , 232.665]
Histidine	123	88.521	[86.583 , 90.460]	10.860	88.780	[80.955 , 94.365]	363	88.439	[87.410 , 89.467]	9.963	87.710	[81.180 , 94.160]
Isoleucine	123	70.180	[67.340 , 73.021]	15.912	68.890	[59.445 , 78.935]	363	69.098	[67.346 , 70.851]	16.979	66.020	[57.560 , 77.475]
Lactate	123	2695.663	[2569.032 , 2822.294]	709.439	2649.800	[2207.265 , 3121.765]	363	2500.545	[2437.569 , 2563.522]	610.141	2438.860	[2044.445 , 2887.120]
Leucine	123	134.132	[129.810 , 138.454]	24.213	133.530	[118.475 , 147.860]	363	128.980	[126.445 , 131.514]	24.553	125.650	[112.310 , 141.070]
Lysine	123	137.077	[133.487 , 140.667]	20.111	135.890	[124.800 , 147.910]	363	137.059	[134.745 , 139.373]	22.417	134.890	[121.825 , 147.660]
Methionine	123	29.664	[28.964 , 30.364]	3.921	29.470	[27.550 , 31.975]	363	29.518	[29.109 , 29.926]	3.959	29.460	[27.040 , 31.485]
N,N-Dimethylglycine	123	2.790	[2.339 , 3.241]	2.528	2.580	[1.975 , 3.120]	360	2.507	[2.407 , 2.607]	0.964	2.340	[1.860 , 2.960]
Ornithine	123	72.606	[69.888 , 75.324]	15.229	70.200	[61.095 , 82.570]	363	70.551	[69.077 , 72.026]	14.286	69.900	[60.570 , 78.765]
Phenylalanine	123	63.541	[61.638 , 65.445]	10.662	62.330	[54.920 , 71.550]	363	61.169	[60.158 , 62.179]	9.793	60.120	[54.960 , 65.610]
Proline	123	188.937	[182.347 , 195.528]	36.924	183.570	[163.435 , 207.295]	363	187.400	[182.613 , 192.186]	46.373	178.700	[157.465 , 207.975]
Pyruvate	123	87.326	[80.519 , 94.132]	38.134	83.530	[57.810 , 112.370]	363	76.708	[73.142 , 80.274]	34.548	71.120	[50.170 , 97.080]
Serine	123	112.537	[110.040 , 115.033]	13.985	113.640	[101.945 , 121.375]	362	111.262	[109.635 , 112.889]	15.741	110.140	[101.305 , 120.235]
Succinate	123	7.362	[7.026 , 7.697]	1.880	7.130	[6.115 , 8.380]	363	6.967	[6.806 , 7.129]	1.561	6.760	[5.845 , 8.035]
Threonine	123	193.539	[186.233 , 200.845]	40.932	185.940	[163.420 , 219.320]	363	192.584	[188.141 , 197.027]	43.047	190.520	[163.260 , 220.670]
Tryptophan	123	59.540	[58.067 , 61.013]	8.251	58.600	[53.185 , 63.545]	363	59.002	[58.016 , 59.987]	9.550	58.160	[52.745 , 64.985]
Tyrosine	123	67.315	[65.084 , 69.546]	12.499	66.240	[57.835 , 76.625]	363	65.370	[63.970 , 66.769]	13.559	62.650	[56.290 , 71.380]
Uridine	120	3.753	[3.596 , 3.910]	0.868	3.705	[3.112 , 4.325]	349	3.823	[3.725 , 3.921]	0.933	3.750	[3.190 , 4.320]
Valine	123	235.278	[228.181 , 242.374]	39.760	228.550	[210.570 , 258.220]	363	232.691	[228.677 , 236.706]	38.893	226.470	[206.085 , 251.820]
2-Aminobutyrate	123	19.251	[18.678 , 19.825]	3.213	18.920	[17.320 , 21.230]	363	19.030	[18.697 , 19.363]	3.225	18.600	[16.705 , 20.750]
2-Hydroxybutyrate	123	35.547	[33.480 , 37.614]	11.582	34.560	[26.465 , 42.840]	363	31.983	[30.729 , 33.236]	12.144	28.890	[23.385 , 38.665]
2-Oxoisocaproate	123	34.775	[33.396 , 36.154]	7.726	34.920	[29.765 , 40.270]	363	30.878	[30.187 , 31.568]	6.692	30.620	[25.920 , 34.810]
3-Hydroxybutyrate	123	57.689	[47.869 , 67.509]	55.014	39.880	[29.805 , 63.915]	363	50.859	[45.355 , 56.363]	53.324	34.770	[27.615 , 48.870]
3-Hydroxyisobutyrate	123	13.180	[12.397 , 13.963]	4.386	12.610	[10.200 , 15.135]	363	11.799	[11.391 , 12.207]	3.954	11.250	[8.970 , 13.700]
3-Methyl-2-Oxobutyric Acid	123	10.464	[10.030 , 10.899]	2.434	10.630	[8.985 , 11.955]	363	9.418	[9.186 , 9.649]	2.244	9.150	[7.790 , 11.015]
3-Methyl-2-Oxovalerate	123	25.362	[24.425 , 26.298]	5.245	25.050	[21.660 , 28.765]	363	22.867	[22.394 , 23.339]	4.574	22.580	[19.735 , 25.765]

Supplementary Table S2-1 : DateDiff0 male 30’s
(2/7)

DateDiff0 male	30 - 34						35 - 39					
Name	N	Mean	95%CI	SD	Median	Quantile [lower, upper]	N	Mean	95%CI	SD	Median	Quantile [lower, upper]
Acetate	867	34.476	[33.872 , 35.080]	9.062	32.870	[28.705 , 38.475]	940	35.698	[33.605 , 37.792]	32.702	32.485	[28.280 , 37.875]
Acetone	869	4.840	[4.584 , 5.096]	3.845	3.810	[2.600 , 5.770]	942	5.186	[4.902 , 5.470]	4.447	3.930	[2.560 , 6.295]
Alanine	869	369.310	[364.233 , 374.386]	76.250	367.120	[313.620 , 419.050]	942	369.448	[364.612 , 374.285]	75.641	364.770	[316.668 , 417.052]
Arginine	869	52.279	[51.512 , 53.045]	11.515	51.630	[44.950 , 58.780]	942	53.150	[52.439 , 53.862]	11.130	52.355	[46.133 , 59.960]
Asparagine	869	60.621	[59.701 , 61.542]	13.823	58.990	[51.620 , 67.740]	942	60.022	[59.199 , 60.845]	12.865	58.725	[51.340 , 67.030]
Betaine	869	49.888	[49.030 , 50.747]	12.895	48.230	[41.150 , 56.620]	942	49.849	[49.071 , 50.626]	12.154	48.155	[41.192 , 56.763]
Carnitine	868	38.953	[38.394 , 39.511]	8.381	38.400	[32.943 , 44.975]	940	38.965	[38.409 , 39.521]	8.687	37.890	[32.720 , 43.930]
Citrate	869	88.324	[87.083 , 89.565]	18.639	85.730	[75.830 , 98.160]	942	87.991	[86.790 , 89.192]	18.782	86.285	[75.240 , 98.735]
Creatine	869	30.780	[29.881 , 31.679]	13.507	27.910	[21.280 , 36.520]	942	31.203	[30.385 , 32.020]	12.786	27.975	[21.705 , 37.325]
Creatinine	869	66.482	[65.896 , 67.068]	8.801	66.100	[60.510 , 72.010]	942	65.898	[65.327 , 66.469]	8.926	65.665	[59.752 , 71.205]
Cysteine	869	52.875	[52.095 , 53.655]	11.710	51.630	[45.100 , 59.170]	942	54.414	[53.585 , 55.242]	12.957	53.045	[46.150 , 60.517]
Formate	867	10.025	[9.827 , 10.222]	2.960	9.660	[8.070 , 11.640]	933	10.249	[10.039 , 10.458]	3.261	9.710	[8.110 , 11.730]
Glucose	869	4793.079	[4734.504 , 4851.654]	879.766	4684.750	[4339.840 , 5064.300]	942	4876.737	[4819.160 , 4934.315]	900.477	4740.945	[4402.587 , 5193.925]
Glutamate	869	52.960	[51.957 , 53.963]	15.065	50.930	[41.940 , 61.710]	942	56.624	[55.559 , 57.688]	16.644	54.210	[44.112 , 67.452]
Glutamine	869	483.099	[479.043 , 487.155]	60.922	483.460	[438.800 , 521.810]	942	485.418	[481.514 , 489.323]	61.057	482.200	[442.150 , 524.672]
Glycerol	869	53.302	[51.740 , 54.863]	23.453	47.810	[37.050 , 64.470]	942	52.731	[51.256 , 54.207]	23.074	46.940	[36.570 , 62.710]
Glycine	869	206.249	[203.690 , 208.808]	38.431	203.590	[178.770 , 227.750]	942	203.839	[201.228 , 206.450]	40.834	197.595	[176.908 , 223.165]
Histidine	869	89.404	[88.646 , 90.162]	11.389	88.060	[81.730 , 95.660]	942	88.640	[87.911 , 89.368]	11.392	87.740	[81.028 , 95.095]
Isoleucine	869	68.327	[67.196 , 69.458]	16.990	65.140	[56.930 , 76.270]	942	67.780	[66.698 , 68.863]	16.928	64.680	[56.675 , 75.315]
Lactate	869	2394.215	[2354.133 , 2434.298]	602.018	2313.660	[1963.300 , 2753.350]	942	2428.743	[2389.926 , 2467.560]	607.073	2371.315	[1985.257 , 2789.400]
Leucine	869	128.293	[126.715 , 129.870]	23.689	124.960	[112.730 , 141.340]	942	127.837	[126.269 , 129.404]	24.516	124.295	[111.190 , 141.395]
Lysine	869	137.499	[136.096 , 138.901]	21.064	136.670	[122.890 , 150.110]	942	137.645	[136.285 , 139.005]	21.270	136.735	[123.615 , 148.857]
Methionine	869	29.854	[29.591 , 30.118]	3.958	29.410	[27.370 , 31.840]	942	29.529	[29.267 , 29.792]	4.106	29.130	[26.885 , 31.538]
N,N-Dimethylglycine	861	2.653	[2.309 , 2.997]	5.145	2.350	[1.870 , 2.950]	936	2.568	[2.508 , 2.628]	0.940	2.415	[1.958 , 3.010]
Ornithine	869	69.322	[68.314 , 70.330]	15.138	68.510	[58.240 , 79.010]	942	70.771	[69.833 , 71.709]	14.666	69.795	[59.625 , 79.245]
Phenylalanine	869	61.123	[60.458 , 61.788]	9.986	59.830	[53.990 , 66.400]	942	60.768	[60.142 , 61.394]	9.789	59.600	[53.460 , 66.702]
Proline	868	185.920	[182.585 , 189.255]	50.061	177.380	[153.812 , 205.315]	942	185.070	[182.208 , 187.933]	44.768	176.900	[153.628 , 207.955]
Pyruvate	869	71.403	[69.237 , 73.569]	32.527	65.350	[48.180 , 90.790]	942	79.049	[76.969 , 81.129]	32.529	76.060	[53.585 , 101.715]
Serine	869	109.140	[108.138 , 110.142]	15.047	108.030	[99.020 , 117.710]	941	109.562	[108.612 , 110.511]	14.843	108.070	[99.730 , 119.560]
Succinate	869	6.840	[6.743 , 6.936]	1.442	6.680	[5.860 , 7.680]	942	7.004	[6.912 , 7.096]	1.440	6.905	[6.030 , 7.838]
Threonine	869	189.706	[186.722 , 192.690]	44.818	186.080	[160.640 , 218.190]	942	186.906	[184.157 , 189.656]	43.000	186.730	[157.032 , 214.995]
Tryptophan	869	58.482	[57.863 , 59.101]	9.295	58.180	[52.040 , 63.780]	942	57.670	[57.040 , 58.300]	9.854	57.095	[51.812 , 62.870]
Tyrosine	869	66.962	[66.091 , 67.833]	13.079	65.520	[57.430 , 74.190]	942	67.315	[66.474 , 68.156]	13.150	65.750	[58.135 , 74.053]
Uridine	833	3.732	[3.672 , 3.792]	0.879	3.690	[3.090 , 4.350]	912	3.759	[3.702 , 3.816]	0.880	3.720	[3.138 , 4.332]
Valine	869	230.651	[228.011 , 233.290]	39.640	226.300	[203.770 , 252.120]	942	227.711	[225.210 , 230.212]	39.107	221.955	[202.050 , 249.177]
2-Aminobutyrate	869	18.839	[18.622 , 19.056]	3.261	18.620	[16.580 , 20.680]	942	18.727	[18.517 , 18.937]	3.284	18.345	[16.600 , 20.560]
2-Hydroxybutyrate	869	33.379	[32.487 , 34.272]	13.405	30.580	[23.990 , 39.050]	942	34.281	[33.353 , 35.209]	14.511	31.530	[25.210 , 40.295]
2-Oxoisocaproate	869	31.075	[30.624 , 31.525]	6.771	30.220	[26.600 , 35.100]	942	30.742	[30.299 , 31.185]	6.931	30.160	[26.093 , 34.745]
3-Hydroxybutyrate	869	52.794	[49.206 , 56.382]	53.894	36.600	[29.550 , 51.490]	942	58.145	[52.650 , 63.641]	85.951	38.090	[30.375 , 53.862]
3-Hydroxyisobutyrate	869	12.022	[11.753 , 12.291]	4.039	11.240	[9.300 , 13.780]	942	11.972	[11.713 , 12.230]	4.043	11.310	[9.362 , 13.850]
3-Methyl-2-Oxobutyric Acid	869	9.377	[9.231 , 9.522]	2.187	9.190	[7.850 , 10.800]	942	9.490	[9.346 , 9.634]	2.253	9.285	[7.910 , 10.938]
3-Methyl-2-Oxovalerate	869	23.001	[22.676 , 23.326]	4.880	22.470	[19.530 , 25.940]	942	22.865	[22.553 , 23.177]	4.878	22.440	[19.525 , 25.675]

Supplementary Table S2-1 : DateDiff0 male 40’s
(3/7)

DateDiff0 male	40 - 44						45 - 49					
Name	N	Mean	95%CI	SD	Median	Quantile [lower, upper]	N	Mean	95%CI	SD	Median	Quantile [lower, upper]
Acetate	917	33.461	[32.823 , 34.100]	9.850	31.790	[27.340 , 37.270]	665	33.670	[32.546 , 34.793]	14.758	30.750	[27.020 , 36.790]
Acetone	920	5.216	[4.945 , 5.488]	4.198	3.850	[2.610 , 6.165]	673	5.870	[5.473 , 6.267]	5.247	4.200	[2.860 , 6.770]
Alanine	921	374.927	[369.798 , 380.057]	79.321	367.750	[322.500 , 430.160]	673	370.195	[364.124 , 376.266]	80.214	369.130	[317.770 , 422.800]
Arginine	921	52.551	[51.800 , 53.302]	11.615	51.680	[45.150 , 58.420]	673	52.315	[51.470 , 53.160]	11.165	51.740	[45.220 , 59.220]
Asparagine	920	59.356	[58.562 , 60.149]	12.258	58.115	[51.270 , 66.030]	673	58.241	[57.320 , 59.163]	12.173	57.500	[49.910 , 65.420]
Betaine	921	49.053	[48.260 , 49.846]	12.263	47.430	[40.800 , 54.820]	673	48.496	[47.558 , 49.434]	12.389	46.430	[40.100 , 55.690]
Carnitine	916	38.782	[38.236 , 39.328]	8.419	38.475	[32.878 , 43.957]	670	38.363	[37.720 , 39.006]	8.479	37.780	[32.483 , 43.280]
Citrate	921	89.558	[88.377 , 90.739]	18.263	88.260	[76.340 , 100.810]	673	91.980	[90.466 , 93.493]	19.998	90.810	[77.000 , 104.890]
Creatine	921	31.573	[30.707 , 32.438]	13.387	29.200	[22.180 , 38.130]	673	31.162	[30.233 , 32.092]	12.281	28.740	[22.270 , 37.410]
Creatinine	919	67.068	[66.443 , 67.694]	9.666	66.830	[60.660 , 72.955]	673	67.293	[66.509 , 68.077]	10.357	66.800	[60.660 , 72.930]
Cysteine	921	57.842	[56.861 , 58.823]	15.168	56.180	[47.960 , 64.870]	673	60.373	[59.234 , 61.512]	15.051	59.010	[50.420 , 68.060]
Formate	910	10.352	[10.146 , 10.558]	3.167	9.950	[8.335 , 11.625]	667	10.671	[10.435 , 10.907]	3.105	10.200	[8.715 , 12.065]
Glucose	920	5006.300	[4936.934 , 5075.665]	1072.053	4827.540	[4437.205 , 5228.543]	670	5171.741	[5076.928 , 5266.555]	1249.892	4909.495	[4490.490 , 5417.618]
Glutamate	921	60.741	[59.607 , 61.875]	17.537	58.670	[47.830 , 71.490]	673	62.909	[61.537 , 64.282]	18.129	60.560	[49.460 , 73.250]
Glutamine	921	486.910	[483.025 , 490.795]	60.073	487.270	[445.020 , 530.960]	673	484.620	[479.531 , 489.710]	67.247	481.770	[440.000 , 524.330]
Glycerol	921	56.151	[54.481 , 57.820]	25.822	49.320	[37.750 , 68.740]	673	60.076	[58.060 , 62.091]	26.629	54.570	[40.860 , 73.650]
Glycine	921	201.334	[198.534 , 204.133]	43.289	194.900	[171.470 , 223.460]	673	199.153	[195.978 , 202.327]	41.943	192.810	[169.010 , 220.740]
Histidine	920	88.386	[87.692 , 89.080]	10.725	87.910	[81.943 , 94.742]	673	88.026	[87.204 , 88.848]	10.861	87.650	[81.080 , 94.560]
Isoleucine	921	66.703	[65.638 , 67.767]	16.459	64.210	[56.380 , 73.760]	673	66.547	[65.232 , 67.862]	17.377	62.970	[55.230 , 73.960]
Lactate	921	2567.153	[2522.550 , 2611.755]	689.709	2519.980	[2066.760 , 2944.760]	673	2655.692	[2603.837 , 2707.546]	685.110	2548.770	[2178.710 , 3062.910]
Leucine	921	128.628	[127.022 , 130.234]	24.835	125.850	[112.030 , 141.210]	673	128.814	[126.928 , 130.700]	24.923	125.840	[112.990 , 140.820]
Lysine	921	136.597	[135.281 , 137.913]	20.352	135.160	[122.730 , 148.400]	673	137.146	[135.591 , 138.700]	20.541	136.570	[123.530 , 149.200]
Methionine	921	29.265	[29.012 , 29.518]	3.916	28.880	[26.740 , 31.430]	673	29.070	[28.764 , 29.376]	4.041	28.630	[26.590 , 31.190]
N,N-Dimethylglycine	916	2.616	[2.553 , 2.679]	0.969	2.485	[1.968 , 3.070]	671	2.549	[2.482 , 2.616]	0.883	2.430	[1.930 , 2.990]
Ornithine	921	72.221	[71.218 , 73.225]	15.517	71.100	[61.180 , 81.950]	673	74.144	[72.968 , 75.319]	15.534	73.640	[63.060 , 83.910]
Phenylalanine	921	61.015	[60.350 , 61.680]	10.286	59.730	[54.240 , 66.240]	673	61.279	[60.525 , 62.034]	9.967	60.130	[54.430 , 66.370]
Proline	921	185.587	[182.690 , 188.485]	44.803	177.140	[155.910 , 209.060]	673	184.331	[180.836 , 187.825]	46.171	175.740	[153.170 , 202.650]
Pyruvate	921	87.281	[85.170 , 89.392]	32.650	85.740	[62.780 , 108.300]	673	94.154	[91.752 , 96.556]	31.735	93.540	[72.340 , 115.170]
Serine	919	109.579	[108.605 , 110.553]	15.045	108.390	[99.635 , 118.375]	673	110.761	[109.590 , 111.931]	15.468	109.990	[99.950 , 120.860]
Succinate	921	7.412	[7.307 , 7.516]	1.617	7.210	[6.280 , 8.280]	673	7.772	[7.635 , 7.909]	1.806	7.560	[6.580 , 8.720]
Threonine	921	186.009	[183.161 , 188.858]	44.047	184.930	[157.520 , 213.920]	673	188.744	[185.513 , 191.976]	42.696	187.100	[159.100 , 216.310]
Tryptophan	920	56.650	[56.010 , 57.291]	9.897	55.725	[50.362 , 61.942]	673	56.778	[55.982 , 57.574]	10.516	56.160	[50.480 , 62.330]
Tyrosine	921	67.637	[66.767 , 68.507]	13.448	65.850	[58.080 , 74.740]	673	69.113	[68.027 , 70.200]	14.356	67.970	[58.490 , 76.870]
Uridine	895	3.860	[3.801 , 3.919]	0.900	3.800	[3.225 , 4.410]	665	3.973	[3.900 , 4.045]	0.953	3.910	[3.280 , 4.590]
Valine	921	226.028	[223.542 , 228.515]	38.447	222.640	[199.530 , 246.470]	673	224.534	[221.428 , 227.640]	41.034	220.540	[197.180 , 245.900]
2-Aminobutyrate	921	18.625	[18.416 , 18.834]	3.232	18.350	[16.350 , 20.290]	673	18.540	[18.280 , 18.800]	3.437	18.150	[16.300 , 20.430]
2-Hydroxybutyrate	921	35.605	[34.720 , 36.489]	13.676	33.320	[25.910 , 42.390]	673	37.764	[36.534 , 38.995]	16.256	34.360	[27.200 , 44.550]
2-Oxoisocaproate	921	30.906	[30.453 , 31.359]	7.002	30.400	[26.030 , 34.860]	673	31.175	[30.655 , 31.694]	6.867	30.830	[26.340 , 35.200]
3-Hydroxybutyrate	920	60.220	[56.133 , 64.308]	63.173	40.420	[30.768 , 62.040]	673	70.419	[64.443 , 76.395]	78.960	42.500	[31.790 , 70.010]
3-Hydroxyisobutyrate	921	11.924	[11.682 , 12.165]	3.732	11.430	[9.320 , 13.710]	673	12.154	[11.852 , 12.456]	3.988	11.540	[9.510 , 13.900]
3-Methyl-2-Oxobutyric Acid	921	9.736	[9.584 , 9.887]	2.341	9.600	[7.990 , 11.110]	673	9.896	[9.728 , 10.064]	2.221	9.700	[8.310 , 11.360]
3-Methyl-2-Oxovalerate	921	22.800	[22.486 , 23.115]	4.867	22.450	[19.250 , 25.780]	673	23.055	[22.679 , 23.430]	4.958	22.640	[19.620 , 26.050]

Supplementary Table S2-1 : DateDiff0 male 50’s
(4/7)

DateDiff0 male	50 - 54						55 - 59					
Name	N	Mean	95%CI	SD	Median	Quantile [lower, upper]	N	Mean	95%CI	SD	Median	Quantile [lower, upper]
Acetate	746	36.675	[32.440 , 40.910]	58.926	31.505	[27.720 , 36.818]	1095	36.21	[34.303 , 38.118]	32.167	32.080	[27.900 , 38.040]
Acetone	753	6.010	[5.617 , 6.404]	5.501	4.300	[2.780 , 7.100]	1104	6.095	[5.765 , 6.424]	5.580	4.390	[2.930 , 7.045]
Alanine	753	371.723	[365.936 , 377.510]	80.889	367.650	[316.820 , 421.750]	1104	368.392	[363.635 , 373.150]	80.556	366.695	[314.232 , 420.792]
Arginine	753	53.673	[52.770 , 54.575]	12.611	52.800	[45.460 , 60.570]	1104	54.093	[53.452 , 54.734]	10.856	53.975	[47.212 , 60.752]
Asparagine	753	58.416	[57.562 , 59.271]	11.946	57.530	[50.100 , 65.550]	1104	58.362	[57.698 , 59.026]	11.241	57.730	[51.333 , 64.738]
Betaine	753	50.684	[49.632 , 51.735]	14.703	48.320	[40.900 , 57.150]	1104	52.477	[51.623 , 53.332]	14.471	50.760	[43.430 , 59.165]
Carnitine	749	38.684	[38.033 , 39.334]	9.069	37.580	[32.570 , 44.080]	1104	39.234	[38.727 , 39.741]	8.589	38.645	[33.295 , 44.655]
Citrate	753	92.426	[91.026 , 93.825]	19.559	90.120	[79.550 , 104.030]	1104	93.219	[92.042 , 94.395]	19.922	90.835	[79.537 , 104.360]
Creatine	753	31.107	[30.181 , 32.033]	12.943	27.980	[21.170 , 38.560]	1104	33.009	[32.206 , 33.813]	13.602	30.190	[22.568 , 40.530]
Creatinine	753	66.895	[66.111 , 67.678]	10.954	65.500	[59.420 , 73.260]	1104	66.831	[66.135 , 67.527]	11.785	65.610	[59.308 , 72.785]
Cysteine	753	61.915	[60.785 , 63.045]	15.799	59.010	[51.280 , 70.800]	1104	62.614	[61.734 , 63.493]	14.891	60.495	[52.528 , 71.100]
Formate	742	11.305	[11.038 , 11.572]	3.703	10.660	[8.912 , 12.848]	1087	11.253	[11.025 , 11.482]	3.838	10.580	[8.930 , 12.910]
Glucose	752	5371.064	[5257.958 , 5484.169]	1579.950	5044.705	[4607.150 , 5644.300]	1102	5451.282	[5369.827 , 5532.737]	1378.109	5073.420	[4684.647 , 5778.948]
Glutamate	752	63.707	[62.325 , 65.088]	19.300	61.475	[49.693 , 74.390]	1104	63.163	[62.144 , 64.181]	17.249	61.600	[50.480 , 72.878]
Glutamine	753	480.762	[475.975 , 485.549]	66.911	480.880	[440.460 , 525.110]	1104	487.083	[483.088 , 491.079]	67.661	488.620	[445.052 , 531.618]
Glycerol	753	62.068	[59.927 , 64.209]	29.926	54.940	[42.170 , 72.990]	1104	61.331	[59.705 , 62.958]	27.538	55.230	[42.250 , 74.235]
Glycine	753	196.763	[193.935 , 199.591]	39.531	190.410	[170.250 , 218.350]	1104	197.318	[194.949 , 199.688]	40.128	190.285	[169.780 , 217.350]
Histidine	753	87.717	[86.935 , 88.499]	10.929	86.900	[80.230 , 93.920]	1103	87.378	[86.714 , 88.041]	11.225	86.840	[80.560 , 93.910]
Isoleucine	753	65.487	[64.298 , 66.676]	16.624	63.350	[55.410 , 72.520]	1104	65.566	[64.667 , 66.465]	15.224	63.570	[55.130 , 73.065]
Lactate	753	2664.209	[2617.689 , 2710.728]	650.257	2614.620	[2192.000 , 3065.990]	1104	2600.19	[2561.949 , 2638.431]	647.571	2525.190	[2138.645 , 3001.435]
Leucine	753	128.286	[126.403 , 130.170]	26.334	124.990	[112.920 , 139.020]	1104	127.455	[126.086 , 128.824]	23.177	124.960	[112.340 , 139.485]
Lysine	753	136.697	[135.169 , 138.225]	21.361	135.520	[122.600 , 149.700]	1104	138.99	[137.762 , 140.219]	20.803	137.425	[125.900 , 150.290]
Methionine	753	28.854	[28.543 , 29.165]	4.345	28.560	[26.450 , 30.790]	1104	29.021	[28.799 , 29.243]	3.763	28.875	[26.778 , 30.877]
N,N-Dimethylglycine	748	2.644	[2.571 , 2.717]	1.017	2.510	[1.970 , 3.150]	1094	2.699	[2.637 , 2.762]	1.049	2.540	[2.000 , 3.190]
Ornithine	753	74.193	[73.100 , 75.286]	15.277	72.720	[63.170 , 84.020]	1104	74.571	[73.690 , 75.452]	14.921	73.955	[64.390 , 83.383]
Phenylalanine	753	62.354	[61.562 , 63.147]	11.080	60.650	[55.390 , 66.870]	1104	62.984	[62.405 , 63.563]	9.799	62.090	[56.427 , 67.757]
Proline	753	180.475	[177.342 , 183.608]	43.793	173.100	[151.870 , 199.290]	1104	178.256	[175.666 , 180.845]	43.852	170.720	[150.785 , 196.393]
Pyruvate	753	94.998	[92.817 , 97.179]	30.487	94.400	[73.700 , 114.920]	1104	91.248	[89.492 , 93.005]	29.751	90.815	[71.842 , 110.267]
Serine	752	111.929	[110.728 , 113.130]	16.775	110.955	[100.990 , 122.998]	1103	111.964	[110.993 , 112.935]	16.433	110.990	[101.375 , 121.685]
Succinate	753	7.802	[7.673 , 7.931]	1.799	7.680	[6.530 , 8.800]	1104	7.845	[7.743 , 7.947]	1.730	7.730	[6.707 , 8.820]
Threonine	753	187.217	[184.137 , 190.296]	43.050	184.720	[158.360 , 213.690]	1104	184.495	[181.927 , 187.064]	43.494	181.540	[156.072 , 209.573]
Tryptophan	753	56.152	[55.417 , 56.886]	10.268	55.250	[49.090 , 62.220]	1104	56.339	[55.738 , 56.939]	10.167	55.820	[50.017 , 61.975]
Tyrosine	753	69.532	[68.525 , 70.540]	14.083	67.690	[59.520 , 76.880]	1104	71.528	[70.693 , 72.362]	14.130	69.730	[61.797 , 79.545]
Uridine	736	4.072	[4.004 , 4.141]	0.948	4.080	[3.400 , 4.680]	1084	4.053	[3.995 , 4.111]	0.971	4.010	[3.367 , 4.680]
Valine	753	222.742	[219.826 , 225.659]	40.769	219.510	[196.430 , 244.180]	1104	223.203	[220.904 , 225.501]	38.921	220.210	[197.625 , 245.752]
2-Aminobutyrate	753	18.349	[18.108 , 18.590]	3.370	18.080	[16.160 , 20.110]	1104	18.401	[18.211 , 18.591]	3.217	18.240	[16.238 , 20.262]
2-Hydroxybutyrate	753	39.250	[37.757 , 40.744]	20.876	35.850	[27.830 , 46.160]	1104	38.232	[37.339 , 39.124]	15.108	35.290	[27.700 , 45.378]
2-Oxoisocaproate	753	31.139	[30.619 , 31.660]	7.279	30.450	[26.100 , 35.710]	1104	30.672	[30.271 , 31.074]	6.792	30.250	[25.758 , 34.752]
3-Hydroxybutyrate	753	73.479	[67.939 , 79.019]	77.443	44.660	[33.130 , 78.220]	1104	70.219	[65.955 , 74.483]	72.206	43.845	[31.822 , 75.575]
3-Hydroxyisobutyrate	753	12.332	[12.019 , 12.644]	4.372	11.530	[9.610 , 14.330]	1104	12.379	[12.162 , 12.597]	3.681	11.870	[9.838 , 14.332]
3-Methyl-2-Oxobutyric Acid	753	9.896	[9.731 , 10.061]	2.303	9.720	[8.300 , 11.440]	1104	9.668	[9.538 , 9.799]	2.209	9.530	[8.100 , 11.080]
3-Methyl-2-Oxovalerate	753	22.987	[22.628 , 23.347]	5.026	22.450	[19.510 , 26.110]	1104	22.607	[22.329 , 22.885]	4.707	22.195	[19.250 , 25.575]

Supplementary Table S2-1 : DateDiff0 male 60’s
(5/7)

DateDiff0 male	60 - 64						65 - 69					
Name	N	Mean	95%CI	SD	Median	Quantile [lower, upper]	N	Mean	95%CI	SD	Median	Quantile [lower, upper]
Acetate	1642	36.258	[35.467 , 37.048]	16.337	33.510	[29.320 , 40.110]	2685	36.829	[36.301 , 37.356]	13.951	34.130	[29.710 , 40.580]
Acetone	1649	6.454	[6.180 , 6.728]	5.673	4.670	[3.070 , 7.490]	2689	6.444	[6.230 , 6.658]	5.662	4.730	[3.010 , 7.680]
Alanine	1650	369.882	[366.163 , 373.602]	77.024	364.315	[315.812 , 420.752]	2691	367.283	[364.477 , 370.090]	74.252	362.840	[313.660 , 416.025]
Arginine	1650	54.158	[53.613 , 54.702]	11.274	53.760	[46.662 , 60.720]	2691	54.104	[53.698 , 54.509]	10.737	53.500	[46.765 , 60.715]
Asparagine	1650	60.116	[59.536 , 60.697]	12.020	58.920	[52.267 , 66.282]	2691	59.972	[59.523 , 60.420]	11.867	58.960	[52.375 , 66.310]
Betaine	1650	53.966	[53.271 , 54.660]	14.385	52.175	[43.900 , 61.800]	2690	54.271	[53.740 , 54.801]	14.031	52.500	[44.730 , 61.698]
Carnitine	1645	40.334	[39.911 , 40.757]	8.752	39.980	[34.370 , 45.580]	2688	39.848	[39.517 , 40.178]	8.733	39.280	[34.080 , 44.890]
Citrate	1650	98.67	[97.689 , 99.651]	20.322	96.630	[85.105 , 111.322]	2691	101.808	[101.033 , 102.584]	20.521	100.130	[87.540 , 114.160]
Creatine	1650	33.257	[32.562 , 33.952]	14.388	29.950	[22.823 , 40.820]	2691	33.678	[33.153 , 34.203]	13.889	31.050	[23.235 , 41.075]
Creatinine	1648	68.835	[68.191 , 69.479]	13.330	67.515	[60.520 , 75.062]	2691	70.030	[69.384 , 70.677]	17.104	67.890	[61.150 , 76.570]
Cysteine	1650	65.266	[64.535 , 65.997]	15.144	63.480	[55.135 , 73.438]	2691	66.971	[66.411 , 67.531]	14.815	65.240	[56.835 , 75.190]
Formate	1650	11.734	[11.553 , 11.914]	3.735	11.000	[9.453 , 13.408]	2691	11.933	[11.792 , 12.074]	3.728	11.340	[9.625 , 13.565]
Glucose	1648	5556.627	[5489.767 , 5623.487]	1383.813	5230.925	[4797.260 , 5825.925]	2689	5538.297	[5487.918 , 5588.676]	1332.301	5219.010	[4770.200 , 5852.340]
Glutamate	1650	61.53	[60.756 , 62.303]	16.022	59.750	[49.778 , 71.505]	2691	60.169	[59.586 , 60.753]	15.427	58.380	[48.985 , 69.570]
Glutamine	1650	493.035	[489.957 , 496.113]	63.739	491.775	[450.937 , 533.912]	2691	494.740	[492.279 , 497.200]	65.095	495.050	[450.775 , 535.775]
Glycerol	1650	64.64	[63.298 , 65.982]	27.796	58.660	[43.197 , 81.005]	2691	66.591	[65.535 , 67.646]	27.914	60.710	[46.445 , 81.860]
Glycine	1650	197.216	[195.332 , 199.101]	39.033	191.470	[169.705 , 216.572]	2691	194.561	[193.051 , 196.072]	39.967	188.500	[168.190 , 213.960]
Histidine	1649	87.483	[86.954 , 88.011]	10.938	86.920	[80.600 , 93.440]	2690	86.691	[86.288 , 87.094]	10.654	86.080	[79.410 , 92.638]
Isoleucine	1650	66.395	[65.682 , 67.109]	14.779	64.325	[56.353 , 74.363]	2691	66.654	[66.076 , 67.233]	15.298	64.650	[56.545 , 74.065]
Lactate	1650	2540.873	[2509.614 , 2572.132]	647.367	2464.335	[2071.728 , 2902.660]	2691	2524.743	[2500.393 , 2549.094]	644.209	2434.740	[2066.375 , 2890.315]
Leucine	1650	128.419	[127.333 , 129.505]	22.489	126.060	[113.435 , 140.910]	2691	128.036	[127.173 , 128.900]	22.854	125.640	[113.255 , 140.950]
Lysine	1650	140.965	[139.968 , 141.961]	20.642	139.960	[127.385 , 152.962]	2691	142.007	[141.235 , 142.779]	20.420	140.390	[128.460 , 154.715]
Methionine	1650	28.898	[28.711 , 29.085]	3.873	28.710	[26.582 , 30.958]	2691	28.747	[28.605 , 28.888]	3.750	28.500	[26.360 , 30.660]
N,N-Dimethylglycine	1617	2.755	[2.699 , 2.810]	1.144	2.610	[2.060 , 3.200]	2653	2.715	[2.675 , 2.754]	1.034	2.550	[2.040 , 3.170]
Ornithine	1650	74.445	[73.703 , 75.187]	15.363	73.040	[63.772 , 83.468]	2691	75.223	[74.655 , 75.791]	15.022	73.700	[64.805 , 84.200]
Phenylalanine	1650	64.889	[64.418 , 65.360]	9.755	63.600	[58.453 , 70.225]	2691	65.665	[65.272 , 66.058]	10.390	64.100	[58.985 , 70.600]
Proline	1650	179.106	[177.120 , 181.092]	41.135	172.285	[151.795 , 198.440]	2690	178.267	[176.718 , 179.816]	40.963	171.840	[151.062 , 196.468]
Pyruvate	1650	88.638	[87.189 , 90.087]	30.015	88.575	[68.310 , 107.638]	2691	88.908	[87.808 , 90.008]	29.103	87.360	[70.230 , 105.605]
Serine	1646	114.318	[113.593 , 115.042]	14.986	112.730	[104.538 , 122.690]	2687	113.276	[112.717 , 113.836]	14.798	111.850	[103.010 , 122.065]
Succinate	1650	7.997	[7.910 , 8.083]	1.800	7.835	[6.725 , 9.008]	2691	8.210	[8.141 , 8.279]	1.814	8.010	[7.000 , 9.230]
Threonine	1650	188.881	[186.783 , 190.980]	43.466	189.290	[160.810 , 215.858]	2691	185.270	[183.661 , 186.879]	42.569	184.770	[158.005 , 212.740]
Tryptophan	1650	56.079	[55.604 , 56.554]	9.832	55.515	[49.935 , 61.920]	2691	55.620	[55.269 , 55.971]	9.275	55.050	[49.345 , 61.180]
Tyrosine	1649	73.15	[72.462 , 73.838]	14.248	71.590	[63.190 , 80.820]	2691	73.446	[72.893 , 73.999]	14.630	71.400	[63.380 , 81.705]
Uridine	1605	4.045	[4.000 , 4.091]	0.929	4.010	[3.380 , 4.640]	2627	4.036	[4.000 , 4.072]	0.943	3.990	[3.370 , 4.660]
Valine	1650	224.15	[222.399 , 225.901]	36.263	221.250	[198.957 , 245.422]	2691	225.550	[224.144 , 226.955]	37.188	221.410	[200.610 , 246.255]
2-Aminobutyrate	1650	18.65	[18.502 , 18.798]	3.061	18.370	[16.572 , 20.488]	2691	18.798	[18.679 , 18.916]	3.128	18.460	[16.695 , 20.580]
2-Hydroxybutyrate	1650	39.094	[38.398 , 39.790]	14.415	37.005	[28.573 , 46.455]	2691	39.703	[39.150 , 40.256]	14.635	37.160	[29.600 , 47.340]
2-Oxoisocaproate	1650	30.598	[30.282 , 30.915]	6.551	30.150	[25.985 , 34.702]	2691	30.409	[30.168 , 30.649]	6.369	30.010	[26.065 , 34.345]
3-Hydroxybutyrate	1650	79.653	[75.694 , 83.613]	82.003	47.960	[33.802 , 92.353]	2691	84.190	[80.845 , 87.535]	88.485	50.410	[34.095 , 99.815]
3-Hydroxyisobutyrate	1650	12.806	[12.626 , 12.985]	3.718	12.380	[10.180 , 14.920]	2691	13.021	[12.880 , 13.162]	3.737	12.430	[10.530 , 14.890]
3-Methyl-2-Oxobutyric Acid	1650	9.675	[9.572 , 9.777]	2.120	9.575	[8.213 , 11.030]	2691	9.662	[9.585 , 9.739]	2.037	9.580	[8.250 , 10.915]
3-Methyl-2-Oxovalerate	1650	22.631	[22.411 , 22.852]	4.562	22.215	[19.350 , 25.497]	2691	22.467	[22.299 , 22.635]	4.446	22.150	[19.400 , 25.095]

Supplementary Table S2-1 : DateDiff0 male 70's
(6/7)

DateDiff0 male	70 - 74						75 - 79					
Name	N	Mean	95%CI	SD	Median	Quantile [lower, upper]	N	Mean	95%CI	SD	Median	Quantile [lower, upper]
Acetate	2459	37.238	[36.735 , 37.742]	12.738	34.480	[29.920 , 40.980]	254	40.349	[38.826 , 41.871]	12.318	37.325	[32.098 , 45.562]
Acetone	2464	6.401	[6.181 , 6.621]	5.570	4.580	[2.950 , 7.802]	254	7.526	[6.736 , 8.315]	6.391	5.090	[3.290 , 9.600]
Alanine	2465	366.995	[364.006 , 369.984]	75.674	361.300	[312.560 , 417.380]	254	363.659	[354.472 , 372.846]	74.345	361.185	[308.788 , 410.228]
Arginine	2465	54.601	[54.165 , 55.037]	11.041	53.870	[47.490 , 61.270]	254	55.719	[54.265 , 57.173]	11.765	55.725	[47.972 , 63.502]
Asparagine	2465	60.543	[60.058 , 61.028]	12.283	59.080	[52.510 , 67.180]	254	60.23	[58.536 , 61.923]	13.706	58.475	[50.865 , 68.507]
Betaine	2464	56.321	[55.735 , 56.908]	14.845	54.075	[46.228 , 64.080]	254	55.628	[53.751 , 57.504]	15.187	53.210	[45.752 , 63.000]
Carnitine	2463	39.195	[38.858 , 39.532]	8.529	38.680	[33.320 , 44.675]	254	40.891	[39.913 , 41.868]	7.911	41.490	[35.775 , 45.838]
Citrate	2465	106.212	[105.381 , 107.043]	21.039	104.270	[91.700 , 118.500]	254	108.809	[106.182 , 111.436]	21.261	107.960	[93.622 , 121.250]
Creatine	2465	32.513	[31.991 , 33.035]	13.212	29.520	[22.400 , 40.820]	254	31.305	[29.576 , 33.034]	13.993	27.970	[20.678 , 36.892]
Creatinine	2461	71.233	[70.651 , 71.816]	14.730	69.150	[61.780 , 78.100]	254	73.8	[71.941 , 75.658]	15.039	72.055	[64.193 , 80.485]
Cysteine	2464	68.241	[67.623 , 68.858]	15.637	65.685	[57.547 , 76.760]	254	65.572	[63.862 , 67.283]	13.843	64.530	[55.910 , 74.908]
Formate	2463	12.631	[12.454 , 12.808]	4.478	11.770	[9.970 , 14.170]	254	12.512	[11.992 , 13.031]	4.207	11.750	[9.860 , 14.332]
Glucose	2462	5552.293	[5501.941 , 5602.645]	1274.081	5260.935	[4800.612 , 5947.520]	254	5693.861	[5499.977 , 5887.744]	1569.014	5340.800	[4843.682 , 5996.587]
Glutamate	2465	58.038	[57.448 , 58.627]	14.926	56.300	[47.200 , 66.580]	254	53.908	[52.154 , 55.662]	14.193	52.095	[43.333 , 63.665]
Glutamine	2465	499.548	[497.003 , 502.094]	64.457	496.480	[457.300 , 543.120]	254	513.336	[504.947 , 521.726]	67.893	512.985	[472.240 , 555.845]
Glycerol	2465	69.959	[68.820 , 71.098]	28.829	64.690	[48.070 , 86.610]	254	74.745	[70.779 , 78.710]	32.093	70.245	[50.342 , 91.770]
Glycine	2465	193.192	[191.724 , 194.660]	37.162	187.820	[168.380 , 212.070]	254	196.563	[192.135 , 200.991]	35.833	194.600	[173.472 , 216.423]
Histidine	2464	86.163	[85.737 , 86.590]	10.791	85.250	[79.068 , 92.463]	253	86.681	[85.151 , 88.212]	12.360	86.290	[79.340 , 92.950]
Isoleucine	2465	66.089	[65.522 , 66.655]	14.348	64.210	[56.270 , 73.630]	254	68.084	[66.166 , 70.002]	15.523	65.815	[58.438 , 75.075]
Lactate	2465	2469.845	[2445.913 , 2493.777]	605.943	2399.430	[2033.310 , 2840.090]	254	2231.89	[2164.116 , 2299.665]	548.469	2205.330	[1856.863 , 2487.650]
Leucine	2465	126.315	[125.414 , 127.216]	22.814	124.200	[111.220 , 139.120]	254	125.152	[122.573 , 127.730]	20.868	122.645	[112.602 , 136.545]
Lysine	2465	142.396	[141.556 , 143.236]	21.271	141.060	[128.430 , 154.730]	254	141.218	[138.372 , 144.064]	23.033	138.750	[125.488 , 155.380]
Methionine	2465	28.601	[28.455 , 28.748]	3.707	28.370	[26.120 , 30.630]	254	28.596	[28.085 , 29.107]	4.134	28.115	[26.160 , 30.830]
N,N-Dimethylglycine	2436	2.764	[2.721 , 2.806]	1.069	2.600	[2.060 , 3.240]	251	2.713	[2.596 , 2.830]	0.941	2.590	[2.125 , 3.240]
Ornithine	2465	75.929	[75.339 , 76.518]	14.930	74.760	[65.670 , 84.510]	254	72.803	[71.021 , 74.585]	14.424	69.690	[62.073 , 80.892]
Phenylalanine	2465	66.467	[66.059 , 66.875]	10.329	65.170	[59.480 , 71.760]	254	67.23	[65.878 , 68.583]	10.945	65.400	[59.635 , 72.083]
Proline	2465	177.885	[176.271 , 179.499]	40.860	170.170	[150.450 , 198.270]	254	171.838	[167.537 , 176.139]	34.809	167.230	[149.323 , 188.245]
Pyruvate	2465	89.95	[88.913 , 90.987]	26.259	88.720	[73.660 , 104.530]	254	72.146	[68.855 , 75.438]	26.634	70.995	[54.335 , 90.025]
Serine	2462	113.311	[112.741 , 113.880]	14.410	112.090	[103.380 , 122.078]	254	115.806	[113.854 , 117.758]	15.799	113.625	[105.060 , 124.715]
Succinate	2465	8.494	[8.420 , 8.568]	1.870	8.280	[7.250 , 9.530]	254	8.098	[7.860 , 8.335]	1.921	7.910	[6.820 , 9.117]
Threonine	2465	180.742	[179.105 , 182.379]	41.447	180.090	[152.790 , 206.040]	254	182.766	[178.043 , 187.490]	38.225	183.205	[158.162 , 207.787]
Tryptophan	2465	54.554	[54.175 , 54.932]	9.589	54.160	[48.320 , 60.180]	254	53.574	[52.414 , 54.734]	9.386	53.800	[47.135 , 59.433]
Tyrosine	2465	74.388	[73.809 , 74.968]	14.675	72.830	[64.330 , 82.540]	254	72.934	[71.366 , 74.503]	12.690	72.090	[65.155 , 79.770]
Uridine	2416	3.985	[3.949 , 4.020]	0.892	3.950	[3.340 , 4.582]	250	4.045	[3.924 , 4.165]	0.969	3.980	[3.433 , 4.725]
Valine	2465	223.78	[222.319 , 225.240]	36.984	221.070	[197.850 , 245.690]	254	227.152	[222.504 , 231.800]	37.614	223.985	[202.267 , 245.645]
2-Aminobutyrate	2465	18.649	[18.528 , 18.771]	3.066	18.490	[16.530 , 20.410]	254	18.794	[18.415 , 19.172]	3.062	18.535	[16.810 , 20.422]
2-Hydroxybutyrate	2464	39.023	[38.466 , 39.579]	14.085	36.985	[28.747 , 46.940]	254	38.311	[36.528 , 40.095]	14.433	35.880	[27.062 , 46.388]
2-Oxoisocaproate	2465	29.846	[29.598 , 30.095]	6.291	29.610	[25.600 , 33.690]	254	29.326	[28.551 , 30.101]	6.273	28.875	[24.735 , 33.472]
3-Hydroxybutyrate	2464	90.17	[86.566 , 93.774]	91.232	53.920	[34.265 , 113.833]	254	99.34	[88.484 , 110.196]	87.853	63.280	[35.905 , 139.075]
3-Hydroxyisobutyrate	2465	13.137	[12.984 , 13.290]	3.869	12.690	[10.480 , 15.180]	254	12.771	[12.298 , 13.243]	3.821	12.100	[10.220 , 14.590]
3-Methyl-2-Oxobutyric Acid	2465	9.591	[9.509 , 9.673]	2.068	9.420	[8.190 , 10.800]	254	9.324	[9.066 , 9.582]	2.089	9.010	[7.852 , 10.638]
3-Methyl-2-Oxovalerate	2465	22.194	[22.024 , 22.364]	4.310	21.870	[19.340 , 24.720]	254	21.91	[21.384 , 22.436]	4.258	21.330	[18.753 , 24.910]

Supplementary Table S2-1 : DateDiff0 male Over 80's
(7/7)

DateDiff0 male		over 80												
Name	N	Mean	95%CI					SD	Median	Quantile [lower, upper]				
Acetate	63	47.207	[	43.222	,	51.192	]	15.823	42.990	[	35.400	,	56.440	]
Acetone	63	10.218	[	8.215	,	12.221	]	7.955	6.900	[	4.380	,	15.320	]
Alanine	63	358.243	[	338.790	,	377.696	]	77.241	341.970	[	304.455	,	414.835	]
Arginine	63	60.071	[	55.816	,	64.326	]	16.895	59.250	[	50.840	,	68.850	]
Asparagine	63	57.517	[	54.715	,	60.319	]	11.126	56.100	[	49.430	,	64.215	]
Betaine	63	58.286	[	54.734	,	61.837	]	14.100	59.960	[	47.195	,	68.470	]
Carnitine	63	43.923	[	41.370	,	46.476	]	10.137	42.370	[	39.050	,	48.195	]
Citrate	63	119.568	[	114.152	,	124.984	]	21.504	120.750	[	108.025	,	134.785	]
Creatine	63	29.361	[	26.163	,	32.558	]	12.695	26.380	[	20.395	,	37.195	]
Creatinine	63	76.17	[	72.422	,	79.917	]	14.880	74.420	[	67.000	,	81.720	]
Cysteine	63	63.323	[	60.059	,	66.587	]	12.960	62.990	[	54.695	,	72.910	]
Formate	63	13.476	[	12.230	,	14.721	]	4.945	12.760	[	9.795	,	15.295	]
Glucose	63	5358.394	[	5188.222	,	5528.565	]	675.695	5239.730	[	4949.460	,	5590.145	]
Glutamate	63	49.363	[	45.945	,	52.782	]	13.573	45.780	[	38.990	,	55.975	]
Glutamine	63	517.344	[	501.896	,	532.792	]	61.338	510.310	[	480.890	,	560.185	]
Glycerol	63	92.412	[	84.007	,	100.817	]	33.374	88.570	[	72.115	,	113.295	]
Glycine	63	200.683	[	188.344	,	213.022	]	48.993	190.900	[	173.310	,	217.270	]
Histidine	63	87.207	[	84.430	,	89.985	]	11.028	88.430	[	80.810	,	93.365	]
Isoleucine	63	69.26	[	65.937	,	72.583	]	13.193	70.290	[	59.635	,	76.935	]
Lactate	63	2092.501	[	1947.750	,	2237.252	]	574.758	1962.630	[	1707.725	,	2347.010	]
Leucine	63	122.48	[	117.762	,	127.198	]	18.733	119.430	[	111.125	,	137.750	]
Lysine	63	137.929	[	131.410	,	144.448	]	25.885	134.380	[	122.395	,	149.425	]
Methionine	63	27.797	[	26.771	,	28.823	]	4.073	28.000	[	24.470	,	30.275	]
N,N-Dimethylglycine	63	2.603	[	2.407	,	2.800	]	0.781	2.520	[	2.110	,	3.090	]
Ornithine	63	70.148	[	66.599	,	73.697	]	14.093	67.550	[	59.990	,	79.315	]
Phenylalanine	63	66.505	[	64.432	,	68.578	]	8.232	65.830	[	60.420	,	72.540	]
Proline	63	182.017	[	167.230	,	196.804	]	58.714	168.510	[	147.045	,	195.945	]
Pyruvate	63	54.118	[	49.757	,	58.478	]	17.313	56.100	[	40.260	,	67.050	]
Serine	62	121.247	[	116.510	,	125.984	]	18.655	121.005	[	109.982	,	132.462	]
Succinate	63	7.599	[	7.199	,	7.998	]	1.587	7.430	[	6.435	,	8.340	]
Threonine	63	185.922	[	177.032	,	194.811	]	35.297	183.170	[	160.970	,	204.595	]
Tryptophan	63	52.24	[	50.156	,	54.324	]	8.274	52.780	[	46.065	,	57.965	]
Tyrosine	63	74.055	[	70.833	,	77.278	]	12.796	73.560	[	65.565	,	84.305	]
Uridine	63	3.987	[	3.769	,	4.205	]	0.865	3.930	[	3.210	,	4.570	]
Valine	63	225.729	[	217.958	,	233.499	]	30.854	221.090	[	203.205	,	250.275	]
2-Aminobutyrate	63	18.623	[	17.914	,	19.331	]	2.813	18.350	[	16.475	,	20.820	]
2-Hydroxybutyrate	63	40.745	[	36.863	,	44.628	]	15.415	37.840	[	31.085	,	51.930	]
2-Oxoisocaproate	63	29.663	[	27.877	,	31.449	]	7.093	29.670	[	24.360	,	34.685	]
3-Hydroxybutyrate	63	136.492	[	109.807	,	163.177	]	105.957	104.840	[	49.080	,	183.880	]
3-Hydroxyisobutyrate	63	13.099	[	12.058	,	14.139	]	4.131	12.920	[	9.670	,	15.525	]
3-Methyl-2-Oxobutyric Acid	63	9.519	[	8.860	,	10.177	]	2.614	9.150	[	7.615	,	11.380	]
3-Methyl-2-Oxovalerate	63	22.657	[	21.325	,	23.989	]	5.288	22.320	[	18.485	,	25.620	]

Supplementary Table S2-2 : DateDiff1 male 20’s
(1/7)

DateDiff1 male	20 - 24						25 - 29					
Name	N	Mean	95%CI	SD	Median	Quantile [lower, upper]	N	Mean	95%CI	SD	Median	Quantile [lower, upper]
Acetate	38	28.217	[26.802 , 29.631]	4.303	27.670	[25.180 , 31.048]	159	28.076	[27.279 , 28.873]	5.087	27.420	[24.785 , 30.370]
Acetone	38	4.100	[3.104 , 5.095]	3.029	3.420	[2.223 , 4.605]	159	4.621	[3.983 , 5.258]	4.068	3.760	[2.675 , 4.980]
Alanine	38	384.957	[367.666 , 402.248]	52.606	384.600	[341.422 , 421.700]	159	379.814	[370.060 , 389.567]	62.269	382.230	[336.370 , 413.685]
Arginine	38	51.744	[47.611 , 55.876]	12.572	52.655	[48.290 , 60.545]	159	51.382	[49.360 , 53.405]	12.910	51.260	[43.190 , 58.780]
Asparagine	37	60.975	[56.738 , 65.212]	12.708	58.240	[53.020 , 68.540]	159	62.671	[60.412 , 64.929]	14.418	60.740	[53.265 , 69.875]
Betaine	38	48.838	[45.547 , 52.128]	10.010	47.240	[40.852 , 55.145]	159	50.190	[48.305 , 52.075]	12.034	49.160	[41.325 , 55.720]
Carnitine	38	41.635	[38.640 , 44.629]	9.110	41.380	[35.015 , 48.468]	159	41.250	[39.864 , 42.636]	8.848	39.960	[34.190 , 47.005]
Citrate	38	93.152	[86.886 , 99.417]	19.061	91.230	[78.472 , 104.230]	159	90.698	[87.641 , 93.754]	19.515	88.980	[75.865 , 101.655]
Creatine	38	38.851	[33.181 , 44.521]	17.250	33.255	[26.188 , 54.008]	159	36.057	[33.391 , 38.724]	17.023	31.570	[24.530 , 43.255]
Creatinine	38	66.831	[63.720 , 69.942]	9.464	67.145	[62.228 , 71.318]	159	67.973	[66.609 , 69.338]	8.714	68.160	[62.310 , 73.925]
Cysteine	38	47.271	[42.098 , 52.445]	15.739	43.715	[36.390 , 53.385]	159	50.715	[48.655 , 52.775]	13.153	50.180	[43.250 , 57.900]
Formate	38	11.307	[10.522 , 12.091]	2.387	11.045	[9.480 , 12.415]	159	11.014	[10.608 , 11.420]	2.591	11.050	[9.235 , 12.435]
Glucose	38	3721.320	[3519.249 , 3923.391]	614.774	3725.300	[3387.905 , 4056.837]	159	4118.801	[3986.775 , 4250.827]	842.889	4060.580	[3682.535 , 4484.190]
Glutamate	38	59.575	[53.745 , 65.406]	17.739	55.535	[46.595 , 67.852]	159	63.337	[60.490 , 66.183]	18.174	60.380	[50.210 , 73.380]
Glutamine	38	482.567	[465.569 , 499.565]	51.714	474.265	[450.692 , 513.838]	159	492.166	[483.772 , 500.561]	53.591	492.410	[455.955 , 525.620]
Glycerol	38	56.569	[48.140 , 64.998]	25.643	50.060	[40.470 , 63.415]	159	53.004	[49.450 , 56.558]	22.690	48.940	[37.780 , 62.695]
Glycine	38	217.260	[202.986 , 231.534]	43.427	208.115	[189.088 , 236.840]	159	214.464	[208.506 , 220.423]	38.040	214.220	[187.640 , 236.280]
Histidine	37	88.821	[85.367 , 92.275]	10.360	91.210	[80.510 , 96.640]	159	89.905	[88.004 , 91.806]	12.135	89.190	[82.065 , 96.930]
Isoleucine	38	74.334	[69.652 , 79.016]	14.244	74.210	[61.400 , 84.360]	159	73.375	[70.479 , 76.270]	18.483	70.160	[60.175 , 81.000]
Lactate	38	3915.050	[3655.532 , 4174.568]	789.549	3819.330	[3459.458 , 4187.355]	159	3769.360	[3630.127 , 3908.592]	888.898	3693.600	[3157.660 , 4229.700]
Leucine	38	140.896	[133.694 , 148.098]	21.910	140.225	[127.242 , 154.730]	159	137.865	[134.034 , 141.696]	24.456	133.570	[121.280 , 151.040]
Lysine	38	139.411	[133.312 , 145.509]	18.554	136.845	[126.555 , 151.440]	159	134.314	[130.815 , 137.813]	22.338	132.750	[118.270 , 147.150]
Methionine	38	27.732	[26.653 , 28.811]	3.284	27.605	[25.480 , 29.008]	159	27.728	[27.037 , 28.419]	4.413	26.800	[24.970 , 29.445]
N,N-Dimethylglycine	38	2.977	[2.628 , 3.325]	1.060	2.675	[2.277 , 3.265]	158	2.797	[2.635 , 2.960]	1.036	2.660	[2.072 , 3.317]
Ornithine	38	73.327	[67.521 , 79.132]	17.661	72.500	[65.197 , 81.250]	159	72.486	[70.080 , 74.891]	15.355	71.460	[61.075 , 81.700]
Phenylalanine	38	63.423	[59.701 , 67.145]	11.325	59.565	[56.062 , 67.688]	159	62.865	[61.233 , 64.498]	10.422	61.430	[56.420 , 67.720]
Proline	38	211.456	[196.038 , 226.873]	46.906	201.255	[179.565 , 241.817]	159	204.091	[196.946 , 211.236]	45.616	200.160	[169.320 , 234.985]
Pyruvate	38	42.125	[36.928 , 47.322]	15.812	37.775	[33.868 , 45.645]	159	43.922	[39.778 , 48.066]	26.455	38.780	[32.295 , 49.160]
Serine	38	109.736	[104.578 , 114.893]	15.692	107.670	[99.540 , 118.667]	159	111.079	[108.732 , 113.426]	14.985	110.390	[102.450 , 118.850]
Succinate	38	8.584	[8.092 , 9.075]	1.495	8.470	[7.852 , 9.067]	159	8.633	[8.340 , 8.926]	1.871	8.360	[7.385 , 9.385]
Threonine	38	209.978	[197.976 , 221.980]	36.515	199.875	[181.875 , 232.065]	159	206.853	[199.185 , 214.520]	48.951	202.530	[172.690 , 239.035]
Tryptophan	38	62.376	[57.451 , 67.300]	14.982	60.505	[55.798 , 65.300]	159	59.404	[57.861 , 60.947]	9.852	59.410	[52.860 , 65.260]
Tyrosine	38	67.529	[63.510 , 71.548]	12.228	64.785	[62.450 , 72.298]	159	67.149	[65.129 , 69.169]	12.896	66.540	[58.395 , 74.015]
Uridine	37	4.228	[3.887 , 4.569]	1.023	4.280	[3.520 , 4.950]	156	3.887	[3.739 , 4.035]	0.936	3.855	[3.030 , 4.472]
Valine	38	243.744	[231.017 , 256.471]	38.720	233.835	[216.782 , 266.242]	159	237.540	[231.199 , 243.881]	40.483	233.090	[205.790 , 259.225]
2-Aminobutyrate	38	20.335	[19.388 , 21.282]	2.881	20.085	[18.317 , 21.650]	159	19.494	[19.007 , 19.982]	3.111	19.280	[17.160 , 21.340]
2-Hydroxybutyrate	38	35.397	[31.259 , 39.534]	12.587	31.765	[25.870 , 42.870]	159	33.310	[31.265 , 35.355]	13.055	29.740	[24.225 , 40.440]
2-Oxoisocaproate	38	29.373	[27.364 , 31.381]	6.111	28.940	[24.820 , 33.802]	159	29.447	[28.402 , 30.492]	6.670	28.540	[25.145 , 33.945]
3-Hydroxybutyrate	38	56.577	[36.207 , 76.946]	61.972	34.155	[26.298 , 52.228]	159	56.899	[46.799 , 66.999]	64.481	35.840	[28.465 , 48.185]
3-Hydroxyisobutyrate	38	12.408	[11.455 , 13.360]	2.899	12.445	[10.345 , 13.835]	159	11.737	[11.120 , 12.354]	3.941	11.190	[8.985 , 13.365]
3-Methyl-2-Oxobutyric Acid	38	7.489	[7.011 , 7.968]	1.456	7.060	[6.365 , 8.852]	159	7.297	[7.009 , 7.585]	1.839	7.240	[6.030 , 8.380]
3-Methyl-2-Oxovalerate	38	23.412	[22.124 , 24.700]	3.919	23.380	[20.205 , 26.040]	159	23.569	[22.842 , 24.297]	4.645	23.070	[20.135 , 25.940]

Supplementary Table S2-2 : DateDiff1 male 30’s
(2/7)

DateDiff1 male	30 - 34						35 - 39					
Name	N	Mean	95%CI	SD	Median	Quantile [lower, upper]	N	Mean	95%CI	SD	Median	Quantile [lower, upper]
Acetate	284	29.188	[28.267 , 30.109]	7.882	27.565	[24.675 , 31.938]	318	29.934	[28.102 , 31.765]	16.598	27.915	[24.798 , 31.977]
Acetone	285	5.234	[4.613 , 5.855]	5.325	3.860	[2.660 , 5.870]	319	4.922	[4.487 , 5.357]	3.948	3.780	[2.640 , 5.905]
Alanine	285	374.024	[366.702 , 381.346]	62.796	381.240	[333.700 , 414.550]	319	370.599	[363.336 , 377.863]	65.940	369.770	[324.465 , 412.985]
Arginine	285	51.792	[50.455 , 53.129]	11.464	51.540	[43.890 , 58.610]	319	54.119	[52.768 , 55.471]	12.269	53.380	[46.610 , 61.560]
Asparagine	285	61.947	[60.502 , 63.393]	12.397	61.150	[53.170 , 69.530]	319	59.755	[58.394 , 61.116]	12.358	57.670	[51.355 , 66.310]
Betaine	285	51.767	[50.254 , 53.279]	12.970	50.410	[42.410 , 59.560]	319	50.161	[48.899 , 51.422]	11.452	49.190	[42.335 , 57.230]
Carnitine	284	40.939	[39.954 , 41.924]	8.435	40.705	[35.638 , 46.513]	319	41.248	[40.298 , 42.198]	8.621	40.600	[35.185 , 46.590]
Citrate	285	88.631	[86.402 , 90.861]	19.124	87.470	[74.960 , 99.120]	319	89.782	[87.875 , 91.689]	17.307	89.850	[76.475 , 100.080]
Creatine	285	34.016	[32.235 , 35.797]	15.276	30.860	[23.790 , 40.520]	319	34.367	[32.824 , 35.911]	14.009	30.790	[23.665 , 42.460]
Creatinine	285	67.967	[66.898 , 69.037]	9.173	66.890	[61.810 , 73.760]	319	67.548	[66.566 , 68.530]	8.916	67.300	[62.000 , 72.710]
Cysteine	285	51.470	[49.896 , 53.045]	13.501	51.290	[42.720 , 58.690]	319	52.512	[50.961 , 54.064]	14.085	51.600	[43.580 , 60.675]
Formate	285	10.531	[10.266 , 10.797]	2.279	10.570	[8.760 , 12.170]	319	11.147	[10.840 , 11.454]	2.791	10.920	[9.175 , 12.765]
Glucose	285	4161.643	[4067.963 , 4255.324]	803.466	4033.140	[3721.400 , 4491.960]	319	4274.478	[4143.791 , 4405.165]	1186.379	4083.920	[3759.040 , 4501.155]
Glutamate	285	63.910	[61.501 , 66.320]	20.662	60.450	[48.270 , 75.770]	319	65.441	[63.228 , 67.654]	20.089	61.500	[51.215 , 76.420]
Glutamine	285	496.652	[489.761 , 503.543]	59.104	497.000	[463.870 , 533.250]	319	493.114	[486.561 , 499.667]	59.488	489.540	[452.325 , 531.070]
Glycerol	285	54.056	[51.314 , 56.799]	23.519	47.870	[38.300 , 67.240]	319	56.397	[53.603 , 59.192]	25.366	50.400	[38.960 , 68.195]
Glycine	285	218.016	[212.855 , 223.177]	44.263	210.860	[186.670 , 240.700]	319	209.542	[204.567 , 214.518]	45.169	201.960	[181.215 , 232.600]
Histidine	285	89.674	[88.309 , 91.039]	11.706	88.390	[82.120 , 96.360]	319	88.472	[87.339 , 89.604]	10.277	87.910	[81.465 , 94.160]
Isoleucine	285	69.619	[67.762 , 71.475]	15.922	67.220	[58.380 , 76.550]	319	70.281	[68.473 , 72.089]	16.417	67.910	[59.265 , 78.310]
Lactate	285	3672.067	[3587.767 , 3756.367]	723.018	3642.470	[3161.310 , 4082.520]	319	3712.537	[3625.643 , 3799.431]	788.825	3617.430	[3145.525 , 4189.495]
Leucine	285	132.286	[129.676 , 134.896]	22.384	130.690	[119.070 , 144.080]	319	134.856	[132.389 , 137.323]	22.396	132.250	[119.935 , 147.655]
Lysine	285	136.792	[134.311 , 139.273]	21.279	136.560	[122.640 , 148.290]	319	136.832	[134.448 , 139.216]	21.645	135.500	[121.070 , 151.310]
Methionine	285	27.517	[27.063 , 27.971]	3.896	27.160	[25.100 , 29.800]	319	27.731	[27.284 , 28.178]	4.058	27.280	[25.205 , 29.910]
N,N-Dimethylglycine	285	2.718	[2.585 , 2.851]	1.144	2.560	[1.960 , 3.200]	316	2.729	[2.598 , 2.861]	1.188	2.635	[2.010 , 3.250]
Ornithine	285	73.313	[71.670 , 74.956]	14.092	72.170	[63.630 , 81.100]	319	69.814	[68.319 , 71.308]	13.570	68.000	[61.090 , 78.490]
Phenylalanine	285	62.976	[61.633 , 64.318]	11.516	61.020	[55.820 , 68.570]	319	62.496	[61.312 , 63.681]	10.754	60.410	[55.080 , 67.675]
Proline	285	202.980	[197.350 , 208.609]	48.281	195.020	[170.390 , 231.390]	319	198.979	[193.119 , 204.839]	53.200	185.860	[166.295 , 220.000]
Pyruvate	285	43.014	[41.261 , 44.766]	15.034	40.110	[32.520 , 50.240]	319	44.254	[42.775 , 45.734]	13.433	42.190	[34.990 , 51.190]
Serine	285	108.851	[107.078 , 110.624]	15.205	107.560	[97.900 , 119.090]	318	108.464	[106.664 , 110.264]	16.317	106.410	[96.823 , 117.550]
Succinate	284	8.613	[8.423 , 8.803]	1.627	8.425	[7.492 , 9.660]	319	8.616	[8.447 , 8.785]	1.535	8.700	[7.545 , 9.725]
Threonine	285	199.412	[194.861 , 203.963]	39.034	200.310	[171.290 , 222.410]	319	199.007	[194.281 , 203.734]	42.904	194.190	[172.980 , 223.635]
Tryptophan	285	57.661	[56.650 , 58.672]	8.671	57.250	[52.310 , 63.230]	319	57.174	[56.040 , 58.308]	10.293	56.330	[50.930 , 62.455]
Tyrosine	285	67.295	[65.795 , 68.796]	12.869	65.580	[58.610 , 74.630]	319	67.390	[65.976 , 68.804]	12.836	65.740	[58.185 , 74.760]
Uridine	280	4.029	[3.912 , 4.145]	0.990	4.005	[3.320 , 4.520]	311	4.085	[3.985 , 4.185]	0.898	3.960	[3.470 , 4.630]
Valine	285	229.797	[225.389 , 234.205]	37.807	227.770	[204.260 , 251.000]	319	233.930	[229.815 , 238.044]	37.350	231.600	[208.055 , 255.125]
2-Aminobutyrate	285	18.972	[18.610 , 19.333]	3.101	18.780	[16.900 , 20.750]	319	19.256	[18.920 , 19.592]	3.051	19.120	[17.175 , 21.145]
2-Hydroxybutyrate	285	33.396	[31.811 , 34.981]	13.596	31.490	[24.440 , 38.860]	319	35.278	[33.694 , 36.863]	14.383	32.760	[25.350 , 42.370]
2-Oxoisocaproate	285	27.670	[26.930 , 28.411]	6.351	27.610	[23.190 , 31.670]	319	28.490	[27.736 , 29.245]	6.846	27.860	[23.545 , 32.430]
3-Hydroxybutyrate	285	58.984	[49.954 , 68.015]	77.453	36.700	[28.840 , 52.960]	319	56.396	[50.443 , 62.350]	54.042	39.110	[31.035 , 57.770]
3-Hydroxyisobutyrate	285	11.394	[10.940 , 11.847]	3.891	11.090	[8.480 , 13.440]	319	11.368	[10.992 , 11.744]	3.413	11.030	[9.055 , 13.205]
3-Methyl-2-Oxobutyric Acid	285	7.038	[6.823 , 7.252]	1.841	6.970	[5.740 , 8.190]	319	7.388	[7.166 , 7.610]	2.017	7.190	[6.050 , 8.530]
3-Methyl-2-Oxovalerate	285	22.044	[21.556 , 22.533]	4.190	21.630	[19.030 , 24.760]	319	22.593	[22.098 , 23.088]	4.493	22.130	[19.430 , 25.550]

Supplementary Table S2-2 : DateDiff1 male 40's
(3/7)

DateDiff1 male	40 - 44						45 - 49					
Name	N	Mean	95%CI	SD	Median	Quantile [lower, upper]	N	Mean	95%CI	SD	Median	Quantile [lower, upper]
Acetate	240	32.321	[28.006 , 36.635]	33.929	28.265	[25.115 , 32.240]	133	30.036	[28.495 , 31.577]	8.983	27.820	[25.220 , 31.730]
Acetone	240	5.252	[4.774 , 5.729]	3.755	4.140	[2.808 , 6.090]	134	5.340	[4.674 , 6.006]	3.895	4.070	[2.808 , 6.540]
Alanine	240	373.394	[365.502 , 381.285]	62.058	374.380	[333.688 , 418.793]	134	356.783	[345.708 , 367.858]	64.816	356.025	[316.970 , 404.117]
Arginine	240	54.243	[52.795 , 55.692]	11.392	53.350	[47.565 , 61.833]	134	52.494	[50.681 , 54.306]	10.608	51.635	[44.930 , 58.640]
Asparagine	240	57.268	[55.788 , 58.748]	11.639	55.930	[49.823 , 63.395]	134	56.780	[54.712 , 58.848]	12.102	56.750	[50.100 , 65.015]
Betaine	240	50.139	[48.545 , 51.733]	12.534	49.100	[41.508 , 57.498]	134	48.598	[46.601 , 50.594]	11.684	47.415	[41.200 , 54.508]
Carnitine	239	41.519	[40.270 , 42.769]	9.808	40.900	[35.120 , 47.230]	134	40.855	[39.183 , 42.527]	9.785	40.085	[33.418 , 47.445]
Citrate	240	90.308	[87.827 , 92.789]	19.511	87.640	[77.968 , 100.122]	134	95.828	[92.020 , 99.635]	22.284	91.935	[79.975 , 108.952]
Creatine	240	34.357	[32.646 , 36.069]	13.461	32.140	[24.137 , 41.653]	134	31.072	[29.135 , 33.010]	11.339	29.670	[21.925 , 37.395]
Creatinine	240	67.652	[66.259 , 69.045]	10.955	66.055	[61.073 , 72.872]	134	69.242	[67.574 , 70.910]	9.762	67.930	[61.875 , 76.473]
Cysteine	240	55.511	[53.508 , 57.514]	15.751	54.665	[44.348 , 62.660]	134	52.949	[50.570 , 55.329]	13.926	51.535	[42.408 , 61.987]
Formate	240	11.370	[10.904 , 11.836]	3.664	11.020	[9.468 , 12.668]	134	11.614	[11.047 , 12.181]	3.319	11.345	[9.858 , 12.950]
Glucose	240	4284.595	[4179.222 , 4389.968]	828.673	4177.085	[3842.597 , 4573.702]	134	4350.895	[4203.360 , 4498.431]	863.438	4252.275	[3870.990 , 4585.255]
Glutamate	240	68.846	[66.264 , 71.428]	20.306	65.805	[54.085 , 78.647]	134	67.021	[63.810 , 70.232]	18.792	64.560	[52.262 , 79.510]
Glutamine	240	490.004	[482.278 , 497.730]	60.758	487.360	[449.213 , 527.058]	134	492.488	[481.457 , 503.518]	64.557	498.030	[448.365 , 533.990]
Glycerol	240	61.131	[57.545 , 64.718]	28.203	54.525	[42.540 , 73.333]	134	61.512	[57.214 , 65.810]	25.153	57.255	[44.015 , 73.842]
Glycine	240	197.120	[192.727 , 201.512]	34.544	195.920	[173.893 , 217.775]	134	204.374	[196.949 , 211.800]	43.456	196.375	[174.605 , 225.720]
Histidine	240	87.972	[86.668 , 89.277]	10.258	86.705	[80.935 , 94.328]	134	86.604	[84.989 , 88.219]	9.452	85.845	[80.692 , 92.677]
Isoleucine	240	70.946	[68.853 , 73.040]	16.465	68.190	[60.508 , 76.332]	134	68.187	[65.766 , 70.609]	14.172	65.420	[59.740 , 75.150]
Lactate	240	3710.078	[3590.108 , 3830.048]	943.466	3642.745	[3122.230 , 4135.380]	134	3546.774	[3429.998 , 3663.550]	683.419	3447.485	[3070.173 , 3940.738]
Leucine	240	136.949	[134.136 , 139.762]	22.120	134.560	[122.883 , 146.450]	134	134.210	[130.284 , 138.136]	22.978	130.990	[119.395 , 144.777]
Lysine	240	134.696	[132.210 , 137.182]	19.554	131.095	[121.037 , 147.512]	134	130.643	[127.254 , 134.031]	19.832	129.960	[119.910 , 140.610]
Methionine	240	27.523	[27.076 , 27.970]	3.512	26.935	[25.307 , 29.103]	134	27.381	[26.856 , 27.906]	3.074	27.035	[25.250 , 29.365]
N,N-Dimethylglycine	240	2.778	[2.646 , 2.909]	1.033	2.675	[2.025 , 3.230]	134	2.910	[2.633 , 3.187]	1.622	2.810	[2.183 , 3.228]
Ornithine	240	69.578	[67.836 , 71.320]	13.698	68.975	[59.748 , 76.725]	134	67.643	[65.334 , 69.951]	13.510	67.825	[58.440 , 75.750]
Phenylalanine	240	62.143	[61.033 , 63.253]	8.730	61.110	[56.410 , 66.310]	134	62.224	[60.735 , 63.713]	8.712	61.410	[56.303 , 68.335]
Proline	240	190.067	[184.625 , 195.509]	42.796	182.980	[163.353 , 207.255]	134	188.960	[181.699 , 196.221]	42.496	181.705	[163.977 , 207.330]
Pyruvate	240	48.039	[44.979 , 51.099]	24.063	44.540	[36.987 , 53.872]	134	45.926	[43.604 , 48.248]	13.588	44.045	[36.985 , 51.102]
Serine	239	105.882	[104.155 , 107.608]	13.549	104.610	[96.515 , 113.660]	134	107.100	[104.458 , 109.742]	15.462	107.445	[98.060 , 118.042]
Succinate	240	8.570	[8.345 , 8.795]	1.767	8.450	[7.323 , 9.682]	134	8.570	[8.278 , 8.862]	1.709	8.540	[7.402 , 9.610]
Threonine	240	195.681	[190.781 , 200.581]	38.532	193.260	[169.542 , 217.770]	134	195.972	[189.620 , 202.325]	37.178	194.510	[170.155 , 215.345]
Tryptophan	240	56.699	[55.468 , 57.929]	9.674	55.975	[50.205 , 62.303]	134	56.855	[55.080 , 58.631]	10.390	56.140	[51.490 , 60.900]
Tyrosine	240	68.687	[67.038 , 70.335]	12.964	67.480	[59.395 , 76.150]	134	68.248	[66.136 , 70.359]	12.358	66.530	[59.365 , 74.757]
Uridine	238	4.236	[4.104 , 4.368]	1.032	4.165	[3.448 , 4.867]	133	4.334	[4.153 , 4.514]	1.051	4.280	[3.620 , 5.080]
Valine	240	235.711	[231.113 , 240.309]	36.160	231.280	[213.345 , 253.270]	134	228.916	[222.391 , 235.440]	38.184	225.610	[202.555 , 249.330]
2-Aminobutyrate	240	19.514	[19.138 , 19.889]	2.954	19.170	[17.675 , 21.015]	134	19.089	[18.510 , 19.669]	3.391	18.485	[17.025 , 20.808]
2-Hydroxybutyrate	240	38.534	[36.633 , 40.435]	14.949	36.350	[27.722 , 45.653]	134	38.501	[36.053 , 40.948]	14.325	36.020	[27.782 , 46.920]
2-Oxoisocaproate	240	29.616	[28.725 , 30.507]	7.010	29.235	[25.348 , 34.207]	134	29.046	[27.954 , 30.139]	6.395	28.400	[24.740 , 32.683]
3-Hydroxybutyrate	240	68.726	[59.660 , 77.792]	71.297	42.270	[32.872 , 70.710]	134	76.120	[64.778 , 87.462]	66.377	45.560	[33.990 , 98.787]
3-Hydroxyisobutyrate	240	11.795	[11.343 , 12.247]	3.552	11.290	[9.512 , 13.972]	134	11.466	[10.879 , 12.053]	3.435	11.110	[9.383 , 13.230]
3-Methyl-2-Oxobutyric Acid	240	7.767	[7.509 , 8.026]	2.032	7.425	[6.318 , 8.942]	134	7.718	[7.394 , 8.042]	1.895	7.545	[6.465 , 8.730]
3-Methyl-2-Oxovalerate	240	23.471	[22.821 , 24.121]	5.112	23.285	[20.008 , 26.660]	134	22.857	[22.095 , 23.620]	4.463	22.825	[19.722 , 25.720]

Supplementary Table S2-2 : DateDiff1 male 50’s
(4/7)

DateDiff1 male	50 - 54						55 - 59					
Name	N	Mean	95%CI	SD	Median	Quantile [lower, upper]	N	Mean	95%CI	SD	Median	Quantile [lower, upper]
Acetate	173	30.356	[29.272 , 31.440]	7.224	29.270	[25.730 , 33.190]	254	31.233	[29.399 , 33.067]	14.845	29.415	[25.125 , 34.045]
Acetone	173	5.859	[5.166 , 6.552]	4.615	4.450	[2.780 , 7.060]	254	6.125	[5.515 , 6.735]	4.936	4.765	[3.092 , 7.513]
Alanine	173	365.909	[355.390 , 376.427]	70.089	371.110	[315.300 , 409.610]	254	358.500	[350.717 , 366.282]	62.981	358.580	[313.125 , 395.920]
Arginine	173	55.404	[53.717 , 57.090]	11.238	56.180	[47.420 , 62.970]	254	55.362	[53.963 , 56.761]	11.323	53.965	[47.065 , 62.115]
Asparagine	173	56.333	[54.553 , 58.112]	11.859	55.470	[48.760 , 61.880]	254	56.012	[54.518 , 57.505]	12.084	53.875	[48.342 , 63.792]
Betaine	173	49.994	[48.135 , 51.853]	12.387	47.770	[41.800 , 54.980]	254	50.930	[49.234 , 52.627]	13.729	48.185	[42.325 , 57.080]
Carnitine	173	40.834	[39.412 , 42.256]	9.475	40.290	[34.780 , 46.690]	254	40.014	[38.964 , 41.064]	8.498	40.180	[33.735 , 45.733]
Citrate	173	97.287	[94.302 , 100.273]	19.896	95.940	[84.370 , 108.970]	254	94.874	[92.457 , 97.290]	19.555	93.635	[80.615 , 106.958]
Creatine	173	32.969	[31.129 , 34.808]	12.259	30.390	[23.870 , 40.080]	254	33.046	[31.338 , 34.755]	13.827	30.435	[22.992 , 39.897]
Creatinine	173	68.030	[65.841 , 70.219]	14.589	66.230	[60.680 , 73.720]	254	67.704	[66.359 , 69.049]	10.885	66.860	[60.233 , 73.230]
Cysteine	173	54.726	[52.303 , 57.150]	16.150	53.560	[44.870 , 61.600]	254	57.063	[55.323 , 58.803]	14.079	55.875	[47.748 , 64.220]
Formate	173	11.403	[10.978 , 11.828]	2.832	10.960	[9.430 , 13.410]	254	11.393	[11.003 , 11.784]	3.159	11.180	[9.253 , 13.260]
Glucose	172	4518.191	[4362.329 , 4674.053]	1035.552	4352.405	[3907.660 , 4889.663]	254	4625.376	[4445.623 , 4805.129]	1454.664	4355.185	[3948.540 , 4825.043]
Glutamate	173	67.471	[63.955 , 70.986]	23.427	62.480	[53.890 , 77.330]	254	67.380	[64.899 , 69.861]	20.078	64.855	[54.202 , 77.570]
Glutamine	173	488.341	[478.626 , 498.056]	64.736	491.230	[443.930 , 527.370]	254	477.323	[469.396 , 485.249]	64.147	479.180	[436.380 , 519.363]
Glycerol	173	63.628	[59.678 , 67.578]	26.322	59.100	[43.260 , 78.330]	254	65.595	[62.242 , 68.948]	27.137	58.600	[48.078 , 80.098]
Glycine	173	200.816	[195.084 , 206.549]	38.198	195.640	[175.690 , 221.170]	254	196.800	[191.696 , 201.905]	41.311	190.410	[168.688 , 215.773]
Histidine	173	90.918	[82.490 , 99.345]	56.156	86.600	[80.550 , 92.320]	254	85.971	[84.712 , 87.229]	10.184	86.095	[78.535 , 92.182]
Isoleucine	173	67.273	[65.232 , 69.314]	13.599	65.500	[58.550 , 74.620]	254	67.737	[65.943 , 69.531]	14.519	65.225	[57.398 , 75.397]
Lactate	173	3488.377	[3382.448 , 3594.307]	705.872	3426.600	[2979.290 , 3900.570]	254	3438.535	[3337.789 , 3539.281]	815.290	3331.205	[2936.922 , 3801.052]
Leucine	173	131.312	[128.097 , 134.526]	21.419	129.450	[116.700 , 142.680]	254	133.409	[130.467 , 136.350]	23.804	129.730	[119.422 , 148.265]
Lysine	173	133.192	[130.397 , 135.988]	18.629	131.990	[121.360 , 145.940]	254	132.837	[130.459 , 135.214]	19.243	131.465	[120.405 , 145.047]
Methionine	173	27.157	[26.670 , 27.645]	3.249	26.940	[25.120 , 29.180]	254	27.104	[26.674 , 27.533]	3.473	26.685	[25.017 , 28.995]
N,N-Dimethylglycine	173	2.817	[2.653 , 2.980]	1.091	2.690	[2.010 , 3.320]	253	2.701	[2.568 , 2.833]	1.072	2.630	[1.980 , 3.170]
Ornithine	173	66.841	[64.891 , 68.792]	12.997	65.060	[56.780 , 75.450]	254	66.550	[64.904 , 68.196]	13.320	64.410	[57.465 , 74.122]
Phenylalanine	173	62.446	[61.034 , 63.858]	9.410	60.460	[55.640 , 67.350]	254	63.534	[62.412 , 64.655]	9.075	62.305	[56.913 , 68.832]
Proline	173	183.145	[177.001 , 189.289]	40.941	177.010	[157.580 , 202.370]	254	183.069	[177.718 , 188.420]	43.304	175.090	[154.947 , 202.770]
Pyruvate	173	45.825	[43.595 , 48.056]	14.864	43.130	[36.300 , 51.940]	254	47.647	[45.308 , 49.985]	18.924	44.990	[36.752 , 55.300]
Serine	173	109.263	[106.745 , 111.780]	16.774	109.460	[97.300 , 118.220]	253	110.876	[108.857 , 112.895]	16.306	110.790	[100.660 , 121.500]
Succinate	173	8.737	[8.492 , 8.981]	1.629	8.500	[7.520 , 9.930]	254	8.708	[8.465 , 8.950]	1.961	8.520	[7.467 , 9.830]
Threonine	173	190.380	[184.089 , 196.670]	41.917	187.480	[164.440 , 210.470]	254	194.660	[189.654 , 199.667]	40.517	189.100	[168.035 , 217.860]
Tryptophan	173	54.901	[53.549 , 56.252]	9.006	53.920	[48.950 , 60.570]	254	54.697	[53.582 , 55.812]	9.025	53.740	[48.385 , 61.350]
Tyrosine	173	68.917	[66.964 , 70.871]	13.017	66.900	[59.450 , 76.830]	254	68.256	[66.711 , 69.800]	12.497	66.070	[60.255 , 75.070]
Uridine	173	4.388	[4.243 , 4.533]	0.966	4.340	[3.720 , 5.090]	253	4.358	[4.224 , 4.491]	1.079	4.320	[3.560 , 4.990]
Valine	173	227.912	[222.451 , 233.374]	36.392	226.040	[203.580 , 252.680]	254	227.943	[223.556 , 232.330]	35.503	222.475	[203.447 , 249.265]
2-Aminobutyrate	173	18.700	[18.251 , 19.150]	2.995	18.580	[16.690 , 20.350]	254	18.827	[18.455 , 19.200]	3.017	18.570	[16.850 , 20.900]
2-Hydroxybutyrate	173	40.085	[37.812 , 42.357]	15.142	37.100	[29.180 , 47.630]	254	40.351	[38.582 , 42.121]	14.321	38.385	[29.843 , 48.135]
2-Oxoisocaproate	173	28.567	[27.475 , 29.658]	7.271	28.480	[23.540 , 32.850]	254	28.650	[27.879 , 29.422]	6.245	28.150	[24.472 , 32.800]
3-Hydroxybutyrate	173	79.832	[69.320 , 90.345]	70.051	51.480	[35.890 , 102.510]	254	78.633	[69.223 , 88.043]	76.152	53.095	[34.500 , 89.362]
3-Hydroxyisobutyrate	173	11.831	[11.292 , 12.370]	3.593	11.440	[9.410 , 13.920]	254	11.788	[11.379 , 12.196]	3.305	11.545	[9.372 , 13.515]
3-Methyl-2-Oxobutyric Acid	173	7.433	[7.108 , 7.758]	2.167	7.170	[5.970 , 8.540]	254	7.545	[7.306 , 7.784]	1.933	7.285	[6.173 , 8.753]
3-Methyl-2-Oxovalerate	173	22.360	[21.634 , 23.086]	4.838	22.340	[19.020 , 24.910]	254	22.356	[21.807 , 22.905]	4.446	21.790	[19.087 , 25.377]

Supplementary Table S2-2 : DateDiff1 male 60’s
(5/7)

DateDiff1 male	60 - 64						65 - 69					
Name	N	Mean	95%CI	SD	Median	Quantile [lower, upper]	N	Mean	95%CI	SD	Median	Quantile [lower, upper]
Acetate	504	32.401	[30.120 , 34.681]	26.053	29.690	[26.065 , 34.603]	672	33.990	[32.964 , 35.016]	13.551	31.025	[27.418 , 36.788]
Acetone	504	6.946	[6.434 , 7.458]	5.846	5.050	[2.970 , 8.557]	673	6.721	[6.303 , 7.139]	5.523	4.980	[3.220 , 8.060]
Alanine	504	363.024	[357.033 , 369.014]	68.453	362.960	[312.585 , 408.922]	673	359.904	[354.767 , 365.041]	67.875	358.110	[311.980 , 407.850]
Arginine	504	54.242	[53.232 , 55.252]	11.540	53.870	[47.068 , 60.938]	673	55.405	[54.522 , 56.289]	11.669	54.980	[47.500 , 62.400]
Asparagine	504	57.935	[56.856 , 59.014]	12.328	56.925	[49.897 , 64.270]	673	58.402	[57.405 , 59.399]	13.173	57.490	[50.410 , 64.030]
Betaine	503	54.574	[53.465 , 55.683]	12.662	52.800	[45.880 , 61.290]	673	55.448	[54.385 , 56.512]	14.054	53.080	[46.450 , 61.940]
Carnitine	501	41.229	[40.447 , 42.010]	8.899	40.920	[34.950 , 46.720]	673	42.077	[41.388 , 42.766]	9.104	41.510	[36.110 , 47.030]
Citrate	504	100.796	[98.975 , 102.617]	20.808	98.405	[86.025 , 113.395]	673	104.214	[102.655 , 105.774]	20.602	102.670	[91.080 , 116.830]
Creatine	504	33.484	[32.286 , 34.683]	13.694	30.450	[23.485 , 41.565]	673	33.628	[32.606 , 34.650]	13.502	30.910	[23.640 , 41.290]
Creatinine	503	69.130	[68.149 , 70.110]	11.194	67.870	[62.095 , 75.425]	672	71.593	[70.521 , 72.666]	14.165	69.785	[62.538 , 78.177]
Cysteine	504	60.704	[59.394 , 62.014]	14.971	59.365	[50.650 , 68.917]	673	61.856	[60.783 , 62.929]	14.179	60.570	[52.530 , 70.440]
Formate	504	11.916	[11.605 , 12.228]	3.557	11.430	[9.655 , 13.705]	672	12.422	[12.127 , 12.718]	3.907	11.670	[9.908 , 14.148]
Glucose	504	4746.555	[4617.859 , 4875.250]	1470.566	4491.445	[4083.225 , 5080.570]	673	4819.509	[4723.949 , 4915.068]	1262.554	4570.470	[4109.540 , 5110.900]
Glutamate	504	66.383	[64.620 , 68.147]	20.153	63.320	[53.425 , 76.232]	673	64.109	[62.837 , 65.380]	16.804	61.510	[52.490 , 73.380]
Glutamine	504	494.136	[488.757 , 499.515]	61.464	492.725	[451.412 , 535.460]	673	501.694	[497.107 , 506.281]	60.603	501.760	[462.780 , 541.030]
Glycerol	504	72.753	[70.060 , 75.445]	30.767	66.160	[50.672 , 87.502]	673	72.863	[70.762 , 74.964]	27.759	70.020	[51.040 , 89.280]
Glycine	504	196.894	[193.587 , 200.201]	37.788	190.675	[172.562 , 212.895]	673	195.599	[192.709 , 198.489]	38.179	189.270	[170.390 , 214.660]
Histidine	504	86.148	[85.260 , 87.036]	10.145	86.205	[79.308 , 91.910]	673	86.794	[86.001 , 87.587]	10.475	85.980	[79.630 , 93.110]
Isoleucine	504	68.999	[67.598 , 70.399]	16.002	67.340	[58.715 , 76.553]	673	70.443	[69.291 , 71.596]	15.227	68.890	[60.590 , 77.940]
Lactate	504	3464.999	[3392.263 , 3537.736]	831.141	3358.850	[2959.228 , 3845.852]	673	3328.078	[3276.074 , 3380.082]	687.086	3228.170	[2824.650 , 3796.430]
Leucine	504	133.412	[131.345 , 135.479]	23.619	130.660	[118.555 , 145.852]	673	134.251	[132.584 , 135.917]	22.019	132.850	[120.430 , 145.860]
Lysine	504	135.219	[133.461 , 136.977]	20.085	133.425	[122.427 , 146.632]	673	136.174	[134.633 , 137.716]	20.372	134.370	[121.980 , 148.530]
Methionine	504	26.707	[26.402 , 27.012]	3.485	26.425	[24.278 , 28.665]	673	26.914	[26.638 , 27.190]	3.648	26.730	[24.650 , 29.100]
N,N-Dimethylglycine	500	2.824	[2.721 , 2.927]	1.176	2.605	[2.047 , 3.350]	666	2.823	[2.711 , 2.935]	1.471	2.630	[2.100 , 3.297]
Ornithine	504	68.366	[67.152 , 69.580]	13.877	66.130	[58.147 , 76.302]	673	68.536	[67.566 , 69.505]	12.812	67.920	[59.610 , 76.450]
Phenylalanine	504	64.427	[63.613 , 65.241]	9.302	63.025	[58.547 , 69.198]	673	66.190	[65.437 , 66.943]	9.950	64.790	[59.650 , 70.630]
Proline	503	183.751	[180.241 , 187.260]	40.060	177.460	[158.290 , 204.610]	673	184.484	[181.524 , 187.443]	39.104	179.540	[157.750 , 204.530]
Pyruvate	504	48.671	[46.863 , 50.479]	20.658	44.635	[37.940 , 53.790]	673	46.285	[45.223 , 47.347]	14.032	43.870	[36.930 , 52.260]
Serine	504	110.415	[108.936 , 111.894]	16.904	108.260	[99.300 , 121.480]	672	110.794	[109.541 , 112.047]	16.539	108.865	[99.730 , 120.840]
Succinate	504	8.925	[8.748 , 9.102]	2.025	8.735	[7.530 , 10.002]	673	8.927	[8.794 , 9.059]	1.750	8.750	[7.730 , 9.900]
Threonine	504	193.010	[189.178 , 196.843]	43.795	192.565	[165.763 , 218.405]	673	188.821	[185.827 , 191.815]	39.557	185.740	[163.040 , 212.370]
Tryptophan	504	55.160	[54.281 , 56.038]	10.039	54.285	[48.975 , 60.385]	673	55.385	[54.642 , 56.127]	9.811	55.390	[49.070 , 61.120]
Tyrosine	504	70.754	[69.551 , 71.956]	13.742	68.635	[61.443 , 78.645]	673	72.163	[71.061 , 73.266]	14.565	69.280	[62.480 , 80.480]
Uridine	495	4.398	[4.313 , 4.483]	0.965	4.390	[3.735 , 4.965]	667	4.401	[4.327 , 4.476]	0.979	4.380	[3.720 , 4.995]
Valine	504	231.042	[227.820 , 234.264]	36.818	227.745	[205.180 , 253.172]	673	232.886	[230.103 , 235.669]	36.772	229.290	[209.140 , 253.010]
2-Aminobutyrate	504	19.155	[18.890 , 19.420]	3.029	18.700	[17.168 , 21.005]	673	19.378	[19.150 , 19.607]	3.022	19.120	[17.450 , 21.090]
2-Hydroxybutyrate	504	42.740	[40.928 , 44.553]	20.714	39.755	[31.328 , 50.980]	673	41.706	[40.575 , 42.837]	14.946	39.740	[31.310 , 51.020]
2-Oxoisocaproate	504	28.994	[28.397 , 29.592]	6.827	28.910	[24.348 , 33.153]	673	29.011	[28.526 , 29.495]	6.400	28.800	[24.840 , 32.890]
3-Hydroxybutyrate	504	101.389	[92.641 , 110.138]	99.968	61.615	[35.890 , 127.595]	673	98.930	[91.831 , 106.029]	93.791	61.930	[36.710 , 135.000]
3-Hydroxyisobutyrate	504	12.152	[11.793 , 12.510]	4.094	11.615	[9.835 , 13.560]	673	12.592	[12.310 , 12.873]	3.715	11.960	[9.910 , 14.760]
3-Methyl-2-Oxobutyric Acid	504	7.642	[7.475 , 7.809]	1.911	7.555	[6.297 , 8.940]	673	7.663	[7.519 , 7.807]	1.904	7.540	[6.420 , 8.840]
3-Methyl-2-Oxovalerate	504	22.454	[22.035 , 22.874]	4.794	22.195	[19.380 , 25.310]	673	22.622	[22.285 , 22.960]	4.460	22.510	[19.450 , 25.590]

Supplementary Table S2-2 : DateDiff1 male 70’s
(6/7)

DateDiff1 male	70 - 74						75 - 79					
Name	N	Mean	95%CI	SD	Median	Quantile [lower, upper]	N	Mean	95%CI	SD	Median	Quantile [lower, upper]
Acetate	443	36.560	[31.882 , 41.238]	50.094	32.100	[27.820 , 37.550]	236	35.370	[34.004 , 36.736]	10.653	32.485	[27.992 , 39.782]
Acetone	443	6.865	[6.316 , 7.414]	5.880	4.960	[3.100 , 8.255]	236	6.356	[5.735 , 6.976]	4.838	5.050	[3.178 , 7.525]
Alanine	443	359.554	[353.049 , 366.059]	69.664	356.980	[310.675 , 405.740]	236	359.033	[349.913 , 368.152]	71.111	353.735	[307.650 , 409.525]
Arginine	443	56.449	[55.400 , 57.499]	11.242	55.910	[49.370 , 64.090]	236	57.175	[55.680 , 58.670]	11.659	56.070	[49.992 , 65.158]
Asparagine	443	58.518	[57.305 , 59.732]	12.997	57.370	[51.010 , 65.130]	236	58.709	[56.941 , 60.478]	13.790	57.610	[48.828 , 66.150]
Betaine	443	57.234	[55.843 , 58.625]	14.897	54.530	[46.700 , 64.480]	236	55.895	[54.065 , 57.725]	14.268	54.495	[45.930 , 63.795]
Carnitine	442	40.922	[40.080 , 41.764]	9.006	40.755	[34.845 , 47.163]	235	40.024	[38.835 , 41.213]	9.250	40.190	[34.105 , 45.985]
Citrate	443	107.424	[105.395 , 109.453]	21.729	105.310	[93.080 , 121.510]	236	110.240	[107.324 , 113.156]	22.737	108.470	[95.082 , 123.542]
Creatine	443	33.153	[31.903 , 34.403]	13.389	30.480	[23.630 , 40.100]	236	31.808	[29.921 , 33.695]	14.714	27.875	[21.802 , 39.590]
Creatinine	442	72.517	[71.017 , 74.016]	16.042	70.190	[62.830 , 79.428]	236	72.895	[69.906 , 75.884]	23.306	69.540	[61.600 , 78.645]
Cysteine	443	62.727	[61.392 , 64.062]	14.299	61.900	[52.680 , 70.290]	236	60.654	[58.670 , 62.639]	15.475	59.725	[49.125 , 69.007]
Formate	443	13.961	[13.420 , 14.503]	5.802	12.600	[10.595 , 15.435]	236	14.341	[13.563 , 15.119]	6.065	13.100	[10.405 , 16.185]
Glucose	443	4920.324	[4802.345 , 5038.304]	1263.488	4677.510	[4254.670 , 5190.080]	236	4979.617	[4811.517 , 5147.716]	1310.784	4636.915	[4169.568 , 5331.183]
Glutamate	443	61.389	[59.928 , 62.849]	15.638	58.160	[50.685 , 70.370]	236	58.894	[56.863 , 60.924]	15.831	57.265	[47.565 , 65.795]
Glutamine	443	507.599	[501.454 , 513.743]	65.807	507.930	[467.840 , 544.770]	236	509.154	[499.408 , 518.900]	75.999	505.685	[463.940 , 546.485]
Glycerol	443	74.888	[72.071 , 77.704]	30.159	69.830	[51.135 , 93.955]	236	77.081	[72.851 , 81.311]	32.987	70.520	[52.110 , 96.152]
Glycine	443	190.687	[187.571 , 193.803]	33.374	188.290	[168.655 , 208.005]	236	189.140	[184.671 , 193.608]	34.844	184.990	[167.200 , 204.882]
Histidine	443	87.037	[85.861 , 88.214]	12.599	85.800	[79.390 , 92.395]	236	85.588	[84.113 , 87.064]	11.507	84.775	[77.635 , 92.908]
Isoleucine	443	71.132	[69.681 , 72.584]	15.548	69.190	[60.450 , 79.220]	236	71.236	[69.086 , 73.386]	16.761	68.685	[60.335 , 78.845]
Lactate	443	3177.137	[3113.148 , 3241.127]	685.285	3088.030	[2693.215 , 3644.995]	236	3065.794	[2982.887 , 3148.700]	646.476	2996.095	[2618.372 , 3503.757]
Leucine	443	132.395	[130.358 , 134.431]	21.810	131.470	[117.535 , 145.055]	236	129.286	[126.106 , 132.466]	24.795	125.370	[114.215 , 141.255]
Lysine	443	136.842	[134.879 , 138.806]	21.026	134.520	[123.390 , 147.740]	236	135.778	[132.774 , 138.782]	23.424	134.125	[121.535 , 146.743]
Methionine	443	27.164	[26.817 , 27.512]	3.724	26.870	[24.645 , 29.140]	236	27.530	[27.043 , 28.016]	3.795	27.250	[24.590 , 29.730]
N,N-Dimethylglycine	440	2.902	[2.777 , 3.026]	1.326	2.640	[2.188 , 3.320]	236	2.818	[2.675 , 2.960]	1.110	2.685	[2.060 , 3.342]
Ornithine	443	69.570	[68.300 , 70.840]	13.599	67.950	[60.850 , 75.690]	236	70.143	[68.284 , 72.001]	14.492	69.520	[60.185 , 77.963]
Phenylalanine	443	67.181	[66.152 , 68.210]	11.018	65.380	[60.055 , 72.390]	236	68.057	[66.599 , 69.516]	11.372	65.575	[60.397 , 74.725]
Proline	443	185.935	[181.602 , 190.268]	46.406	178.760	[157.690 , 203.980]	236	182.745	[178.010 , 187.479]	36.920	175.375	[157.067 , 203.070]
Pyruvate	443	48.405	[46.573 , 50.237]	19.620	45.060	[37.585 , 54.420]	236	45.881	[44.165 , 47.596]	13.378	44.025	[37.285 , 52.752]
Serine	443	111.672	[110.064 , 113.281]	17.228	109.770	[99.965 , 121.775]	236	111.209	[108.965 , 113.453]	17.495	108.880	[98.930 , 123.225]
Succinate	443	8.987	[8.816 , 9.158]	1.833	8.790	[7.750 , 9.945]	236	8.829	[8.583 , 9.075]	1.917	8.665	[7.503 , 9.762]
Threonine	443	186.070	[182.149 , 189.991]	41.995	183.800	[157.240 , 210.710]	236	180.281	[175.602 , 184.959]	36.483	183.130	[155.965 , 203.000]
Tryptophan	443	54.811	[53.899 , 55.722]	9.761	54.370	[48.410 , 61.435]	236	53.513	[52.164 , 54.862]	10.519	53.265	[46.447 , 59.793]
Tyrosine	443	73.324	[71.892 , 74.755]	15.333	72.260	[62.860 , 80.980]	236	73.823	[71.615 , 76.032]	17.221	70.975	[61.890 , 81.882]
Uridine	439	4.331	[4.240 , 4.422]	0.971	4.310	[3.635 , 4.880]	232	4.444	[4.308 , 4.579]	1.047	4.410	[3.730 , 5.090]
Valine	443	235.791	[232.270 , 239.311]	37.703	232.240	[210.780 , 257.120]	236	234.595	[229.313 , 239.877]	41.190	229.690	[205.167 , 258.388]
2-Aminobutyrate	443	19.539	[19.250 , 19.828]	3.095	19.220	[17.465 , 21.365]	236	19.148	[18.713 , 19.582]	3.385	18.860	[16.718 , 21.040]
2-Hydroxybutyrate	443	41.087	[39.725 , 42.449]	14.584	39.830	[30.845 , 47.710]	236	39.040	[37.279 , 40.801]	13.733	36.650	[29.468 , 46.073]
2-Oxoisocaproate	443	28.544	[27.967 , 29.122]	6.183	28.180	[24.375 , 32.015]	236	26.988	[26.220 , 27.756]	5.989	26.425	[22.828 , 30.320]
3-Hydroxybutyrate	443	102.216	[93.311 , 111.121]	95.364	64.980	[38.035 , 135.910]	236	98.166	[86.782 , 109.551]	88.775	63.180	[34.885 , 133.955]
3-Hydroxyisobutyrate	443	12.909	[12.562 , 13.257]	3.723	12.440	[10.530 , 14.875]	236	12.897	[12.404 , 13.390]	3.842	12.225	[10.130 , 14.923]
3-Methyl-2-Oxobutyric Acid	443	7.702	[7.538 , 7.866]	1.756	7.590	[6.480 , 8.835]	236	7.370	[7.133 , 7.607]	1.844	7.065	[6.000 , 8.528]
3-Methyl-2-Oxovalerate	443	22.497	[22.079 , 22.914]	4.471	21.950	[19.440 , 25.065]	236	21.653	[21.135 , 22.170]	4.034	21.395	[19.028 , 23.952]

Supplementary Table S2-2 : DateDiff1 male Over 80's
(7/7)

DateDiff1 male	over 80						
Name	N	Mean	95%CI	SD	Median	Quantile [lower, upper]
Acetate	105	34.628	[33.009 , 36.247]	8.365	33.280	[29.180 , 38.470]
Acetone	105	6.107	[5.232 , 6.982]	4.522	4.980	[2.930 , 7.740]
Alanine	105	346.289	[331.060 , 361.518]	78.692	344.600	[278.010 , 396.950]
Arginine	105	55.787	[53.523 , 58.051]	11.698	56.880	[49.500 , 62.290]
Asparagine	105	58.311	[55.991 , 60.631]	11.987	57.760	[51.470 , 63.900]
Betaine	105	56.078	[52.858 , 59.297]	16.635	54.410	[47.430 , 60.560]
Carnitine	105	38.739	[36.842 , 40.637]	9.805	37.350	[32.890 , 46.680]
Citrate	105	122.280	[117.150 , 127.409]	26.505	119.950	[103.100 , 136.470]
Creatine	105	31.285	[28.729 , 33.840]	13.205	28.300	[22.400 , 38.210]
Creatinine	105	78.790	[75.405 , 82.176]	17.494	77.660	[65.480 , 90.000]
Cysteine	105	60.575	[57.818 , 63.332]	14.247	60.670	[50.400 , 69.100]
Formate	105	14.964	[13.541 , 16.388]	7.357	12.560	[10.600 , 17.370]
Glucose	105	4956.591	[4649.179 , 5264.004]	1588.492	4548.630	[4239.650 , 4998.770]
Glutamate	105	57.047	[54.161 , 59.933]	14.911	55.320	[47.020 , 65.010]
Glutamine	105	506.523	[495.113 , 517.933]	58.959	508.100	[465.360 , 551.550]
Glycerol	105	77.916	[71.368 , 84.464]	33.836	69.510	[51.380 , 96.490]
Glycine	105	191.528	[185.415 , 197.640]	31.586	188.270	[165.700 , 213.280]
Histidine	105	82.454	[80.436 , 84.473]	10.432	81.580	[74.170 , 89.370]
Isoleucine	105	67.275	[63.925 , 70.626]	17.314	62.930	[55.440 , 76.230]
Lactate	105	3184.056	[3049.237 , 3318.876]	696.651	3184.820	[2602.200 , 3641.910]
Leucine	105	123.063	[118.612 , 127.513]	22.999	120.290	[108.540 , 135.750]
Lysine	105	132.632	[128.656 , 136.607]	20.542	133.890	[116.100 , 142.260]
Methionine	105	26.402	[25.686 , 27.118]	3.697	26.270	[23.830 , 27.880]
N,N-Dimethylglycine	105	3.120	[2.921 , 3.319]	1.028	2.890	[2.460 , 3.890]
Ornithine	105	68.787	[66.292 , 71.283]	12.894	66.420	[60.190 , 75.180]
Phenylalanine	105	68.950	[66.735 , 71.164]	11.442	66.740	[61.710 , 75.650]
Proline	105	182.199	[172.741 , 191.657]	48.873	175.020	[145.640 , 204.810]
Pyruvate	105	47.387	[45.101 , 49.673]	11.811	45.470	[38.360 , 54.610]
Serine	105	111.146	[108.027 , 114.265]	16.118	109.860	[98.730 , 122.080]
Succinate	105	9.622	[9.222 , 10.021]	2.063	9.390	[8.120 , 10.700]
Threonine	105	176.583	[169.589 , 183.576]	36.138	174.630	[149.200 , 206.560]
Tryptophan	105	51.662	[49.487 , 53.837]	11.239	50.810	[44.380 , 57.670]
Tyrosine	105	71.396	[68.531 , 74.260]	14.801	69.890	[60.420 , 80.050]
Uridine	105	4.214	[4.027 , 4.400]	0.961	4.230	[3.620 , 4.820]
Valine	105	219.627	[212.087 , 227.167]	38.961	215.480	[191.400 , 245.570]
2-Aminobutyrate	105	18.253	[17.616 , 18.890]	3.290	17.990	[16.000 , 20.270]
2-Hydroxybutyrate	105	38.074	[35.794 , 40.355]	11.783	37.560	[28.820 , 45.430]
2-Oxoisocaproate	105	26.346	[25.183 , 27.508]	6.006	25.980	[21.960 , 30.150]
3-Hydroxybutyrate	105	106.541	[89.372 , 123.710]	88.718	73.540	[42.680 , 151.000]
3-Hydroxyisobutyrate	105	12.434	[11.698 , 13.170]	3.805	11.400	[9.710 , 14.120]
3-Methyl-2-Oxobutyric Acid	105	7.342	[6.971 , 7.714]	1.921	7.350	[5.920 , 8.570]
3-Methyl-2-Oxovalerate	105	21.260	[20.339 , 22.181]	4.760	20.790	[18.270 , 24.590]

Supplementary Table S2-3 : DateDiff0 female 20’s
(1/7)

DateDiff0 female	20 - 24						25 - 29					
Name	N	Mean	95%CI	SD	Median	Quantile [lower, upper]	N	Mean	95%CI	SD	Median	Quantile [lower, upper]
Acetate	138	30.578	[29.640 , 31.516]	5.572	30.085	[26.685 , 33.910]	355	32.157	[31.331 , 32.982]	7.908	30.840	[26.600 , 36.375]
Acetone	138	4.808	[4.105 , 5.510]	4.176	3.710	[2.050 , 5.478]	355	5.290	[4.748 , 5.831]	5.187	3.620	[2.170 , 6.255]
Alanine	138	329.480	[316.028 , 342.932]	79.915	324.995	[275.638 , 365.305]	355	317.451	[309.333 , 325.569]	77.771	309.160	[263.555 , 357.355]
Arginine	138	44.227	[42.777 , 45.677]	8.615	44.285	[38.580 , 48.905]	355	44.435	[43.219 , 45.651]	11.647	43.580	[37.325 , 50.830]
Asparagine	138	58.062	[56.016 , 60.108]	12.155	56.445	[50.330 , 65.460]	355	57.214	[55.945 , 58.484]	12.161	55.600	[48.325 , 63.695]
Betaine	138	41.208	[39.255 , 43.162]	11.606	39.515	[32.125 , 48.000]	355	40.300	[39.203 , 41.398]	10.517	39.390	[33.520 , 45.705]
Carnitine	136	34.328	[33.063 , 35.593]	7.460	33.710	[29.280 , 39.942]	343	34.071	[33.289 , 34.853]	7.365	33.540	[28.855 , 39.055]
Citrate	138	99.967	[96.895 , 103.039]	18.249	99.660	[87.688 , 113.395]	355	99.818	[97.691 , 101.945]	20.375	96.740	[85.380 , 114.150]
Creatine	138	33.121	[31.123 , 35.118]	11.866	31.910	[24.135 , 40.300]	355	35.645	[34.293 , 36.996]	12.948	34.400	[26.715 , 42.775]
Creatinine	138	50.659	[49.407 , 51.911]	7.438	50.015	[44.850 , 55.252]	355	49.652	[48.940 , 50.363]	6.813	48.960	[44.765 , 54.145]
Cysteine	138	44.666	[43.214 , 46.119]	8.629	43.955	[38.695 , 50.388]	355	45.974	[45.028 , 46.919]	9.059	45.490	[40.130 , 50.975]
Formate	136	10.896	[10.352 , 11.440]	3.207	10.340	[8.780 , 12.525]	348	11.301	[10.922 , 11.680]	3.591	10.665	[9.125 , 12.475]
Glucose	138	4423.598	[4326.856 , 4520.340]	574.717	4424.120	[4083.872 , 4686.253]	355	4511.178	[4454.181 , 4568.176]	546.055	4493.950	[4155.130 , 4835.520]
Glutamate	138	38.877	[36.875 , 40.878]	11.891	37.385	[29.975 , 44.680]	355	41.738	[40.529 , 42.948]	11.589	39.650	[33.565 , 47.370]
Glutamine	138	433.682	[423.915 , 443.450]	58.026	431.155	[399.620 , 481.575]	355	433.862	[426.940 , 440.783]	66.311	432.320	[389.020 , 480.290]
Glycerol	138	68.700	[63.591 , 73.808]	30.348	61.595	[44.942 , 85.088]	355	69.754	[66.200 , 73.307]	34.041	60.610	[46.085 , 85.320]
Glycine	138	205.240	[197.689 , 212.791]	44.859	203.715	[171.865 , 230.290]	355	213.146	[207.663 , 218.630]	52.534	206.330	[180.805 , 234.950]
Histidine	138	82.484	[80.622 , 84.346]	11.062	81.895	[76.335 , 86.878]	355	82.725	[81.677 , 83.773]	10.040	81.990	[76.665 , 87.500]
Isoleucine	138	56.525	[53.760 , 59.291]	16.431	52.420	[47.823 , 61.678]	355	54.964	[53.401 , 56.527]	14.975	52.050	[44.880 , 60.810]
Lactate	138	2202.081	[2099.313 , 2304.848]	610.514	2089.165	[1743.652 , 2545.360]	355	2211.678	[2158.149 , 2265.207]	512.821	2134.450	[1869.795 , 2520.130]
Leucine	138	105.207	[101.443 , 108.972]	22.364	102.245	[92.422 , 113.135]	355	104.271	[102.140 , 106.403]	20.420	102.010	[89.935 , 114.950]
Lysine	138	119.340	[116.319 , 122.362]	17.949	120.000	[107.958 , 129.230]	355	121.441	[119.118 , 123.764]	22.251	119.270	[107.480 , 132.230]
Methionine	138	26.124	[25.429 , 26.820]	4.131	25.805	[23.712 , 27.670]	355	25.990	[25.528 , 26.452]	4.429	25.640	[23.600 , 27.965]
N,N-Dimethylglycine	138	2.439	[2.263 , 2.616]	1.050	2.285	[1.750 , 2.967]	355	2.331	[2.235 , 2.427]	0.918	2.230	[1.705 , 2.770]
Ornithine	138	59.701	[57.345 , 62.058]	14.000	57.925	[48.370 , 69.755]	355	61.980	[60.437 , 63.522]	14.781	60.700	[51.455 , 71.555]
Phenylalanine	138	56.809	[55.049 , 58.568]	10.452	54.130	[49.880 , 60.272]	355	56.484	[55.480 , 57.487]	9.610	55.430	[49.555 , 60.885]
Proline	138	156.984	[151.476 , 162.492]	32.722	150.270	[130.793 , 177.495]	355	154.464	[150.257 , 158.671]	40.302	147.470	[129.340 , 166.925]
Pyruvate	138	88.846	[83.057 , 94.634]	34.389	84.780	[63.993 , 108.408]	355	95.760	[92.274 , 99.246]	33.398	94.620	[73.055 , 116.935]
Serine	138	111.812	[109.305 , 114.318]	14.890	111.370	[102.688 , 121.190]	355	112.735	[111.121 , 114.350]	15.469	112.450	[101.210 , 122.695]
Succinate	138	7.058	[6.811 , 7.304]	1.466	6.790	[6.018 , 7.650]	355	7.156	[6.970 , 7.342]	1.785	7.030	[6.100 , 8.035]
Threonine	138	180.002	[173.328 , 186.675]	39.646	175.555	[155.472 , 206.570]	355	182.272	[177.682 , 186.861]	43.966	180.000	[152.480 , 208.140]
Tryptophan	138	52.692	[50.715 , 54.670]	11.749	50.680	[45.500 , 58.100]	355	51.123	[50.102 , 52.143]	9.777	50.330	[44.910 , 57.100]
Tyrosine	138	60.716	[58.245 , 63.188]	14.683	57.105	[51.098 , 68.375]	355	60.668	[59.202 , 62.134]	14.042	58.070	[52.050 , 66.930]
Uridine	136	3.703	[3.557 , 3.849]	0.861	3.620	[3.105 , 4.232]	353	3.870	[3.783 , 3.957]	0.831	3.850	[3.270 , 4.440]
Valine	138	194.210	[187.379 , 201.041]	40.580	185.870	[170.602 , 208.055]	355	190.592	[186.726 , 194.459]	37.043	185.580	[165.240 , 207.230]
2-Aminobutyrate	138	16.061	[15.484 , 16.638]	3.428	15.485	[14.050 , 17.442]	355	15.876	[15.558 , 16.195]	3.048	15.430	[13.685 , 17.600]
2-Hydroxybutyrate	138	33.835	[31.719 , 35.951]	12.572	31.345	[24.940 , 39.255]	355	34.180	[32.758 , 35.601]	13.619	32.160	[24.715 , 41.020]
2-Oxoisocaproate	138	27.695	[26.635 , 28.755]	6.298	27.490	[23.218 , 31.625]	355	26.632	[26.031 , 27.234]	5.764	26.480	[22.690 , 29.840]
3-Hydroxybutyrate	138	84.828	[70.501 , 99.154]	85.109	49.850	[32.070 , 95.080]	355	98.916	[86.619 , 111.213]	117.809	55.240	[33.555 , 113.945]
3-Hydroxyisobutyrate	138	10.665	[10.049 , 11.280]	3.655	10.130	[8.057 , 12.418]	355	10.417	[10.048 , 10.786]	3.532	9.680	[8.325 , 11.895]
3-Methyl-2-Oxobutyric Acid	138	9.262	[8.911 , 9.613]	2.087	9.195	[7.738 , 10.738]	355	9.148	[8.944 , 9.352]	1.956	9.040	[7.875 , 10.280]
3-Methyl-2-Oxovalerate	138	20.985	[20.239 , 21.731]	4.432	20.910	[18.012 , 23.425]	355	20.020	[19.568 , 20.471]	4.324	19.430	[16.855 , 22.985]

Supplementary Table S2-3 : DateDiff0 female 30's
(2/7)

DateDiff0 female	30 - 34								35 - 39							
Name	N	Mean	95%CI		SD	Median	Quantile [lower, upper]		N	Mean	95%CI		SD	Median	Quantile [lower, upper]	
Acetate	785	32.001	31.462	32.540	7.691	30.880	26.880	35.180	1153	31.666	31.043	32.290	10.794	30.020	26.410	35.170
Acetone	785	4.958	4.616	5.300	4.881	3.390	2.090	5.990	1153	5.226	4.939	5.512	4.954	3.580	2.170	6.280
Alanine	785	314.109	308.654	319.563	77.857	306.300	257.740	362.640	1153	307.494	303.034	311.954	77.188	295.920	249.790	352.690
Arginine	785	44.178	43.400	44.955	11.097	43.610	37.550	51.280	1153	43.342	42.727	43.958	10.652	43.250	36.340	49.470
Asparagine	785	58.014	57.083	58.946	13.291	56.880	49.980	65.060	1153	58.153	57.392	58.915	13.178	56.770	49.840	64.560
Betaine	785	41.926	41.168	42.683	10.807	41.000	34.470	48.010	1153	40.817	40.199	41.435	10.691	39.500	33.990	46.780
Carnitine	764	33.596	33.025	34.166	8.035	32.935	28.305	38.392	1122	32.989	32.539	33.438	7.670	32.495	27.622	38.010
Citrate	785	101.654	100.135	103.172	21.670	99.720	85.520	116.260	1153	102.127	100.932	103.322	20.684	99.670	87.820	115.710
Creatine	785	38.170	37.219	39.121	13.571	36.590	28.240	46.300	1153	36.284	35.516	37.052	13.296	34.420	26.130	44.150
Creatinine	785	50.537	50.045	51.028	7.014	50.170	45.560	54.710	1152	51.135	50.697	51.573	7.576	50.555	45.948	55.710
Cysteine	785	47.402	46.680	48.124	10.303	46.570	40.850	52.460	1153	47.876	47.303	48.449	9.917	46.940	41.210	53.480
Formate	769	10.760	10.544	10.977	3.061	10.210	8.820	12.250	1132	10.758	10.581	10.936	3.041	10.380	8.818	12.210
Glucose	785	4568.271	4527.428	4609.113	582.944	4507.880	4210.290	4846.190	1153	4581.241	4541.051	4621.432	695.560	4496.830	4177.740	4838.780
Glutamate	785	40.993	40.183	41.803	11.558	39.250	32.880	47.550	1153	42.125	41.403	42.847	12.501	39.440	33.540	48.900
Glutamine	785	441.519	436.897	446.141	65.965	441.780	399.790	482.280	1153	437.998	433.871	442.124	71.415	435.870	391.360	485.580
Glycerol	785	68.529	66.074	70.984	35.038	59.480	43.100	85.600	1153	66.889	65.029	68.750	32.198	59.060	43.960	83.860
Glycine	785	227.938	223.162	232.714	68.172	211.850	182.510	258.650	1153	224.406	220.581	228.231	66.198	210.820	178.130	255.310
Histidine	785	82.822	82.104	83.541	10.248	81.930	76.060	89.210	1153	82.197	81.617	82.777	10.032	81.450	75.430	87.870
Isoleucine	785	53.452	52.505	54.400	13.521	50.770	44.440	59.300	1153	51.082	50.301	51.864	13.521	48.400	42.410	55.910
Lactate	785	2184.782	2145.459	2224.106	561.264	2102.780	1779.560	2500.340	1153	2214.286	2182.452	2246.120	550.933	2153.570	1822.620	2530.740
Leucine	785	101.905	100.623	103.187	18.295	99.240	89.860	111.390	1153	99.333	98.228	100.438	19.120	96.140	86.680	107.630
Lysine	785	122.463	121.009	123.917	20.752	121.440	107.440	134.600	1153	119.657	118.454	120.859	20.814	118.210	104.950	132.880
Methionine	785	25.714	25.424	26.003	4.130	25.390	23.110	27.720	1153	25.171	24.937	25.405	4.048	24.930	22.770	27.200
N,N-Dimethylglycine	783	2.220	2.160	2.280	0.861	2.120	1.650	2.615	1147	2.189	2.145	2.234	0.771	2.140	1.620	2.625
Ornithine	785	62.343	61.323	63.362	14.550	61.400	52.070	70.810	1153	62.806	61.952	63.661	14.783	61.120	52.210	72.460
Phenylalanine	785	55.988	55.312	56.664	9.649	54.120	49.300	60.880	1153	54.856	54.325	55.387	9.185	53.340	48.680	59.400
Proline	785	150.648	148.089	153.208	36.534	144.390	126.030	168.650	1153	147.022	144.881	149.164	37.058	140.330	121.730	163.910
Pyruvate	785	96.338	94.061	98.615	32.501	94.970	73.270	116.970	1153	98.361	96.463	100.259	32.843	96.810	78.240	117.560
Serine	785	113.363	112.140	114.585	17.454	112.970	101.700	124.630	1152	110.917	109.943	111.890	16.836	109.885	99.998	121.100
Succinate	785	7.446	7.316	7.577	1.864	7.170	6.200	8.460	1153	7.624	7.512	7.736	1.940	7.420	6.330	8.600
Threonine	785	175.246	172.250	178.242	42.759	173.000	146.160	201.830	1153	174.919	172.239	177.599	46.385	172.690	143.620	202.540
Tryptophan	785	50.498	49.867	51.129	9.011	50.130	44.650	55.920	1153	49.095	48.584	49.606	8.841	48.550	43.350	54.480
Tyrosine	785	60.016	59.112	60.921	12.910	57.760	51.370	66.770	1153	59.399	58.594	60.203	13.924	57.320	50.210	65.770
Uridine	770	3.941	3.877	4.006	0.907	3.880	3.330	4.520	1138	3.906	3.855	3.958	0.887	3.860	3.280	4.460
Valine	785	186.763	184.314	189.212	34.954	181.080	163.730	204.860	1153	179.860	177.911	181.808	33.722	175.320	156.980	197.260
2-Aminobutyrate	785	15.511	15.311	15.710	2.845	15.080	13.600	17.060	1153	15.032	14.870	15.193	2.796	14.660	13.070	16.460
2-Hydroxybutyrate	785	32.912	31.903	33.921	14.404	29.830	23.150	39.630	1152	34.000	33.223	34.778	13.449	31.795	25.135	40.712
2-Oxoisocaproate	785	25.210	24.830	25.591	5.426	24.790	21.160	28.570	1153	24.463	24.161	24.764	5.220	24.090	20.760	27.460
3-Hydroxybutyrate	784	98.552	91.130	105.975	105.878	55.935	31.253	131.215	1152	99.801	94.368	105.234	93.986	67.815	36.422	128.120
3-Hydroxyisobutyrate	785	10.068	9.840	10.295	3.242	9.460	7.940	11.500	1153	10.031	9.857	10.205	3.005	9.550	7.960	11.730
3-Methyl-2-Oxobutyric Acid	785	8.840	8.702	8.977	1.969	8.680	7.430	10.060	1153	8.611	8.505	8.717	1.839	8.500	7.340	9.770
3-Methyl-2-Oxovalerate	785	18.963	18.698	19.228	3.784	18.450	16.250	21.300	1153	18.306	18.089	18.524	3.765	17.920	15.640	20.460

Supplementary Table S2-3 : DateDiff0 female 40’s
(3/7)

DateDiff0 female	40 - 44								45 - 49							
Name	N	Mean	95%CI		SD	Median	Quantile [lower, upper]		N	Mean	95%CI		SD	Median	Quantile [lower, upper]	
Acetate	1018	33.021	32.025	34.017	16.195	31.065	26.508	36.338	1083	32.744	32.260	33.228	8.113	31.370	27.280	36.515
Acetone	1019	5.597	5.300	5.893	4.824	4.110	2.490	7.320	1083	6.267	5.934	6.600	5.585	4.630	2.585	8.065
Alanine	1019	308.280	303.362	313.198	80.004	298.040	249.410	351.900	1083	315.496	310.610	320.382	81.953	303.720	254.695	363.325
Arginine	1019	44.785	44.105	45.464	11.060	43.770	37.390	51.045	1083	45.598	44.986	46.209	10.256	44.900	38.930	51.200
Asparagine	1019	58.770	57.873	59.668	14.600	57.370	49.650	66.100	1082	58.900	58.082	59.717	13.703	56.915	49.932	65.440
Betaine	1019	40.738	40.080	41.396	10.705	39.850	33.730	46.385	1083	41.093	40.425	41.762	11.210	39.620	33.740	46.840
Carnitine	1011	33.746	33.286	34.206	7.454	33.350	28.465	38.230	1080	34.688	34.243	35.133	7.452	34.190	29.605	39.472
Citrate	1019	104.174	102.855	105.492	21.447	102.260	89.150	117.155	1083	107.407	106.143	108.671	21.199	104.410	92.135	119.800
Creatine	1019	36.474	35.599	37.349	14.235	34.600	26.320	44.265	1083	37.805	37.016	38.595	13.247	36.850	28.085	45.695
Creatinine	1019	51.736	51.268	52.203	7.600	51.300	46.695	56.130	1083	52.637	52.105	53.169	8.921	51.950	47.240	57.510
Cysteine	1019	48.450	47.828	49.072	10.120	47.530	41.930	54.510	1083	51.090	50.443	51.738	10.860	49.840	44.030	56.530
Formate	1019	10.748	10.567	10.929	2.944	10.370	8.915	12.070	1083	10.611	10.439	10.783	2.883	10.240	8.845	11.955
Glucose	1019	4735.030	4685.471	4784.589	806.206	4622.090	4322.735	5005.430	1082	4881.395	4823.283	4939.508	974.205	4753.225	4431.760	5094.728
Glutamate	1019	41.791	41.001	42.580	12.843	39.450	32.685	48.560	1083	44.799	43.982	45.616	13.709	42.390	35.310	51.805
Glutamine	1019	443.658	439.507	447.810	67.538	443.070	397.740	487.830	1083	459.383	455.347	463.419	67.691	458.930	412.495	503.310
Glycerol	1019	72.025	70.021	74.028	32.590	67.930	47.155	90.875	1083	74.931	72.922	76.940	33.701	69.960	49.550	94.040
Glycine	1019	221.659	217.553	225.766	66.801	207.400	176.270	250.485	1083	223.963	220.005	227.920	66.376	210.540	177.970	255.110
Histidine	1019	83.407	81.871	84.943	24.988	81.920	75.610	88.285	1082	83.219	82.567	83.870	10.927	82.320	76.208	89.225
Isoleucine	1019	52.079	51.211	52.947	14.122	49.460	42.720	57.440	1083	53.359	52.461	54.257	15.062	50.120	44.020	58.545
Lactate	1019	2100.113	2067.921	2132.306	523.693	2014.580	1744.230	2395.610	1083	2119.683	2086.943	2152.422	549.106	2033.660	1719.815	2370.430
Leucine	1019	100.165	98.951	101.378	19.741	97.180	87.050	109.395	1083	102.655	101.437	103.872	20.422	99.420	89.180	112.180
Lysine	1019	118.659	117.261	120.057	22.745	116.500	102.550	131.130	1083	122.724	121.359	124.089	22.893	120.980	108.805	134.355
Methionine	1019	25.187	24.914	25.459	4.430	24.680	22.525	27.075	1083	25.543	25.272	25.815	4.559	25.160	22.965	27.700
N,N-Dimethylglycine	1015	2.202	2.136	2.268	1.075	2.080	1.610	2.570	1080	2.166	2.117	2.215	0.814	2.040	1.580	2.580
Ornithine	1019	61.350	60.448	62.253	14.679	59.890	51.070	70.075	1083	63.782	62.870	64.693	15.285	62.100	52.785	72.640
Phenylalanine	1019	56.086	55.451	56.722	10.333	54.300	49.410	60.805	1083	56.853	56.243	57.463	10.232	55.180	50.270	61.060
Proline	1019	145.382	143.117	147.648	36.852	138.510	121.235	160.215	1083	149.144	146.830	151.458	38.811	142.140	122.435	166.710
Pyruvate	1019	89.855	88.072	91.638	29.009	89.440	70.155	108.120	1083	87.919	86.138	89.700	29.871	87.860	67.270	105.735
Serine	1016	111.122	110.095	112.149	16.685	110.295	99.390	121.168	1082	111.675	110.693	112.657	16.462	110.170	99.922	121.330
Succinate	1019	7.460	7.348	7.571	1.814	7.280	6.270	8.410	1083	7.491	7.387	7.595	1.748	7.290	6.330	8.435
Threonine	1019	178.109	175.263	180.955	46.294	173.400	147.530	206.405	1083	184.338	181.604	187.072	45.858	181.350	151.630	211.065
Tryptophan	1019	49.084	48.561	49.606	8.495	48.580	43.520	54.100	1083	48.909	48.406	49.412	8.430	48.230	43.310	53.435
Tyrosine	1019	59.742	58.872	60.612	14.146	57.910	50.050	66.740	1083	61.670	60.804	62.536	14.524	59.510	52.290	68.030
Uridine	1000	3.906	3.850	3.962	0.899	3.890	3.250	4.470	1071	4.001	3.952	4.050	0.820	3.970	3.440	4.555
Valine	1019	180.871	178.705	183.038	35.244	175.660	155.685	198.790	1083	185.749	183.553	187.944	36.821	178.950	160.790	206.330
2-Aminobutyrate	1019	15.125	14.946	15.305	2.924	14.690	13.055	16.725	1083	15.549	15.369	15.729	3.011	15.080	13.435	17.170
2-Hydroxybutyrate	1019	34.927	34.086	35.769	13.690	32.820	25.675	42.075	1083	35.782	34.932	36.631	14.245	33.120	25.665	42.660
2-Oxoisocaproate	1019	24.808	24.482	25.134	5.299	24.340	20.925	28.225	1083	24.722	24.401	25.042	5.379	23.890	21.070	27.550
3-Hydroxybutyrate	1019	107.367	101.220	113.513	99.988	74.260	39.425	141.080	1082	114.226	107.768	120.684	108.262	74.820	40.160	147.565
3-Hydroxyisobutyrate	1019	10.122	9.927	10.317	3.173	9.440	8.015	11.650	1083	10.380	10.189	10.572	3.214	9.780	8.110	11.955
3-Methyl-2-Oxobutyric Acid	1019	8.674	8.557	8.792	1.918	8.530	7.415	9.865	1083	8.733	8.618	8.847	1.918	8.520	7.405	9.790
3-Methyl-2-Oxovalerate	1019	18.606	18.371	18.841	3.818	18.170	15.900	20.890	1083	18.515	18.284	18.746	3.877	18.000	15.730	20.565

Supplementary Table S2-3 : DateDiff0 female 50's
(4/7)

DateDiff0 female	50 - 54						55 - 59					
Name	N	Mean	95%CI	SD	Median	Quantile [lower, upper]	N	Mean	95%CI	SD	Median	Quantile [lower, upper]
Acetate	1349	35.112	[34.314 , 35.909]	14.928	32.920	[28.390 , 38.740]	2028	35.804	[35.380 , 36.228]	9.729	33.715	[29.040 , 39.770]
Acetone	1352	6.415	[6.122 , 6.709]	5.497	4.645	[2.770 , 8.300]	2030	6.651	[6.413 , 6.890]	5.475	5.020	[3.000 , 8.505]
Alanine	1353	316.488	[312.412 , 320.564]	76.421	305.720	[261.810 , 359.400]	2030	312.062	[308.763 , 315.360]	75.779	301.520	[256.768 , 356.590]
Arginine	1353	48.855	[48.289 , 49.421]	10.611	47.970	[41.510 , 55.030]	2030	49.682	[49.199 , 50.165]	11.095	48.590	[42.390 , 55.468]
Asparagine	1353	57.587	[56.887 , 58.286]	13.116	56.360	[49.400 , 63.910]	2029	56.282	[55.724 , 56.840]	12.817	55.110	[47.990 , 62.090]
Betaine	1353	44.287	[43.675 , 44.898]	11.463	43.050	[36.420 , 49.970]	2030	46.532	[45.993 , 47.072]	12.395	44.640	[38.182 , 52.815]
Carnitine	1348	35.987	[35.586 , 36.388]	7.505	35.685	[30.697 , 40.990]	2027	37.017	[36.674 , 37.359]	7.869	36.560	[31.800 , 41.850]
Citrate	1353	109.357	[108.202 , 110.513]	21.672	106.870	[94.010 , 122.320]	2030	111.376	[110.456 , 112.296]	21.137	109.655	[96.475 , 123.732]
Creatine	1353	39.952	[39.295 , 40.609]	12.326	39.060	[30.890 , 47.740]	2030	41.360	[40.790 , 41.929]	13.090	40.175	[32.162 , 49.078]
Creatinine	1353	53.142	[52.708 , 53.576]	8.131	52.350	[47.750 , 57.750]	2029	53.210	[52.849 , 53.571]	8.286	52.900	[47.450 , 58.000]
Cysteine	1353	53.227	[52.622 , 53.833]	11.355	52.200	[45.400 , 59.640]	2030	55.946	[55.421 , 56.471]	12.062	54.670	[47.930 , 62.720]
Formate	1352	10.715	[10.557 , 10.873]	2.960	10.350	[8.848 , 12.002]	2030	10.942	[10.803 , 11.081]	3.188	10.450	[8.940 , 12.310]
Glucose	1352	4940.295	[4893.831 , 4986.759]	870.895	4858.605	[4476.960 , 5213.892]	2030	5106.167	[5063.715 , 5148.618]	975.292	4938.890	[4595.482 , 5353.085]
Glutamate	1353	46.396	[45.647 , 47.145]	14.045	44.800	[36.210 , 53.690]	2030	48.215	[47.610 , 48.821]	13.910	46.420	[38.073 , 56.677]
Glutamine	1353	475.070	[471.674 , 478.466]	63.680	473.420	[438.080 , 513.400]	2030	483.196	[480.619 , 485.774]	59.212	482.370	[444.078 , 519.830]
Glycerol	1353	72.848	[71.127 , 74.569]	32.274	67.130	[48.590 , 93.200]	2030	73.163	[71.708 , 74.618]	33.432	66.845	[48.713 , 90.948]
Glycine	1353	231.379	[227.834 , 234.923]	66.462	213.450	[188.030 , 256.680]	2030	232.263	[229.256 , 235.270]	69.083	213.830	[184.705 , 262.660]
Histidine	1353	84.855	[84.273 , 85.438]	10.920	83.850	[77.630 , 90.960]	2029	83.604	[83.124 , 84.083]	11.008	82.580	[76.440 , 89.510]
Isoleucine	1353	53.329	[52.547 , 54.111]	14.662	50.580	[43.670 , 59.280]	2030	53.965	[53.311 , 54.619]	15.026	50.715	[44.393 , 59.555]
Lactate	1353	2080.085	[2048.565 , 2111.606]	591.023	1980.640	[1666.920 , 2367.270]	2030	2054.619	[2030.928 , 2078.310]	544.285	1980.895	[1664.392 , 2350.485]
Leucine	1353	103.569	[102.497 , 104.642]	20.101	101.450	[90.380 , 112.910]	2030	105.412	[104.554 , 106.269]	19.702	102.375	[92.058 , 114.587]
Lysine	1353	127.793	[126.769 , 128.817]	19.201	126.780	[115.030 , 138.820]	2030	129.081	[128.189 , 129.973]	20.489	126.755	[115.757 , 139.950]
Methionine	1353	26.234	[26.022 , 26.447]	3.983	25.700	[23.910 , 27.990]	2030	26.157	[25.980 , 26.334]	4.074	25.735	[23.740 , 27.913]
N,N-Dimethylglycine	1344	2.195	[2.153 , 2.237]	0.784	2.110	[1.668 , 2.592]	2019	2.288	[2.249 , 2.328]	0.904	2.190	[1.710 , 2.680]
Ornithine	1353	66.503	[65.739 , 67.268]	14.334	64.920	[56.100 , 75.740]	2030	67.742	[67.136 , 68.348]	13.917	66.335	[57.912 , 76.227]
Phenylalanine	1353	57.285	[56.767 , 57.802]	9.702	55.810	[50.950 , 61.100]	2030	57.930	[57.500 , 58.361]	9.884	56.290	[51.543 , 62.375]
Proline	1353	152.386	[150.188 , 154.585]	41.218	144.120	[125.410 , 167.020]	2030	148.713	[147.137 , 150.289]	36.211	141.070	[123.915 , 164.685]
Pyruvate	1353	83.198	[81.577 , 84.820]	30.398	82.200	[62.060 , 101.690]	2030	81.673	[80.382 , 82.964]	29.660	80.435	[60.487 , 100.870]
Serine	1348	114.241	[113.351 , 115.130]	16.652	112.765	[103.395 , 124.472]	2025	114.986	[114.283 , 115.690]	16.139	113.610	[104.120 , 125.100]
Succinate	1353	7.727	[7.631 , 7.823]	1.802	7.520	[6.560 , 8.610]	2030	7.698	[7.626 , 7.770]	1.656	7.495	[6.510 , 8.648]
Threonine	1353	180.601	[178.389 , 182.812]	41.468	178.550	[151.560 , 208.100]	2030	175.194	[173.416 , 176.972]	40.848	174.495	[147.762 , 200.493]
Tryptophan	1353	49.749	[49.300 , 50.198]	8.419	49.230	[44.010 , 54.660]	2030	49.529	[49.142 , 49.916]	8.898	49.055	[44.103 , 54.218]
Tyrosine	1353	63.040	[62.304 , 63.776]	13.802	60.430	[53.810 , 69.590]	2030	64.463	[63.869 , 65.058]	13.658	62.385	[55.125 , 71.180]
Uridine	1340	4.147	[4.100 , 4.195]	0.886	4.130	[3.530 , 4.730]	2011	4.218	[4.177 , 4.258]	0.927	4.180	[3.560 , 4.800]
Valine	1353	189.056	[187.104 , 191.008]	36.603	184.480	[163.850 , 208.670]	2030	193.024	[191.409 , 194.638]	37.088	187.655	[167.333 , 212.355]
2-Aminobutyrate	1353	15.753	[15.596 , 15.910]	2.951	15.470	[13.750 , 17.330]	2030	16.145	[16.015 , 16.274]	2.976	15.700	[14.110 , 17.705]
2-Hydroxybutyrate	1353	35.451	[34.705 , 36.196]	13.981	32.810	[25.720 , 42.670]	2030	37.225	[36.636 , 37.813]	13.519	35.535	[27.870 , 43.833]
2-Oxoisocaproate	1353	24.247	[23.973 , 24.521]	5.135	23.890	[20.660 , 27.440]	2030	24.336	[24.112 , 24.561]	5.164	23.885	[20.720 , 27.290]
3-Hydroxybutyrate	1352	99.794	[94.878 , 104.710]	92.145	67.965	[37.930 , 129.752]	2030	101.366	[97.461 , 105.271]	89.724	71.975	[39.380 , 133.498]
3-Hydroxyisobutyrate	1353	10.244	[10.075 , 10.413]	3.171	9.740	[8.070 , 11.800]	2030	10.566	[10.437 , 10.694]	2.952	10.160	[8.510 , 12.020]
3-Methyl-2-Oxobutyric Acid	1353	8.412	[8.314 , 8.510]	1.832	8.230	[7.070 , 9.590]	2030	8.490	[8.410 , 8.571]	1.856	8.350	[7.182 , 9.638]
3-Methyl-2-Oxovalerate	1353	18.168	[17.961 , 18.374]	3.872	17.740	[15.390 , 20.420]	2030	18.105	[17.940 , 18.270]	3.792	17.590	[15.390 , 20.230]

Supplementary Table S2-3 : DateDiff0 female 60's
(5/7)

DateDiff0 female	60 - 64						65 - 69					
Name	N	Mean	95%CI	SD	Median	Quantile [lower, upper]	N	Mean	95%CI	SD	Median	Quantile [lower, upper]
Acetate	2891	36.187	[35.829 , 36.544]	9.813	33.920	[29.480 , 40.565]	2948	37.230	[36.836 , 37.624]	10.918	34.755	[30.090 , 41.502]
Acetone	2893	6.709	[6.501 , 6.917]	5.707	4.780	[3.010 , 8.590]	2947	6.951	[6.743 , 7.159]	5.765	5.210	[3.110 , 8.880]
Alanine	2893	316.763	[314.129 , 319.397]	72.256	305.160	[264.490 , 361.180]	2948	318.833	[316.194 , 321.471]	73.057	309.245	[264.732 , 363.198]
Arginine	2891	48.845	[48.452 , 49.237]	10.772	48.150	[41.770 , 54.645]	2948	49.095	[48.719 , 49.470]	10.399	48.390	[42.378 , 55.062]
Asparagine	2891	56.169	[55.737 , 56.601]	11.843	54.890	[48.470 , 62.400]	2948	56.045	[55.621 , 56.469]	11.736	54.650	[48.408 , 62.050]
Betaine	2893	47.409	[46.975 , 47.842]	11.888	45.920	[39.080 , 53.910]	2948	47.859	[47.412 , 48.305]	12.368	46.315	[39.548 , 54.610]
Carnitine	2889	37.740	[37.458 , 38.023]	7.744	37.380	[32.190 , 42.670]	2940	37.694	[37.412 , 37.975]	7.784	37.205	[32.280 , 42.552]
Citrate	2892	113.634	[112.850 , 114.419]	21.517	111.435	[98.505 , 126.325]	2948	115.523	[114.765 , 116.280]	20.973	113.770	[101.170 , 128.232]
Creatine	2893	42.272	[41.800 , 42.744]	12.946	41.230	[33.200 , 50.000]	2948	42.514	[42.042 , 42.986]	13.057	41.940	[33.430 , 50.272]
Creatinine	2891	53.318	[53.004 , 53.632]	8.614	52.520	[47.545 , 58.180]	2948	53.684	[53.362 , 54.005]	8.898	53.005	[48.008 , 58.400]
Cysteine	2893	58.696	[58.230 , 59.161]	12.778	57.450	[50.290 , 65.530]	2948	59.961	[59.510 , 60.411]	12.470	58.725	[51.370 , 67.023]
Formate	2892	11.287	[11.169 , 11.404]	3.227	10.790	[9.260 , 12.670]	2948	11.737	[11.608 , 11.866]	3.573	11.185	[9.600 , 13.060]
Glucose	2892	5139.504	[5107.727 , 5171.280]	871.520	4992.765	[4625.497 , 5421.100]	2947	5194.870	[5161.713 , 5228.026]	917.981	5031.130	[4673.035 , 5460.905]
Glutamate	2893	48.412	[47.920 , 48.904]	13.500	46.430	[38.700 , 56.240]	2948	48.349	[47.870 , 48.827]	13.249	46.610	[38.725 , 55.765]
Glutamine	2893	484.783	[482.683 , 486.883]	57.610	483.630	[446.260 , 520.720]	2948	486.327	[484.296 , 488.358]	56.244	484.675	[449.002 , 522.418]
Glycerol	2893	77.464	[76.240 , 78.688]	33.576	72.890	[52.050 , 97.300]	2948	81.489	[80.234 , 82.744]	34.758	76.310	[55.165 , 101.970]
Glycine	2893	227.747	[225.352 , 230.142]	65.691	209.540	[182.000 , 255.740]	2948	222.458	[220.171 , 224.745]	63.335	205.535	[178.763 , 247.335]
Histidine	2891	82.728	[82.358 , 83.098]	10.153	81.890	[76.020 , 88.375]	2948	82.571	[82.071 , 83.071]	13.844	81.830	[75.798 , 88.002]
Isoleucine	2893	54.492	[53.966 , 55.018]	14.424	51.580	[44.850 , 60.520]	2948	55.707	[55.157 , 56.256]	15.216	52.755	[45.938 , 61.600]
Lactate	2893	2101.552	[2081.683 , 2121.421]	545.026	2002.990	[1710.620 , 2389.020]	2948	2100.079	[2080.632 , 2119.526]	538.507	2011.280	[1715.238 , 2393.760]
Leucine	2893	105.795	[105.084 , 106.506]	19.500	103.380	[92.380 , 115.660]	2948	107.095	[106.368 , 107.821]	20.108	104.395	[93.990 , 116.402]
Lysine	2893	129.853	[129.133 , 130.574]	19.766	128.080	[116.900 , 140.760]	2948	130.102	[129.386 , 130.817]	19.806	128.570	[116.438 , 140.915]
Methionine	2893	26.024	[25.887 , 26.162]	3.772	25.570	[23.620 , 27.820]	2948	26.047	[25.902 , 26.192]	4.007	25.555	[23.540 , 27.840]
N,N-Dimethylglycine	2857	2.334	[2.300 , 2.368]	0.930	2.190	[1.750 , 2.740]	2917	2.355	[2.316 , 2.394]	1.079	2.210	[1.730 , 2.750]
Ornithine	2893	68.382	[67.879 , 68.885]	13.799	66.870	[58.820 , 76.350]	2948	68.867	[68.375 , 69.360]	13.640	67.550	[59.448 , 76.488]
Phenylalanine	2893	59.115	[58.745 , 59.484]	10.128	57.180	[52.520 , 63.580]	2948	60.277	[59.907 , 60.647]	10.244	58.590	[53.667 , 64.910]
Proline	2893	149.918	[148.549 , 151.288]	37.564	142.890	[125.850 , 165.110]	2948	150.533	[149.210 , 151.857]	36.652	143.445	[126.345 , 165.730]
Pyruvate	2893	82.880	[81.842 , 83.919]	28.494	82.370	[63.330 , 100.560]	2948	83.610	[82.568 , 84.652]	28.861	82.705	[64.135 , 101.202]
Serine	2885	114.669	[114.110 , 115.227]	15.299	113.400	[104.450 , 123.990]	2946	114.695	[114.155 , 115.236]	14.961	113.495	[104.672 , 123.578]
Succinate	2893	7.855	[7.794 , 7.916]	1.673	7.650	[6.710 , 8.750]	2948	7.960	[7.898 , 8.022]	1.706	7.750	[6.780 , 8.920]
Threonine	2893	175.365	[173.932 , 176.797]	39.287	175.820	[149.270 , 200.960]	2948	174.237	[172.850 , 175.623]	38.399	173.710	[148.995 , 197.385]
Tryptophan	2893	49.588	[49.274 , 49.901]	8.600	48.910	[43.850 , 54.590]	2948	49.198	[48.890 , 49.507]	8.550	48.545	[43.600 , 53.968]
Tyrosine	2893	65.428	[64.939 , 65.916]	13.397	63.240	[56.750 , 71.520]	2948	66.548	[66.036 , 67.059]	14.175	64.415	[57.140 , 73.220]
Uridine	2846	4.271	[4.236 , 4.305]	0.938	4.250	[3.612 , 4.900]	2912	4.292	[4.258 , 4.326]	0.939	4.290	[3.650 , 4.902]
Valine	2893	194.429	[193.116 , 195.742]	36.022	189.350	[168.700 , 214.390]	2948	197.888	[196.546 , 199.230]	37.165	193.080	[172.427 , 217.257]
2-Aminobutyrate	2893	16.296	[16.187 , 16.404]	2.968	15.880	[14.130 , 17.950]	2948	16.574	[16.465 , 16.684]	3.031	16.180	[14.520 , 18.230]
2-Hydroxybutyrate	2893	38.309	[37.823 , 38.794]	13.307	36.370	[28.750 , 45.700]	2948	39.156	[38.674 , 39.638]	13.342	37.460	[29.670 , 46.545]
2-Oxoisocaproate	2893	24.303	[24.117 , 24.490]	5.117	23.800	[20.640 , 27.290]	2948	24.502	[24.320 , 24.684]	5.046	23.975	[21.105 , 27.422]
3-Hydroxybutyrate	2893	102.744	[99.638 , 105.851]	85.213	74.800	[40.440 , 137.350]	2948	106.073	[102.824 , 109.322]	89.975	80.130	[42.315 , 143.140]
3-Hydroxyisobutyrate	2893	10.895	[10.785 , 11.005]	3.026	10.390	[8.780 , 12.450]	2948	11.268	[11.150 , 11.387]	3.271	10.775	[8.970 , 12.860]
3-Methyl-2-Oxobutyric Acid	2893	8.513	[8.448 , 8.579]	1.793	8.400	[7.210 , 9.650]	2948	8.611	[8.546 , 8.675]	1.792	8.490	[7.320 , 9.713]
3-Methyl-2-Oxovalerate	2893	18.273	[18.136 , 18.411]	3.781	17.800	[15.620 , 20.470]	2948	18.423	[18.288 , 18.559]	3.758	17.900	[15.770 , 20.562]

Supplementary Table S2-3 : DateDiff0 female 70’s
(6/7)

DateDiff0 female	70 - 74						75 - 79					
Name	N	Mean	95%CI	SD	Median	Quantile [lower, upper]	N	Mean	95%CI	SD	Median	Quantile [lower, upper]
Acetate	2316	37.009	[36.546 , 37.473]	11.373	34.415	[29.708 , 40.858]	276	40.915	[39.395 , 42.435]	12.828	38.295	[32.387 , 45.848]
Acetone	2317	6.581	[6.359 , 6.803]	5.449	4.900	[2.920 , 8.350]	276	8.640	[7.855 , 9.425]	6.624	7.030	[3.948 , 12.060]
Alanine	2317	324.951	[321.929 , 327.973]	74.179	314.100	[271.280 , 370.720]	276	321.553	[312.892 , 330.213]	73.086	313.405	[270.005 , 354.830]
Arginine	2317	49.940	[49.527 , 50.352]	10.122	49.110	[43.060 , 55.870]	276	52.849	[51.526 , 54.172]	11.167	51.650	[44.730 , 58.930]
Asparagine	2317	56.784	[56.295 , 57.272]	11.991	55.510	[48.970 , 62.910]	276	56.357	[54.949 , 57.765]	11.882	55.395	[48.957 , 62.862]
Betaine	2317	47.846	[47.318 , 48.373]	12.951	46.190	[38.990 , 54.120]	276	49.395	[47.875 , 50.916]	12.831	47.700	[40.985 , 57.165]
Carnitine	2316	36.916	[36.583 , 37.250]	8.184	36.425	[31.318 , 41.800]	276	39.358	[38.372 , 40.343]	8.316	39.845	[32.725 , 44.898]
Citrate	2317	116.494	[115.613 , 117.376]	21.634	114.770	[100.160 , 129.010]	276	120.617	[117.841 , 123.392]	23.421	118.720	[103.785 , 133.330]
Creatine	2317	42.733	[42.189 , 43.277]	13.358	41.930	[33.520 , 50.960]	276	43.546	[41.815 , 45.277]	14.606	42.140	[33.130 , 52.218]
Creatinine	2316	54.829	[54.435 , 55.223]	9.661	53.695	[48.390 , 59.862]	275	58.579	[56.967 , 60.192]	13.585	57.160	[49.920 , 63.100]
Cysteine	2317	61.891	[61.363 , 62.419]	12.964	60.710	[52.990 , 69.340]	276	63.385	[61.670 , 65.101]	14.477	61.510	[55.230 , 69.833]
Formate	2317	12.120	[11.953 , 12.287]	4.100	11.440	[9.680 , 13.620]	276	12.975	[12.430 , 13.519]	4.597	11.925	[10.082 , 14.742]
Glucose	2316	5276.945	[5234.368 , 5319.521]	1044.873	5070.930	[4676.615 , 5583.358]	275	5356.952	[5224.710 , 5489.193]	1113.947	5098.020	[4773.095 , 5594.905]
Glutamate	2317	49.617	[49.085 , 50.149]	13.053	47.890	[39.840 , 57.250]	276	45.934	[44.507 , 47.362]	12.047	43.895	[37.632 , 52.965]
Glutamine	2317	492.110	[489.728 , 494.493]	58.489	490.340	[452.190 , 528.720]	276	498.023	[491.243 , 504.803]	57.217	496.325	[461.537 , 531.890]
Glycerol	2317	83.104	[81.683 , 84.525]	34.886	78.870	[56.640 , 104.510]	276	95.123	[90.391 , 99.854]	39.929	89.935	[65.172 , 123.160]
Glycine	2317	222.002	[219.396 , 224.608]	63.978	205.110	[177.260 , 249.840]	276	220.460	[213.574 , 227.345]	58.104	204.780	[179.653 , 247.740]
Histidine	2316	81.905	[81.483 , 82.328]	10.365	81.190	[74.888 , 88.438]	276	82.458	[81.130 , 83.786]	11.205	81.420	[75.520 , 88.080]
Isoleucine	2317	56.459	[55.828 , 57.090]	15.484	53.250	[46.460 , 62.720]	276	59.102	[57.291 , 60.912]	15.278	55.425	[49.500 , 65.732]
Lactate	2317	2141.317	[2118.873 , 2163.762]	550.938	2066.710	[1740.080 , 2440.130]	276	1999.748	[1938.299 , 2061.196]	518.562	1924.035	[1642.672 , 2245.162]
Leucine	2317	106.668	[105.837 , 107.499]	20.390	103.750	[93.240 , 116.860]	276	108.568	[106.265 , 110.872]	19.440	106.120	[94.308 , 116.960]
Lysine	2317	131.605	[130.809 , 132.400]	19.532	129.730	[118.030 , 142.550]	276	129.615	[127.329 , 131.902]	19.296	127.530	[116.560 , 140.230]
Methionine	2317	26.149	[25.985 , 26.313]	4.019	25.660	[23.600 , 27.900]	276	25.460	[24.985 , 25.935]	4.005	24.830	[22.855 , 27.282]
N,N-Dimethylglycine	2300	2.435	[2.394 , 2.475]	0.995	2.280	[1.780 , 2.860]	274	2.464	[2.345 , 2.582]	0.994	2.345	[1.843 , 2.940]
Ornithine	2317	70.975	[70.403 , 71.547]	14.042	69.440	[61.250 , 79.160]	276	66.846	[65.216 , 68.476]	13.756	64.925	[56.875 , 73.025]
Phenylalanine	2317	61.984	[61.541 , 62.428]	10.890	59.970	[54.680 , 66.840]	276	63.216	[61.839 , 64.594]	11.623	61.020	[55.803 , 67.772]
Proline	2317	152.649	[151.125 , 154.173]	37.408	145.300	[128.430 , 168.910]	276	154.377	[150.148 , 158.607]	35.694	148.955	[130.433 , 169.620]
Pyruvate	2317	86.664	[85.582 , 87.745]	26.541	85.570	[68.720 , 102.340]	276	66.189	[63.158 , 69.219]	25.574	61.550	[47.430 , 83.455]
Serine	2312	114.512	[113.866 , 115.158]	15.840	113.265	[102.905 , 124.082]	276	117.341	[115.442 , 119.241]	16.029	115.910	[106.040 , 127.200]
Succinate	2317	8.338	[8.265 , 8.411]	1.793	8.120	[7.070 , 9.400]	276	7.738	[7.547 , 7.928]	1.607	7.560	[6.605 , 8.520]
Threonine	2317	170.104	[168.562 , 171.646]	37.857	169.320	[144.500 , 193.440]	276	173.155	[169.243 , 177.067]	33.015	171.440	[149.550 , 195.485]
Tryptophan	2317	49.029	[48.661 , 49.397]	9.030	48.310	[42.850 , 54.230]	276	48.930	[47.900 , 49.960]	8.692	48.430	[43.528 , 53.972]
Tyrosine	2317	68.888	[68.257 , 69.518]	15.482	66.260	[58.220 , 76.410]	276	68.485	[66.704 , 70.267]	15.036	65.800	[58.590 , 75.378]
Uridine	2290	4.234	[4.196 , 4.272]	0.926	4.190	[3.610 , 4.850]	275	4.378	[4.268 , 4.488]	0.927	4.370	[3.735 , 4.945]
Valine	2317	198.782	[197.235 , 200.328]	37.952	194.590	[172.950 , 218.420]	276	203.862	[199.588 , 208.136]	36.068	198.360	[176.042 , 224.158]
2-Aminobutyrate	2317	16.643	[16.517 , 16.769]	3.082	16.270	[14.580 , 18.270]	276	16.986	[16.639 , 17.334]	2.931	16.605	[14.758 , 18.745]
2-Hydroxybutyrate	2317	38.463	[37.921 , 39.004]	13.282	36.720	[28.790 , 46.040]	276	41.134	[39.481 , 42.787]	13.952	39.560	[30.120 , 49.728]
2-Oxoisocaproate	2317	24.001	[23.797 , 24.204]	4.993	23.570	[20.440 , 27.080]	276	25.188	[24.643 , 25.732]	4.594	24.985	[21.945 , 27.792]
3-Hydroxybutyrate	2317	101.383	[97.992 , 104.775]	83.244	74.330	[39.660 , 135.450]	276	118.449	[107.119 , 129.780]	95.618	99.905	[45.423 , 160.067]
3-Hydroxyisobutyrate	2317	11.475	[11.337 , 11.612]	3.367	10.940	[9.150 , 13.140]	276	11.766	[11.395 , 12.136]	3.128	11.205	[9.590 , 13.648]
3-Methyl-2-Oxobutyric Acid	2317	8.530	[8.457 , 8.603]	1.793	8.430	[7.290 , 9.570]	276	8.621	[8.423 , 8.819]	1.671	8.505	[7.305 , 9.652]
3-Methyl-2-Oxovalerate	2317	18.318	[18.164 , 18.472]	3.782	17.800	[15.580 , 20.540]	276	19.168	[18.751 , 19.585]	3.516	18.845	[16.602 , 21.200]

Supplementary Table S2-3 : DateDiff0 female Over 80's (7/7)

DateDiff0 female	over 80											
Name	N	Mean	95%CI				SD	Median	Quantile [lower, upper]			
Acetate	69	47.021	[	40.556 ,	53.487	]	26.914	40.940	[	34.800 ,	48.190	]
Acetone	69	8.376	[	6.785 ,	9.968	]	6.625	6.560	[	3.770 ,	11.660	]
Alanine	69	324.189	[	304.574 ,	343.803	]	81.649	308.820	[	262.060 ,	364.830	]
Arginine	69	52.418	[	50.119 ,	54.717	]	9.569	52.840	[	46.740 ,	57.440	]
Asparagine	69	56.222	[	54.041 ,	58.403	]	9.079	55.910	[	49.950 ,	61.150	]
Betaine	69	49.693	[	46.370 ,	53.017	]	13.835	47.890	[	38.770 ,	56.230	]
Carnitine	69	40.046	[	38.304 ,	41.788	]	7.251	38.990	[	35.320 ,	44.000	]
Citrate	69	121.859	[	116.732 ,	126.985	]	21.341	117.190	[	107.850 ,	136.860	]
Creatine	69	41.560	[	38.227 ,	44.892	]	13.871	42.160	[	30.210 ,	50.880	]
Creatinine	69	61.136	[	57.639 ,	64.633	]	14.557	59.270	[	51.270 ,	65.380	]
Cysteine	69	63.906	[	60.830 ,	66.983	]	12.807	62.960	[	54.160 ,	72.380	]
Formate	68	13.908	[	12.457 ,	15.360	]	5.997	12.530	[	10.717 ,	15.250	]
Glucose	69	5360.525	[	5118.204 ,	5602.845	]	1008.718	5270.570	[	4981.240 ,	5576.820	]
Glutamate	69	47.789	[	44.321 ,	51.257	]	14.436	45.990	[	37.200 ,	54.470	]
Glutamine	69	502.171	[	488.559 ,	515.784	]	56.667	497.030	[	462.860 ,	534.310	]
Glycerol	69	103.283	[	92.966 ,	113.601	]	42.949	96.260	[	72.840 ,	139.170	]
Glycine	69	222.024	[	206.926 ,	237.121	]	62.847	209.020	[	172.530 ,	256.010	]
Histidine	69	81.656	[	79.011 ,	84.301	]	11.009	81.910	[	73.350 ,	88.800	]
Isoleucine	69	60.011	[	56.024 ,	63.998	]	16.598	53.440	[	48.310 ,	69.420	]
Lactate	69	1835.904	[	1729.203 ,	1942.606	]	444.170	1770.980	[	1593.830 ,	2080.430	]
Leucine	69	105.908	[	100.429 ,	111.386	]	22.804	97.840	[	90.210 ,	121.480	]
Lysine	69	126.329	[	122.282 ,	130.375	]	16.844	123.610	[	116.250 ,	135.600	]
Methionine	69	25.238	[	24.315 ,	26.161	]	3.843	25.040	[	22.630 ,	27.070	]
N,N-Dimethylglycine	69	2.460	[	2.262 ,	2.659	]	0.827	2.370	[	1.900 ,	2.930	]
Ornithine	69	66.296	[	62.790 ,	69.802	]	14.594	65.210	[	58.200 ,	74.280	]
Phenylalanine	69	64.822	[	62.012 ,	67.632	]	11.699	61.310	[	56.590 ,	70.610	]
Proline	69	153.766	[	145.434 ,	162.098	]	34.685	146.880	[	132.990 ,	167.940	]
Pyruvate	69	58.941	[	54.404 ,	63.478	]	18.888	54.670	[	47.280 ,	67.260	]
Serine	69	119.866	[	116.725 ,	123.008	]	13.077	117.210	[	113.010 ,	127.890	]
Succinate	69	7.413	[	7.121 ,	7.706	]	1.218	7.240	[	6.560 ,	7.940	]
Threonine	69	173.248	[	166.915 ,	179.580	]	26.361	173.660	[	151.870 ,	188.470	]
Tryptophan	69	47.780	[	45.723 ,	49.838	]	8.563	46.210	[	42.320 ,	52.280	]
Tyrosine	69	69.275	[	65.796 ,	72.754	]	14.483	68.010	[	58.310 ,	75.810	]
Uridine	69	4.269	[	4.047 ,	4.491	]	0.925	4.420	[	3.670 ,	4.840	]
Valine	69	203.949	[	193.929 ,	213.969	]	41.710	195.550	[	170.320 ,	226.630	]
2-Aminobutyrate	69	16.805	[	16.007 ,	17.603	]	3.322	15.880	[	14.330 ,	18.590	]
2-Hydroxybutyrate	69	37.638	[	34.289 ,	40.987	]	13.940	37.860	[	28.020 ,	45.210	]
2-Oxoisocaproate	69	24.583	[	23.281 ,	25.884	]	5.418	23.960	[	21.030 ,	27.900	]
3-Hydroxybutyrate	69	120.476	[	96.549 ,	144.404	]	99.603	82.710	[	49.890 ,	158.440	]
3-Hydroxyisobutyrate	69	11.307	[	10.435 ,	12.178	]	3.627	11.000	[	8.790 ,	12.710	]
3-Methyl-2-Oxobutyric Acid	69	8.578	[	8.072 ,	9.084	]	2.108	8.300	[	7.450 ,	10.120	]
3-Methyl-2-Oxovalerate	69	18.946	[	17.948 ,	19.945	]	4.157	18.440	[	15.750 ,	22.110	]

Supplementary Table S2-4 : DateDiff1 female 20's
(1/7)

DateDiff1 female	20 - 24						25 - 29					
Name	N	Mean	95%CI	SD	Median	Quantile [lower, upper]	N	Mean	95%CI	SD	Median	Quantile [lower, upper]
Acetate	76	27.773	[25.813 , 29.732]	8.576	25.525	[22.817 , 29.915]	135	28.657	[27.282 , 30.032]	8.078	27.160	[24.725 , 30.815]
Acetone	76	4.285	[3.393 , 5.176]	3.903	2.805	[1.922 , 5.442]	135	4.650	[3.927 , 5.373]	4.249	3.220	[2.260 , 5.120]
Alanine	76	323.556	[308.132 , 338.979]	67.495	315.835	[277.735 , 362.350]	135	326.749	[313.454 , 340.043]	78.101	321.480	[265.485 , 368.630]
Arginine	76	47.705	[45.021 , 50.389]	11.745	47.185	[38.803 , 54.952]	135	47.045	[45.350 , 48.740]	9.958	47.030	[40.130 , 54.650]
Asparagine	76	57.109	[54.473 , 59.745]	11.535	56.345	[48.387 , 65.678]	135	58.083	[56.140 , 60.026]	11.414	58.290	[50.060 , 64.960]
Betaine	76	40.074	[37.649 , 42.499]	10.612	39.580	[33.425 , 45.370]	135	42.907	[41.105 , 44.709]	10.586	41.880	[35.670 , 49.470]
Carnitine	73	34.340	[32.294 , 36.386]	8.770	33.670	[27.980 , 40.250]	134	34.617	[33.081 , 36.153]	8.992	33.840	[27.097 , 41.630]
Citrate	76	103.708	[99.481 , 107.935]	18.496	102.995	[92.832 , 114.078]	135	102.316	[98.932 , 105.700]	19.880	102.230	[85.855 , 116.105]
Creatine	76	35.962	[32.984 , 38.940]	13.032	34.300	[27.672 , 40.522]	135	37.515	[35.283 , 39.747]	13.110	35.280	[28.905 , 42.975]
Creatinine	76	50.172	[48.551 , 51.793]	7.094	49.810	[44.550 , 55.560]	135	50.384	[49.122 , 51.646]	7.415	50.990	[45.400 , 55.450]
Cysteine	76	39.706	[37.302 , 42.109]	10.519	38.870	[32.035 , 47.798]	135	42.680	[40.883 , 44.477]	10.557	42.360	[36.155 , 50.145]
Formate	76	11.409	[10.866 , 11.952]	2.378	11.415	[10.058 , 12.747]	135	11.452	[11.027 , 11.877]	2.497	11.180	[10.125 , 12.805]
Glucose	76	3945.461	[3834.091 , 4056.830]	487.372	3926.665	[3655.712 , 4203.418]	135	3977.822	[3867.896 , 4087.749]	645.777	3924.050	[3644.510 , 4272.265]
Glutamate	76	42.044	[39.391 , 44.697]	11.610	39.045	[34.215 , 44.777]	135	43.597	[41.788 , 45.407]	10.628	41.330	[35.575 , 48.550]
Glutamine	76	453.266	[437.451 , 469.082]	69.210	448.135	[401.737 , 504.753]	135	456.284	[445.729 , 466.839]	62.006	451.450	[417.760 , 499.395]
Glycerol	76	72.575	[65.644 , 79.507]	30.333	66.385	[50.967 , 89.950]	135	69.628	[64.505 , 74.752]	30.099	60.510	[47.225 , 90.915]
Glycine	76	201.883	[193.163 , 210.603]	38.161	193.730	[178.185 , 216.898]	135	221.700	[210.740 , 232.661]	64.390	209.210	[181.270 , 244.535]
Histidine	76	82.991	[80.749 , 85.234]	9.814	82.675	[76.798 , 89.190]	135	83.320	[81.738 , 84.903]	9.296	83.030	[76.975 , 88.685]
Isoleucine	76	59.048	[56.103 , 61.993]	12.889	56.290	[49.985 , 66.078]	135	58.127	[55.843 , 60.411]	13.416	55.410	[50.255 , 63.620]
Lactate	76	3191.350	[3072.044 , 3310.655]	522.103	3204.860	[2845.372 , 3508.513]	135	3217.024	[3100.490 , 3333.559]	684.594	3153.270	[2791.755 , 3531.165]
Leucine	76	110.904	[106.788 , 115.021]	18.014	107.680	[96.823 , 123.335]	135	109.142	[106.115 , 112.169]	17.782	108.600	[97.805 , 118.495]
Lysine	76	115.704	[110.973 , 120.435]	20.704	116.555	[100.732 , 128.090]	135	121.032	[117.491 , 124.573]	20.803	119.670	[104.820 , 131.745]
Methionine	76	24.904	[24.099 , 25.710]	3.525	24.305	[22.505 , 26.815]	135	24.892	[24.319 , 25.466]	3.368	24.840	[22.760 , 27.065]
N,N-Dimethylglycine	76	2.425	[2.220 , 2.630]	0.898	2.240	[1.815 , 2.902]	135	2.272	[2.137 , 2.407]	0.793	2.260	[1.630 , 2.765]
Ornithine	76	55.352	[52.596 , 58.107]	12.059	53.475	[45.760 , 63.040]	135	56.800	[54.745 , 58.855]	12.071	56.860	[48.560 , 64.190]
Phenylalanine	76	56.681	[54.638 , 58.724]	8.940	54.290	[51.030 , 62.002]	135	56.829	[54.881 , 58.777]	11.446	54.650	[50.525 , 60.605]
Proline	76	167.688	[160.530 , 174.846]	31.324	163.185	[145.265 , 185.103]	135	166.393	[159.162 , 173.624]	42.481	153.980	[141.450 , 183.635]
Pyruvate	76	43.642	[40.704 , 46.581]	12.860	40.755	[34.575 , 51.045]	135	44.719	[42.602 , 46.836]	12.436	42.510	[35.235 , 51.395]
Serine	76	109.838	[106.081 , 113.595]	16.441	107.485	[96.893 , 118.540]	135	110.264	[107.703 , 112.825]	15.044	109.100	[99.440 , 119.665]
Succinate	76	8.231	[7.878 , 8.585]	1.548	8.220	[7.242 , 9.430]	135	8.105	[7.814 , 8.395]	1.708	7.930	[6.950 , 9.085]
Threonine	76	193.996	[182.777 , 205.215]	49.096	194.750	[154.000 , 224.420]	135	188.806	[181.646 , 195.966]	42.065	182.600	[160.370 , 213.540]
Tryptophan	76	52.101	[50.351 , 53.851]	7.657	52.055	[47.095 , 57.180]	135	50.627	[49.403 , 51.852]	7.193	50.670	[46.210 , 55.645]
Tyrosine	76	58.951	[56.272 , 61.631]	11.726	58.485	[50.323 , 65.468]	135	59.895	[57.894 , 61.897]	11.759	59.290	[52.045 , 67.295]
Uridine	75	4.237	[4.012 , 4.461]	0.975	4.280	[3.675 , 4.760]	133	4.182	[4.016 , 4.347]	0.965	4.190	[3.460 , 4.830]
Valine	76	198.425	[190.410 , 206.441]	35.076	194.310	[175.855 , 214.658]	135	194.745	[188.909 , 200.582]	34.288	191.300	[170.855 , 214.285]
2-Aminobutyrate	76	16.569	[15.880 , 17.258]	3.014	16.175	[14.840 , 18.132]	135	16.305	[15.823 , 16.787]	2.832	16.140	[14.345 , 18.225]
2-Hydroxybutyrate	76	38.038	[34.391 , 41.684]	15.957	32.580	[27.593 , 44.935]	135	34.852	[32.731 , 36.973]	12.460	32.170	[26.055 , 43.805]
2-Oxoisocaproate	76	25.597	[24.394 , 26.801]	5.266	24.970	[21.485 , 29.328]	135	24.539	[23.660 , 25.418]	5.162	23.910	[21.155 , 27.740]
3-Hydroxybutyrate	76	99.552	[76.414 , 122.690]	101.256	52.095	[31.590 , 136.090]	135	91.905	[76.935 , 106.874]	87.942	54.890	[30.255 , 120.350]
3-Hydroxyisobutyrate	76	10.090	[9.267 , 10.912]	3.598	9.175	[8.222 , 10.552]	135	9.420	[8.959 , 9.881]	2.707	8.970	[7.805 , 10.730]
3-Methyl-2-Oxobutyric Acid	76	7.264	[6.848 , 7.680]	1.820	6.800	[6.053 , 8.088]	135	6.969	[6.656 , 7.282]	1.837	6.780	[5.690 , 7.890]
3-Methyl-2-Oxovalerate	76	21.086	[20.171 , 22.002]	4.007	20.805	[17.950 , 23.352]	135	20.028	[19.366 , 20.689]	3.885	19.970	[17.655 , 21.925]

Supplementary Table S2-4 : DateDiff1 female 30's
(2/7)

DateDiff1 female	30 - 34						35 - 39					
Name	N	Mean	95%CI	SD	Median	Quantile [lower, upper]	N	Mean	95%CI	SD	Median	Quantile [lower, upper]
Acetate	252	27.699	[26.971 , 28.426]	5.867	26.840	[23.652 , 30.285]	389	27.678	[27.098 , 28.258]	5.820	27.150	[23.720 , 30.700]
Acetone	252	4.906	[4.383 , 5.428]	4.211	3.605	[2.102 , 6.318]	389	4.844	[4.462 , 5.227]	3.837	3.780	[2.210 , 6.190]
Alanine	252	304.128	[294.664 , 313.591]	76.278	295.525	[245.115 , 351.635]	389	303.104	[296.032 , 310.175]	70.936	293.490	[248.840 , 346.410]
Arginine	252	45.947	[44.556 , 47.337]	11.207	46.230	[38.847 , 53.083]	389	46.429	[45.338 , 47.521]	10.950	45.790	[39.260 , 52.730]
Asparagine	252	57.707	[55.757 , 59.657]	15.716	55.825	[49.015 , 63.330]	389	58.437	[57.058 , 59.817]	13.841	56.590	[49.180 , 64.650]
Betaine	252	41.699	[40.400 , 42.999]	10.473	40.900	[34.382 , 46.960]	389	41.704	[40.567 , 42.842]	11.413	39.710	[34.120 , 47.500]
Carnitine	249	35.215	[34.085 , 36.345]	9.053	34.780	[28.580 , 42.180]	383	34.768	[33.905 , 35.630]	8.586	34.620	[28.410 , 40.945]
Citrate	252	103.951	[101.394 , 106.509]	20.615	102.215	[89.622 , 117.043]	389	105.915	[103.852 , 107.977]	20.687	104.540	[90.940 , 118.720]
Creatine	252	38.123	[36.484 , 39.763]	13.215	36.430	[28.260 , 45.398]	389	37.292	[36.088 , 38.497]	12.083	36.130	[28.150 , 44.500]
Creatinine	252	50.698	[49.838 , 51.558]	6.931	50.055	[45.790 , 55.050]	389	51.063	[50.361 , 51.766]	7.049	50.920	[46.380 , 55.540]
Cysteine	252	43.659	[42.531 , 44.786]	9.090	43.510	[37.198 , 50.360]	389	44.135	[43.084 , 45.186]	10.541	43.980	[36.140 , 50.350]
Formate	252	11.236	[10.827 , 11.646]	3.303	10.865	[9.535 , 12.648]	389	11.429	[11.181 , 11.676]	2.481	11.270	[9.990 , 12.770]
Glucose	252	4052.875	[3958.925 , 4146.826]	757.273	4033.660	[3695.405 , 4274.095]	389	4045.852	[3985.002 , 4106.702]	610.420	3980.000	[3664.920 , 4326.510]
Glutamate	252	43.368	[41.841 , 44.895]	12.309	39.760	[34.942 , 47.685]	389	43.398	[42.184 , 44.613]	12.180	40.960	[35.620 , 47.590]
Glutamine	252	449.911	[441.694 , 458.129]	66.233	451.965	[406.478 , 495.120]	389	454.390	[448.238 , 460.541]	61.709	456.350	[412.120 , 497.110]
Glycerol	252	74.205	[70.154 , 78.256]	32.654	66.770	[50.200 , 90.882]	389	71.132	[68.117 , 74.147]	30.250	66.450	[47.550 , 88.120]
Glycine	252	221.420	[213.696 , 229.144]	62.256	208.665	[179.097 , 259.020]	389	225.672	[219.086 , 232.259]	66.070	212.230	[180.040 , 258.510]
Histidine	252	82.102	[80.564 , 83.640]	12.400	81.560	[75.545 , 86.310]	389	81.995	[81.133 , 82.857]	8.644	81.700	[76.040 , 87.280]
Isoleucine	252	55.324	[53.690 , 56.958]	13.175	53.500	[45.870 , 61.645]	389	54.177	[53.057 , 55.297]	11.231	51.790	[46.190 , 59.620]
Lactate	252	3108.380	[3037.260 , 3179.500]	573.252	3050.040	[2719.038 , 3436.835]	389	3111.059	[3049.146 , 3172.972]	621.084	2989.410	[2669.300 , 3507.810]
Leucine	252	106.295	[104.071 , 108.519]	17.927	104.170	[93.697 , 117.962]	389	105.437	[103.772 , 107.101]	16.695	102.760	[94.490 , 115.020]
Lysine	252	116.216	[113.847 , 118.584]	19.090	116.265	[102.490 , 128.543]	389	117.022	[114.980 , 119.063]	20.484	116.440	[101.230 , 129.250]
Methionine	252	23.899	[23.443 , 24.355]	3.677	23.510	[21.533 , 26.055]	389	23.859	[23.501 , 24.218]	3.601	23.570	[21.600 , 26.000]
N,N-Dimethylglycine	252	2.421	[2.299 , 2.544]	0.989	2.270	[1.790 , 2.832]	388	2.473	[2.338 , 2.607]	1.347	2.285	[1.730 , 2.862]
Ornithine	252	56.802	[55.180 , 58.424]	13.074	55.475	[47.848 , 62.880]	389	56.604	[55.343 , 57.865]	12.649	56.070	[47.760 , 64.080]
Phenylalanine	252	55.985	[54.582 , 57.388]	11.311	54.540	[49.438 , 59.630]	389	55.784	[54.952 , 56.615]	8.343	54.450	[50.430 , 59.580]
Proline	252	159.019	[154.250 , 163.788]	38.440	154.375	[131.767 , 175.340]	389	156.887	[152.930 , 160.843]	39.693	149.670	[129.820 , 172.230]
Pyruvate	252	45.965	[43.990 , 47.940]	15.921	43.730	[36.055 , 53.218]	389	43.863	[42.377 , 45.350]	14.915	41.110	[34.200 , 50.030]
Serine	252	106.679	[104.701 , 108.658]	15.950	106.190	[96.227 , 116.882]	388	107.888	[106.191 , 109.585]	17.002	106.625	[95.122 , 118.390]
Succinate	252	8.113	[7.933 , 8.294]	1.458	7.975	[7.170 , 8.922]	389	8.343	[8.174 , 8.512]	1.693	8.200	[7.210 , 9.240]
Threonine	252	182.733	[177.659 , 187.806]	40.892	178.835	[154.903 , 208.572]	389	182.228	[178.214 , 186.243]	40.270	178.130	[155.160 , 207.860]
Tryptophan	252	50.172	[49.086 , 51.258]	8.753	50.110	[44.080 , 54.995]	389	48.739	[47.939 , 49.539]	8.024	48.340	[43.690 , 53.000]
Tyrosine	252	58.124	[56.612 , 59.637]	12.189	56.685	[50.127 , 63.145]	389	58.344	[57.199 , 59.488]	11.484	56.970	[50.190 , 64.420]
Uridine	251	4.126	[4.016 , 4.237]	0.889	4.070	[3.475 , 4.645]	387	4.149	[4.061 , 4.237]	0.881	4.120	[3.545 , 4.765]
Valine	252	187.411	[183.212 , 191.610]	33.844	181.545	[161.505 , 206.628]	389	183.357	[180.467 , 186.247]	28.987	179.330	[164.590 , 199.350]
2-Aminobutyrate	252	15.761	[15.414 , 16.108]	2.797	15.475	[13.595 , 17.548]	389	15.454	[15.208 , 15.700]	2.464	15.140	[13.720 , 16.900]
2-Hydroxybutyrate	252	37.214	[35.324 , 39.104]	15.233	33.995	[25.695 , 45.100]	389	36.374	[34.918 , 37.829]	14.602	34.170	[25.340 , 45.470]
2-Oxoisocaproate	252	24.398	[23.676 , 25.121]	5.826	23.670	[20.137 , 27.452]	389	23.414	[22.863 , 23.965]	5.528	22.840	[19.840 , 26.520]
3-Hydroxybutyrate	252	116.119	[103.174 , 129.065]	104.343	82.160	[35.925 , 167.800]	389	121.630	[110.027 , 133.232]	116.392	84.650	[39.050 , 165.640]
3-Hydroxyisobutyrate	252	9.512	[9.167 , 9.857]	2.782	9.380	[7.635 , 10.692]	389	9.255	[9.000 , 9.510]	2.559	8.900	[7.410 , 10.690]
3-Methyl-2-Oxobutyric Acid	252	7.182	[6.952 , 7.411]	1.846	7.025	[5.785 , 8.232]	389	6.705	[6.536 , 6.873]	1.693	6.520	[5.420 , 7.720]
3-Methyl-2-Oxovalerate	252	19.606	[19.090 , 20.122]	4.157	19.270	[16.785 , 21.930]	389	18.753	[18.390 , 19.116]	3.644	18.440	[16.130 , 20.840]

Supplementary Table S2-4 : DateDiff1 female 40's
(3/7)

DateDiff1 female	40 - 44						45 - 49					
Name	N	Mean	95%CI	SD	Median	Quantile [lower, upper]	N	Mean	95%CI	SD	Median	Quantile [lower, upper]
Acetate	479	28.609	[26.667 , 30.552]	21.640	26.500	[23.335 , 30.440]	456	27.649	[27.087 , 28.211]	6.104	26.585	[23.758 , 31.008]
Acetone	479	4.605	[4.285 , 4.924]	3.554	3.560	[2.250 , 6.090]	456	5.335	[4.906 , 5.764]	4.663	3.730	[2.398 , 6.872]
Alanine	479	305.377	[298.887 , 311.868]	72.292	293.720	[253.440 , 344.830]	456	306.570	[300.100 , 313.039]	70.299	296.480	[258.578 , 348.185]
Arginine	479	46.832	[45.787 , 47.876]	11.634	47.290	[39.125 , 54.045]	456	45.723	[44.720 , 46.726]	10.900	45.665	[37.852 , 53.105]
Asparagine	479	56.217	[55.053 , 57.382]	12.968	55.650	[47.815 , 63.540]	455	56.681	[55.424 , 57.937]	13.638	55.070	[47.365 , 64.890]
Betaine	479	40.668	[39.692 , 41.645]	10.881	39.200	[33.435 , 47.075]	456	40.835	[39.878 , 41.792]	10.402	39.805	[33.628 , 46.475]
Carnitine	459	35.292	[34.448 , 36.136]	9.200	34.740	[28.595 , 41.630]	436	35.342	[34.577 , 36.106]	8.122	35.610	[29.358 , 40.898]
Citrate	479	105.044	[103.209 , 106.880]	20.448	103.740	[91.275 , 116.245]	456	107.857	[105.917 , 109.796]	21.078	106.795	[93.795 , 119.995]
Creatine	479	36.046	[34.936 , 37.156]	12.365	34.430	[27.465 , 43.600]	456	36.657	[35.452 , 37.862]	13.095	35.375	[27.500 , 44.032]
Creatinine	479	50.670	[50.025 , 51.314]	7.177	50.410	[45.775 , 55.145]	456	51.216	[50.561 , 51.871]	7.116	51.280	[46.120 , 56.160]
Cysteine	479	44.588	[43.633 , 45.542]	10.635	45.010	[36.510 , 52.025]	456	47.100	[46.090 , 48.109]	10.970	46.350	[39.670 , 54.170]
Formate	479	11.897	[11.204 , 12.589]	7.714	11.460	[9.510 , 13.180]	456	10.999	[10.722 , 11.277]	3.015	10.730	[9.162 , 12.422]
Glucose	479	4056.828	[3998.951 , 4114.706]	644.661	4010.980	[3674.950 , 4318.490]	456	4081.002	[4026.891 , 4135.114]	587.983	4032.395	[3676.055 , 4411.905]
Glutamate	479	43.555	[42.524 , 44.587]	11.489	41.230	[35.255 , 48.325]	456	44.516	[43.261 , 45.770]	13.635	41.330	[35.345 , 50.478]
Glutamine	479	445.653	[439.581 , 451.725]	67.634	451.650	[398.570 , 493.325]	456	454.749	[448.460 , 461.038]	68.338	455.910	[409.852 , 502.795]
Glycerol	479	71.036	[68.323 , 73.749]	30.217	65.580	[48.695 , 87.980]	456	80.221	[77.231 , 83.211]	32.486	75.775	[55.995 , 100.240]
Glycine	479	216.359	[211.242 , 221.477]	56.997	206.160	[176.325 , 242.935]	456	221.083	[215.577 , 226.589]	59.829	207.200	[177.140 , 252.208]
Histidine	479	81.616	[80.734 , 82.497]	9.821	80.930	[75.685 , 86.930]	455	82.124	[81.178 , 83.070]	10.271	81.110	[76.165 , 87.065]
Isoleucine	479	54.389	[53.259 , 55.518]	12.577	52.280	[45.790 , 59.860]	456	54.003	[52.794 , 55.213]	13.137	51.620	[45.325 , 59.715]
Lactate	479	3090.036	[3037.350 , 3142.722]	586.832	3056.130	[2687.010 , 3419.410]	456	3020.788	[2965.410 , 3076.167]	601.749	2939.265	[2563.222 , 3388.805]
Leucine	479	104.752	[103.259 , 106.245]	16.633	103.390	[93.400 , 114.250]	456	104.150	[102.464 , 105.836]	18.320	101.980	[91.197 , 114.007]
Lysine	479	115.014	[113.237 , 116.791]	19.792	113.670	[101.840 , 128.070]	456	116.259	[114.359 , 118.159]	20.644	114.695	[102.932 , 128.028]
Methionine	479	24.178	[23.830 , 24.525]	3.874	23.870	[22.010 , 26.020]	456	23.925	[23.627 , 24.223]	3.238	23.655	[21.647 , 25.583]
N,N-Dimethylglycine	478	2.286	[2.199 , 2.374]	0.976	2.120	[1.640 , 2.780]	456	2.416	[1.707 , 3.124]	7.698	1.895	[1.487 , 2.530]
Ornithine	479	56.604	[55.458 , 57.750]	12.765	55.200	[48.060 , 64.825]	456	57.458	[56.301 , 58.615]	12.574	56.550	[49.048 , 65.015]
Phenylalanine	479	56.203	[55.366 , 57.040]	9.327	54.130	[50.145 , 61.320]	456	56.705	[55.841 , 57.569]	9.387	54.895	[50.092 , 60.915]
Proline	479	152.444	[149.496 , 155.392]	32.838	148.020	[129.715 , 166.315]	456	147.570	[144.331 , 150.810]	35.201	139.590	[123.512 , 162.960]
Pyruvate	479	44.534	[42.901 , 46.167]	18.188	42.210	[34.285 , 51.015]	456	43.553	[42.261 , 44.845]	14.040	41.495	[33.320 , 50.640]
Serine	478	104.084	[102.521 , 105.647]	17.394	102.550	[93.570 , 114.615]	456	106.596	[105.085 , 108.108]	16.425	105.530	[95.483 , 118.202]
Succinate	479	8.146	[8.003 , 8.289]	1.595	8.080	[7.040 , 9.010]	456	8.165	[8.003 , 8.326]	1.759	7.970	[6.858 , 9.180]
Threonine	479	179.730	[176.124 , 183.337]	40.167	175.070	[152.195 , 201.480]	456	186.053	[182.025 , 190.082]	43.773	185.035	[154.287 , 213.113]
Tryptophan	479	48.625	[47.919 , 49.331]	7.861	48.430	[43.220 , 53.125]	456	47.773	[47.021 , 48.526]	8.177	47.470	[42.478 , 52.872]
Tyrosine	479	58.591	[57.518 , 59.664]	11.947	57.050	[50.425 , 65.675]	456	58.518	[57.426 , 59.611]	11.871	56.795	[50.570 , 63.935]
Uridine	471	4.314	[4.228 , 4.401]	0.955	4.290	[3.725 , 4.890]	455	4.264	[4.179 , 4.350]	0.932	4.240	[3.605 , 4.865]
Valine	479	186.746	[183.951 , 189.541]	31.133	182.480	[165.550 , 204.365]	456	187.692	[184.655 , 190.728]	32.996	184.755	[165.267 , 205.770]
2-Aminobutyrate	479	15.475	[15.247 , 15.703]	2.540	15.200	[13.760 , 16.980]	456	15.437	[15.181 , 15.694]	2.784	15.135	[13.545 , 17.005]
2-Hydroxybutyrate	479	36.601	[35.420 , 37.783]	13.163	35.140	[27.090 , 44.595]	456	37.102	[35.753 , 38.451]	14.657	34.605	[26.200 , 44.987]
2-Oxoisocaproate	479	23.260	[22.797 , 23.723]	5.158	23.070	[19.790 , 26.465]	456	22.687	[22.197 , 23.176]	5.319	22.010	[18.508 , 26.407]
3-Hydroxybutyrate	479	107.219	[98.800 , 115.638]	93.770	76.650	[39.820 , 141.915]	456	115.026	[105.687 , 124.364]	101.477	79.520	[46.557 , 151.595]
3-Hydroxyisobutyrate	479	9.400	[9.148 , 9.652]	2.802	8.970	[7.580 , 10.855]	456	9.436	[9.154 , 9.717]	3.059	8.855	[7.280 , 10.963]
3-Methyl-2-Oxobutyric Acid	479	6.634	[6.486 , 6.783]	1.653	6.500	[5.480 , 7.690]	456	6.414	[6.259 , 6.568]	1.676	6.160	[5.207 , 7.610]
3-Methyl-2-Oxovalerate	479	18.753	[18.429 , 19.078]	3.611	18.440	[16.210 , 20.760]	456	18.327	[17.972 , 18.682]	3.856	17.980	[15.460 , 20.985]

Supplementary Table S2-4 : DateDiff1 female 50’s
(4/7)

DateDiff1 female	50 - 54						55 - 59					
Name	N	Mean	95%CI	SD	Median	Quantile [lower, upper]	N	Mean	95%CI	SD	Median	Quantile [lower, upper]
Acetate	655	29.724	[29.102 , 30.347]	8.113	27.910	[24.455 , 32.955]	861	31.710	[31.069 , 32.352]	9.597	29.540	[25.630 , 34.770]
Acetone	655	6.540	[6.103 , 6.978]	5.705	4.860	[2.760 , 8.225]	861	6.094	[5.777 , 6.410]	4.728	4.550	[2.790 , 7.820]
Alanine	655	301.297	[296.266 , 306.328]	65.575	296.960	[252.560 , 339.130]	861	308.906	[304.326 , 313.486]	68.471	299.190	[258.310 , 349.490]
Arginine	655	48.722	[47.922 , 49.522]	10.424	48.650	[42.080 , 55.730]	861	49.906	[49.187 , 50.625]	10.751	49.900	[42.930 , 56.590]
Asparagine	655	55.482	[54.536 , 56.428]	12.329	54.710	[47.120 , 62.540]	861	55.534	[54.691 , 56.377]	12.606	53.820	[47.130 , 62.230]
Betaine	655	43.695	[42.836 , 44.553]	11.185	42.410	[35.810 , 49.605]	861	46.999	[46.156 , 47.842]	12.608	45.370	[38.600 , 53.540]
Carnitine	644	37.278	[36.633 , 37.923]	8.332	36.815	[31.875 , 42.733]	858	38.381	[37.837 , 38.926]	8.128	38.095	[32.672 , 43.900]
Citrate	655	111.246	[109.460 , 113.032]	23.280	108.670	[94.890 , 125.775]	861	113.719	[112.229 , 115.210]	22.285	112.650	[98.370 , 127.110]
Creatine	655	39.479	[38.464 , 40.493]	13.224	38.390	[30.185 , 47.920]	861	40.920	[40.062 , 41.779]	12.829	40.030	[32.430 , 48.050]
Creatinine	655	52.369	[51.716 , 53.021]	8.499	51.810	[46.705 , 56.940]	861	53.261	[52.606 , 53.916]	9.786	52.150	[47.760 , 58.000]
Cysteine	655	49.293	[48.421 , 50.164]	11.359	49.020	[42.190 , 55.800]	861	53.037	[52.261 , 53.812]	11.597	52.040	[45.170 , 59.780]
Formate	655	10.989	[10.769 , 11.209]	2.868	10.670	[9.155 , 12.455]	860	11.519	[11.290 , 11.749]	3.425	10.975	[9.410 , 13.010]
Glucose	655	4246.854	[4180.027 , 4313.681]	871.006	4142.110	[3813.050 , 4566.635]	861	4430.984	[4371.916 , 4490.053]	883.079	4311.100	[3938.590 , 4714.570]
Glutamate	655	48.585	[47.382 , 49.788]	15.679	45.590	[38.040 , 55.635]	861	50.208	[49.286 , 51.129]	13.778	47.670	[40.570 , 57.500]
Glutamine	655	470.621	[466.053 , 475.189]	59.536	470.900	[431.355 , 508.410]	861	482.820	[479.021 , 486.619]	56.798	481.880	[446.170 , 519.340]
Glycerol	655	80.235	[77.688 , 82.781]	33.192	75.080	[57.945 , 99.250]	861	79.989	[77.761 , 82.216]	33.306	75.990	[55.050 , 99.330]
Glycine	655	227.622	[222.820 , 232.424]	62.584	213.320	[185.335 , 252.385]	861	233.147	[228.504 , 237.790]	69.415	212.610	[183.780 , 266.170]
Histidine	655	83.020	[81.956 , 84.085]	13.875	81.720	[76.245 , 88.855]	861	82.716	[82.038 , 83.394]	10.133	81.610	[75.810 , 88.240]
Isoleucine	655	55.532	[54.521 , 56.543]	13.176	53.340	[46.365 , 61.295]	861	57.206	[56.251 , 58.161]	14.279	54.710	[47.620 , 64.070]
Lactate	655	2984.742	[2937.425 , 3032.059]	616.715	2893.990	[2597.150 , 3329.795]	861	2954.753	[2914.050 , 2995.456]	608.510	2893.430	[2542.430 , 3307.440]
Leucine	655	107.870	[106.471 , 109.268]	18.222	105.120	[95.500 , 117.155]	861	110.662	[109.355 , 111.969]	19.538	108.870	[97.110 , 121.050]
Lysine	655	120.313	[118.869 , 121.757]	18.821	119.060	[108.020 , 131.680]	861	124.376	[123.169 , 125.582]	18.034	122.250	[112.440 , 134.880]
Methionine	655	24.300	[24.043 , 24.556]	3.341	23.940	[22.120 , 25.975]	861	24.717	[24.481 , 24.954]	3.539	24.360	[22.520 , 26.480]
N,N-Dimethylglycine	655	2.230	[2.128 , 2.332]	1.328	2.120	[1.585 , 2.630]	859	2.276	[2.218 , 2.335]	0.876	2.180	[1.650 , 2.740]
Ornithine	655	60.394	[59.538 , 61.251]	11.161	59.370	[52.465 , 67.390]	861	63.558	[62.737 , 64.378]	12.265	62.070	[55.430 , 69.810]
Phenylalanine	655	57.053	[56.346 , 57.760]	9.214	55.690	[50.965 , 61.300]	861	59.058	[58.328 , 59.787]	10.906	57.330	[52.130 , 63.110]
Proline	655	153.015	[150.104 , 155.926]	37.940	146.250	[127.700 , 171.000]	861	156.506	[153.912 , 159.101]	38.784	147.900	[130.690 , 174.320]
Pyruvate	655	42.756	[41.569 , 43.943]	15.470	41.170	[33.535 , 48.880]	861	43.317	[42.409 , 44.226]	13.576	41.290	[34.090 , 49.950]
Serine	655	110.854	[109.558 , 112.151]	16.902	110.250	[99.795 , 121.085]	859	112.483	[111.340 , 113.625]	17.062	112.180	[101.190 , 123.205]
Succinate	655	8.291	[8.153 , 8.429]	1.796	8.110	[7.155 , 9.260]	861	8.375	[8.268 , 8.482]	1.604	8.190	[7.320 , 9.320]
Threonine	655	182.361	[179.475 , 185.247]	37.612	181.380	[158.245 , 204.805]	861	181.340	[178.759 , 183.921]	38.589	179.320	[154.220 , 206.440]
Tryptophan	655	48.839	[48.195 , 49.483]	8.395	48.550	[43.175 , 54.240]	861	49.915	[49.319 , 50.511]	8.912	49.130	[44.190 , 54.820]
Tyrosine	655	61.311	[60.382 , 62.240]	12.107	59.640	[52.720 , 67.475]	861	63.342	[62.513 , 64.171]	12.393	61.720	[54.770 , 69.370]
Uridine	652	4.551	[4.470 , 4.631]	1.046	4.545	[3.788 , 5.180]	857	4.470	[4.403 , 4.538]	1.005	4.460	[3.780 , 5.160]
Valine	655	194.245	[191.696 , 196.794]	33.223	190.660	[171.350 , 210.015]	861	199.646	[197.325 , 201.967]	34.702	196.030	[175.960 , 218.770]
2-Aminobutyrate	655	16.010	[15.797 , 16.224]	2.789	15.740	[14.100 , 17.445]	861	16.506	[16.313 , 16.699]	2.887	16.260	[14.470 , 18.190]
2-Hydroxybutyrate	655	38.585	[37.351 , 39.820]	16.091	35.980	[27.545 , 46.850]	861	38.897	[37.913 , 39.881]	14.713	36.510	[28.380 , 47.020]
2-Oxoisocaproate	655	22.869	[22.463 , 23.275]	5.293	22.230	[18.975 , 25.900]	861	23.275	[22.926 , 23.623]	5.208	22.950	[19.650 , 26.450]
3-Hydroxybutyrate	655	121.032	[112.274 , 129.791]	114.155	90.650	[44.615 , 160.105]	861	109.392	[103.091 , 115.693]	94.199	77.300	[42.110 , 147.110]
3-Hydroxyisobutyrate	655	9.617	[9.394 , 9.841]	2.917	9.230	[7.730 , 10.955]	861	10.157	[9.948 , 10.366]	3.122	9.690	[7.930 , 11.870]
3-Methyl-2-Oxobutyric Acid	655	6.475	[6.339 , 6.610]	1.765	6.290	[5.175 , 7.635]	861	6.551	[6.438 , 6.664]	1.691	6.420	[5.350 , 7.640]
3-Methyl-2-Oxovalerate	655	18.337	[18.038 , 18.636]	3.899	17.990	[15.530 , 20.500]	861	18.684	[18.419 , 18.950]	3.968	18.400	[15.830 , 20.990]

Supplementary Table S2-4 : DateDiff1 female 60’s
(5/7)

DateDiff1 female	60 - 64						65 - 69					
Name	N	Mean	95%CI	SD	Median	Quantile [lower, upper]	N	Mean	95%CI	SD	Median	Quantile [lower, upper]
Acetate	1103	31.944	[31.416 , 32.472]	8.941	30.070	[26.310 , 35.350]	1072	32.981	[32.401 , 33.562]	9.689	30.865	[26.510 , 36.445]
Acetone	1103	6.118	[5.835 , 6.402]	4.799	4.730	[2.915 , 7.845]	1073	6.295	[5.994 , 6.596]	5.026	4.680	[2.820 , 8.170]
Alanine	1103	314.770	[310.669 , 318.872]	69.421	307.230	[265.180 , 359.325]	1073	319.177	[314.953 , 323.401]	70.518	309.850	[267.980 , 365.020]
Arginine	1103	49.459	[48.821 , 50.098]	10.812	49.210	[42.065 , 56.440]	1072	50.999	[50.338 , 51.661]	11.039	50.115	[43.842 , 57.230]
Asparagine	1103	55.250	[54.554 , 55.946]	11.781	53.630	[47.605 , 61.270]	1072	55.267	[54.570 , 55.965]	11.635	54.225	[47.670 , 61.115]
Betaine	1103	48.159	[47.428 , 48.890]	12.370	46.640	[39.735 , 54.980]	1073	49.161	[48.367 , 49.955]	13.259	47.330	[40.620 , 55.170]
Carnitine	1097	38.932	[38.428 , 39.436]	8.511	38.660	[33.100 , 44.160]	1070	38.860	[38.372 , 39.349]	8.144	38.615	[33.485 , 44.388]
Citrate	1103	114.762	[113.463 , 116.061]	21.987	113.230	[98.795 , 128.350]	1073	117.475	[116.186 , 118.764]	21.520	115.630	[102.320 , 130.590]
Creatine	1103	42.092	[41.333 , 42.851]	12.848	41.170	[32.480 , 50.105]	1073	43.767	[42.965 , 44.570]	13.398	42.600	[34.400 , 51.460]
Creatinine	1103	53.368	[52.881 , 53.856]	8.253	52.720	[47.740 , 57.935]	1073	54.102	[53.506 , 54.698]	9.950	53.060	[48.140 , 58.680]
Cysteine	1103	55.701	[54.973 , 56.430]	12.329	55.050	[47.925 , 62.980]	1073	58.079	[57.287 , 58.871]	13.225	57.410	[49.350 , 65.730]
Formate	1103	11.712	[11.510 , 11.914]	3.419	11.230	[9.645 , 13.105]	1073	12.570	[12.319 , 12.821]	4.190	11.880	[10.060 , 14.080]
Glucose	1103	4572.315	[4516.007 , 4628.623]	953.090	4409.200	[4056.035 , 4826.250]	1073	4670.385	[4607.689 , 4733.082]	1046.658	4479.110	[4112.710 , 4957.630]
Glutamate	1103	51.931	[51.053 , 52.810]	14.871	49.060	[41.255 , 59.610]	1073	52.186	[51.350 , 53.023]	13.961	49.980	[42.060 , 59.830]
Glutamine	1103	488.716	[485.502 , 491.929]	54.391	487.160	[453.325 , 526.600]	1073	490.964	[487.470 , 494.458]	58.327	490.830	[451.360 , 528.090]
Glycerol	1103	84.215	[82.219 , 86.212]	33.795	80.640	[59.665 , 103.760]	1073	88.780	[86.697 , 90.864]	34.779	85.300	[62.360 , 110.000]
Glycine	1103	229.465	[225.411 , 233.520]	68.628	211.820	[181.865 , 256.595]	1073	222.448	[218.696 , 226.199]	62.626	206.820	[178.840 , 246.920]
Histidine	1103	81.425	[80.813 , 82.036]	10.350	80.330	[74.860 , 86.735]	1072	80.987	[80.378 , 81.597]	10.168	80.095	[74.360 , 85.915]
Isoleucine	1103	58.707	[57.833 , 59.581]	14.798	56.200	[48.670 , 65.060]	1073	61.270	[60.277 , 62.263]	16.577	58.480	[50.490 , 67.840]
Lactate	1103	2988.286	[2950.051 , 3026.521]	647.182	2925.810	[2569.580 , 3316.035]	1073	3004.218	[2964.104 , 3044.332]	669.668	2952.140	[2550.750 , 3381.540]
Leucine	1103	112.444	[111.307 , 113.581]	19.244	110.520	[99.680 , 122.480]	1073	115.300	[114.035 , 116.566]	21.126	112.310	[101.910 , 124.270]
Lysine	1103	125.130	[124.040 , 126.220]	18.455	123.420	[112.430 , 135.190]	1073	126.828	[125.648 , 128.007]	19.684	124.340	[113.710 , 137.130]
Methionine	1103	24.548	[24.339 , 24.757]	3.534	24.060	[22.185 , 26.325]	1073	24.698	[24.458 , 24.937]	3.996	24.210	[22.290 , 26.450]
N,N-Dimethylglycine	1099	2.317	[2.266 , 2.369]	0.870	2.190	[1.695 , 2.780]	1067	2.394	[2.336 , 2.452]	0.972	2.220	[1.750 , 2.820]
Ornithine	1103	64.039	[63.318 , 64.761]	12.208	62.210	[55.705 , 70.585]	1073	64.880	[64.097 , 65.664]	13.083	63.530	[56.160 , 71.250]
Phenylalanine	1103	60.338	[59.761 , 60.915]	9.764	58.750	[53.975 , 64.775]	1073	61.740	[61.118 , 62.362]	10.384	59.670	[54.730 , 66.060]
Proline	1103	158.092	[155.711 , 160.474]	40.313	148.830	[133.055 , 174.370]	1073	157.660	[155.545 , 159.775]	35.305	150.770	[132.450 , 175.040]
Pyruvate	1103	43.975	[43.157 , 44.794]	13.851	41.960	[34.485 , 50.540]	1072	45.466	[44.654 , 46.278]	13.553	43.250	[37.030 , 52.208]
Serine	1101	111.084	[110.061 , 112.107]	17.299	110.810	[99.240 , 121.890]	1073	112.602	[111.604 , 113.599]	16.654	111.930	[101.160 , 123.350]
Succinate	1103	8.486	[8.389 , 8.583]	1.644	8.370	[7.430 , 9.395]	1073	8.618	[8.510 , 8.726]	1.804	8.410	[7.480 , 9.450]
Threonine	1103	180.981	[178.719 , 183.244]	38.290	179.680	[155.795 , 204.670]	1073	179.635	[177.469 , 181.801]	36.162	179.090	[155.320 , 202.720]
Tryptophan	1103	50.149	[49.644 , 50.654]	8.549	49.570	[44.805 , 54.655]	1073	49.925	[49.390 , 50.460]	8.931	49.070	[44.170 , 54.680]
Tyrosine	1103	65.849	[65.051 , 66.646]	13.502	63.570	[56.715 , 72.495]	1073	67.355	[66.479 , 68.231]	14.630	64.700	[57.600 , 74.250]
Uridine	1098	4.552	[4.496 , 4.608]	0.938	4.540	[3.890 , 5.128]	1065	4.538	[4.480 , 4.595]	0.960	4.520	[3.900 , 5.190]
Valine	1103	203.321	[201.150 , 205.493]	36.757	199.150	[178.535 , 222.945]	1073	208.221	[205.951 , 210.491]	37.895	202.950	[182.150 , 226.110]
2-Aminobutyrate	1103	16.959	[16.780 , 17.138]	3.028	16.640	[14.915 , 18.590]	1073	17.404	[17.218 , 17.590]	3.102	16.940	[15.290 , 18.940]
2-Hydroxybutyrate	1103	40.585	[39.728 , 41.441]	14.492	38.620	[30.485 , 49.220]	1073	42.382	[41.524 , 43.241]	14.336	40.520	[31.830 , 51.740]
2-Oxoisocaproate	1103	23.540	[23.241 , 23.839]	5.061	23.250	[19.875 , 26.625]	1073	23.791	[23.488 , 24.093]	5.048	23.610	[20.280 , 26.870]
3-Hydroxybutyrate	1103	110.219	[105.209 , 115.229]	84.801	83.790	[42.780 , 151.055]	1073	116.266	[110.805 , 121.727]	91.165	88.920	[47.260 , 154.140]
3-Hydroxyisobutyrate	1103	10.605	[10.415 , 10.795]	3.213	10.110	[8.470 , 12.310]	1073	11.144	[10.946 , 11.341]	3.299	10.490	[8.790 , 13.020]
3-Methyl-2-Oxobutyric Acid	1103	6.730	[6.632 , 6.829]	1.667	6.660	[5.530 , 7.810]	1073	6.848	[6.748 , 6.947]	1.662	6.790	[5.700 , 7.860]
3-Methyl-2-Oxovalerate	1103	18.918	[18.690 , 19.145]	3.851	18.470	[16.065 , 21.340]	1073	19.268	[19.035 , 19.502]	3.894	18.950	[16.550 , 21.600]

Supplementary Table S2-4 : DateDiff1 female 70’s
(6/7)

DateDiff1 female	70 - 74						75 - 79					
Name	N	Mean	95%CI	SD	Median	Quantile [lower, upper]	N	Mean	95%CI	SD	Median	Quantile [lower, upper]
Acetate	577	35.240	[34.173 , 36.307]	13.048	32.410	[27.870 , 38.360]	251	33.932	[32.454 , 35.410]	11.890	31.130	[27.360 , 36.825]
Acetone	577	6.354	[5.968 , 6.739]	4.712	4.950	[3.110 , 8.250]	251	5.985	[5.456 , 6.514]	4.257	4.590	[3.080 , 7.260]
Alanine	577	324.329	[318.377 , 330.282]	72.800	315.620	[269.700 , 370.720]	251	324.470	[315.306 , 333.634]	73.718	318.360	[270.590 , 369.870]
Arginine	577	52.114	[51.162 , 53.065]	11.635	51.390	[44.730 , 58.690]	251	51.418	[49.928 , 52.907]	11.981	50.940	[43.925 , 58.135]
Asparagine	577	57.239	[56.225 , 58.253]	12.398	55.780	[49.070 , 63.930]	251	54.650	[52.866 , 56.435]	14.356	53.780	[45.745 , 60.245]
Betaine	577	49.564	[48.419 , 50.708]	13.998	47.730	[40.590 , 55.960]	251	49.115	[47.498 , 50.732]	13.009	47.740	[41.310 , 54.780]
Carnitine	576	39.145	[38.464 , 39.826]	8.321	39.195	[33.443 , 44.487]	249	39.379	[38.378 , 40.381]	8.022	38.850	[33.930 , 45.010]
Citrate	577	118.069	[116.325 , 119.813]	21.330	116.960	[103.920 , 130.060]	251	118.294	[115.381 , 121.207]	23.431	118.800	[102.625 , 133.140]
Creatine	577	44.413	[43.146 , 45.680]	15.496	42.300	[34.790 , 52.000]	251	44.201	[42.213 , 46.189]	15.994	42.790	[32.255 , 53.855]
Creatinine	577	55.996	[55.060 , 56.933]	11.451	54.670	[49.050 , 60.410]	251	55.203	[53.916 , 56.491]	10.356	53.940	[47.815 , 61.055]
Cysteine	577	59.635	[58.592 , 60.679]	12.761	59.380	[51.890 , 66.410]	251	58.064	[56.608 , 59.520]	11.714	57.860	[51.035 , 64.600]
Formate	576	13.282	[12.882 , 13.682]	4.885	12.295	[10.178 , 15.008]	251	13.335	[12.713 , 13.957]	5.005	12.120	[10.240 , 15.135]
Glucose	577	4730.846	[4635.453 , 4826.240]	1166.666	4515.730	[4122.090 , 5003.840]	250	4751.803	[4627.177 , 4876.430]	1000.499	4548.105	[4166.418 , 5061.255]
Glutamate	577	52.383	[51.327 , 53.438]	12.907	50.380	[43.340 , 59.240]	251	50.544	[48.985 , 52.104]	12.545	48.020	[41.670 , 56.805]
Glutamine	577	499.687	[495.066 , 504.308]	56.512	497.080	[461.340 , 533.370]	251	483.479	[476.075 , 490.883]	59.557	488.510	[440.460 , 520.845]
Glycerol	577	90.208	[87.137 , 93.279]	37.561	84.660	[63.170 , 113.950]	251	92.025	[87.118 , 96.933]	39.477	88.400	[60.355 , 116.680]
Glycine	577	224.716	[219.462 , 229.970]	64.253	208.150	[179.090 , 256.150]	251	226.317	[218.735 , 233.899]	60.992	215.930	[181.915 , 252.605]
Histidine	577	81.082	[80.202 , 81.963]	10.770	79.420	[74.330 , 86.760]	251	78.525	[77.190 , 79.860]	10.739	77.600	[70.980 , 84.410]
Isoleucine	577	62.560	[61.010 , 64.109]	18.949	57.860	[50.800 , 69.280]	251	60.255	[58.181 , 62.329]	16.682	56.690	[49.595 , 69.200]
Lactate	577	2939.099	[2890.337 , 2987.861]	596.363	2859.610	[2524.820 , 3295.890]	251	2904.453	[2833.541 , 2975.366]	570.432	2845.470	[2528.095 , 3236.515]
Leucine	577	114.145	[112.193 , 116.097]	23.874	110.040	[100.440 , 122.670]	251	110.049	[107.474 , 112.623]	20.713	108.850	[97.970 , 119.350]
Lysine	577	128.623	[126.861 , 130.385]	21.553	125.670	[115.040 , 139.060]	251	125.906	[123.409 , 128.403]	20.089	123.160	[112.830 , 137.310]
Methionine	577	24.900	[24.570 , 25.230]	4.034	24.240	[22.150 , 26.410]	251	24.791	[24.290 , 25.293]	4.034	24.180	[22.375 , 26.705]
N,N-Dimethylglycine	575	2.404	[2.333 , 2.474]	0.862	2.270	[1.820 , 2.850]	251	3.069	[1.764 , 4.374]	10.496	2.270	[1.750 , 2.935]
Ornithine	577	67.194	[65.961 , 68.428]	15.087	65.090	[57.700 , 73.440]	251	65.500	[63.881 , 67.119]	13.021	64.170	[56.750 , 72.630]
Phenylalanine	577	63.404	[62.472 , 64.336]	11.399	61.550	[56.050 , 68.000]	251	63.356	[61.979 , 64.733]	11.076	61.410	[55.990 , 70.270]
Proline	577	161.833	[158.408 , 165.258]	41.887	152.630	[135.510 , 177.970]	251	156.812	[152.494 , 161.131]	34.739	149.850	[132.100 , 169.635]
Pyruvate	577	44.173	[43.067 , 45.280]	13.533	42.660	[35.370 , 49.840]	251	43.860	[42.378 , 45.342]	11.922	43.850	[34.820 , 50.895]
Serine	577	112.896	[111.427 , 114.365]	17.969	111.730	[100.500 , 123.660]	250	111.048	[108.924 , 113.172]	17.049	111.035	[100.092 , 121.700]
Succinate	577	8.670	[8.540 , 8.800]	1.594	8.530	[7.550 , 9.550]	251	8.592	[8.365 , 8.819]	1.827	8.490	[7.270 , 9.630]
Threonine	577	175.964	[172.950 , 178.978]	36.860	174.450	[151.330 , 198.760]	251	170.482	[165.256 , 175.708]	42.039	168.540	[145.590 , 190.895]
Tryptophan	577	49.954	[49.193 , 50.716]	9.315	49.060	[43.610 , 54.970]	251	48.753	[47.516 , 49.990]	9.950	47.140	[42.160 , 54.650]
Tyrosine	577	68.890	[67.588 , 70.192]	15.922	65.980	[57.560 , 76.740]	251	69.828	[67.692 , 71.965]	17.187	67.960	[58.365 , 76.980]
Uridine	572	4.522	[4.444 , 4.600]	0.950	4.490	[3.898 , 5.160]	249	4.499	[4.387 , 4.611]	0.897	4.400	[3.850 , 5.110]
Valine	577	209.151	[205.656 , 212.646]	42.745	200.950	[179.680 , 228.900]	251	207.531	[202.547 , 212.516]	40.099	202.370	[180.530 , 229.030]
2-Aminobutyrate	577	17.437	[17.152 , 17.722]	3.485	16.870	[15.080 , 19.010]	251	16.983	[16.583 , 17.384]	3.220	16.730	[14.700 , 18.750]
2-Hydroxybutyrate	577	40.253	[39.109 , 41.398]	14.002	38.130	[30.780 , 48.750]	251	38.394	[36.675 , 40.112]	13.822	37.060	[29.470 , 45.830]
2-Oxoisocaproate	577	23.059	[22.639 , 23.479]	5.140	22.480	[19.900 , 26.190]	251	21.896	[21.285 , 22.507]	4.912	21.690	[18.575 , 24.295]
3-Hydroxybutyrate	577	107.408	[100.946 , 113.871]	79.036	84.750	[43.070 , 153.500]	251	113.379	[100.666 , 126.092]	102.262	81.050	[41.325 , 162.320]
3-Hydroxyisobutyrate	577	11.147	[10.868 , 11.425]	3.408	10.680	[8.990 , 12.800]	251	10.863	[10.491 , 11.236]	2.997	10.590	[8.820 , 12.710]
3-Methyl-2-Oxobutyric Acid	577	6.616	[6.483 , 6.749]	1.626	6.520	[5.600 , 7.690]	251	6.322	[6.127 , 6.516]	1.565	6.380	[5.250 , 7.325]
3-Methyl-2-Oxovalerate	577	19.096	[18.758 , 19.433]	4.130	18.630	[16.110 , 21.480]	251	18.170	[17.690 , 18.650]	3.862	17.730	[15.555 , 20.795]

Supplementary Table S2-4 : DateDiff1 female Over 80's
(7/7)

DateDiff1 female	over 80										
Name	N	Mean	95%CI				SD	Median	Quantile [lower, upper]		
Acetate	79	33.553	[31.749	,	35.357	]	8.055	32.180	[27.515	, 37.310	]
Acetone	79	5.805	[4.948	,	6.663	]	3.829	4.520	[3.115	, 7.745	]
Alanine	79	324.555	[309.535	,	339.575	]	67.056	330.850	[281.475	, 349.630	]
Arginine	79	52.846	[50.470	,	55.221	]	10.606	52.430	[45.465	, 58.875	]
Asparagine	79	56.661	[53.687	,	59.636	]	13.280	54.990	[48.225	, 63.290	]
Betaine	79	49.342	[46.718	,	51.967	]	11.719	49.390	[39.200	, 56.810	]
Carnitine	79	38.265	[36.564	,	39.967	]	7.595	37.380	[33.120	, 43.885	]
Citrate	79	122.185	[117.227	,	127.144	]	22.138	121.020	[105.985	, 138.535	]
Creatine	79	43.267	[40.258	,	46.276	]	13.432	42.100	[33.645	, 51.180	]
Creatinine	79	58.015	[55.324	,	60.705	]	12.013	56.120	[50.320	, 63.415	]
Cysteine	79	57.121	[54.451	,	59.790	]	11.916	55.270	[48.145	, 67.600	]
Formate	79	13.987	[12.868	,	15.105	]	4.993	12.980	[10.770	, 16.435	]
Glucose	79	4780.946	[4554.922	,	5006.969	]	1009.087	4547.020	[4268.435	, 5106.940	]
Glutamate	79	53.334	[50.430	,	56.238	]	12.964	52.200	[44.980	, 59.290	]
Glutamine	79	499.556	[486.686	,	512.427	]	57.460	495.990	[460.825	, 543.325	]
Glycerol	79	86.126	[76.750	,	95.502	]	41.859	79.830	[53.000	, 109.005	]
Glycine	79	214.139	[201.252	,	227.027	]	57.536	198.990	[175.910	, 248.370	]
Histidine	79	77.040	[75.202	,	78.878	]	8.206	77.370	[72.660	, 81.595	]
Isoleucine	79	60.415	[57.250	,	63.580	]	14.131	58.330	[49.640	, 69.085	]
Lactate	79	3069.589	[2910.664	,	3228.515	]	709.528	2969.130	[2558.685	, 3462.410	]
Leucine	79	108.744	[104.571	,	112.916	]	18.629	106.210	[94.010	, 120.645	]
Lysine	79	125.399	[121.299	,	129.499	]	18.303	124.110	[112.485	, 138.575	]
Methionine	79	25.092	[24.221	,	25.963	]	3.889	24.620	[22.375	, 26.780	]
N,N-Dimethylglycine	79	2.785	[2.605	,	2.966	]	0.805	2.720	[2.285	, 3.130	]
Ornithine	79	67.662	[64.441	,	70.883	]	14.381	65.290	[59.740	, 74.475	]
Phenylalanine	79	66.108	[63.589	,	68.626	]	11.243	63.810	[58.580	, 71.315	]
Proline	79	164.718	[156.754	,	172.682	]	35.555	156.050	[140.230	, 187.060	]
Pyruvate	79	46.616	[44.105	,	49.128	]	11.211	44.390	[38.340	, 53.515	]
Serine	79	110.488	[106.549	,	114.428	]	17.587	110.100	[96.825	, 121.005	]
Succinate	79	9.590	[9.180	,	10.000	]	1.831	9.590	[8.340	, 10.830	]
Threonine	79	163.402	[155.054	,	171.749	]	37.270	161.120	[135.550	, 189.020	]
Tryptophan	79	47.935	[45.819	,	50.051	]	9.448	47.280	[42.130	, 53.385	]
Tyrosine	79	71.197	[67.755	,	74.639	]	15.367	67.740	[59.635	, 79.615	]
Uridine	79	4.313	[4.091	,	4.535	]	0.990	4.440	[3.635	, 4.855	]
Valine	79	202.236	[195.001	,	209.471	]	32.302	199.850	[180.585	, 224.235	]
2-Aminobutyrate	79	16.872	[16.295	,	17.448	]	2.573	16.620	[14.935	, 18.685	]
2-Hydroxybutyrate	79	34.564	[32.034	,	37.093	]	11.293	34.140	[26.905	, 41.205	]
2-Oxoisocaproate	79	21.351	[20.247	,	22.455	]	4.931	20.640	[17.850	, 24.495	]
3-Hydroxybutyrate	79	97.997	[79.017	,	116.977	]	84.737	67.180	[34.500	, 139.105	]
3-Hydroxyisobutyrate	79	10.542	[9.853	,	11.232	]	3.079	9.940	[8.415	, 12.260	]
3-Methyl-2-Oxobutyric Acid	79	6.343	[5.997	,	6.688	]	1.543	6.090	[5.425	, 6.880	]
3-Methyl-2-Oxovalerate	79	18.059	[17.243	,	18.875	]	3.644	17.850	[15.640	, 20.065	]

Supplementary Table S2 : These tables are shown in Supplementary Table S2-1 (DateDiff0.male), Supplementary Table S2-2 (DateDiff1.male), Supplementary Table S2-3 (DateDiff0.female), and Supplementary Table S2-4 (DateDiff1.female). Each list of metabolite indices is divided into age groups of 20-24, 25-29, 30-34, 35-39, 40-44, 45-49, 50-54, 55-59, 60-64, 65-69, 70-74, 75-79, and 80 and above, and for each age group, the number (N), mean (Mean), 95% confidence interval (95%CI), standard deviation (SD), median (Median), and quartile range (Quantile [upper, lower]) of the six items are described one line per metabolite.

Supplementary Table S3-1 : male
(1/3)

AgeGroup	20-24 : 25-29		20-24	25-29	25-29 : 30-34		25-29	30-34	30-34 : 35-39		30-34	35-39	35-39 : 40-44		35-39	40-44
Name	p.value	Cliff's delta	Mean	Mean	p.value	Cliff's delta	Mean	Mean	p.value	Cliff's delta	Mean	Mean	p.value	Cliff's delta	Mean	Mean
3-Methyl.2-Oxovalerate	0.000	small	25.362	22.867	1.000	Negl.	22.867	23.001	1.000	Negl.	23.001	22.865	1.000	Negl.	22.865	22.800
2-Oxoisocaproate	0.000	small	34.775	30.878	1.000	Negl.	30.878	31.075	1.000	Negl.	31.075	30.742	1.000	Negl.	30.742	30.906
3-Methyl-2-Oxobutyric.Acid	0.001	small	10.464	9.418	1.000	Negl.	9.418	9.377	1.000	Negl.	9.377	9.490	1.000	Negl.	9.490	9.736
Glutamate	1.000	Negl.	54.751	52.062	1.000	Negl.	52.062	52.960	0.000	Negl.	52.960	56.624	0.000	Negl.	56.624	60.741
Pyruvate	0.376	small	87.326	76.708	1.000	Negl.	76.708	71.403	0.000	Negl.	71.403	79.049	0.000	Negl.	79.049	87.281
Succinate	1.000	Negl.	7.362	6.967	1.000	Negl.	6.967	6.840	0.516	Negl.	6.840	7.004	0.000	Negl.	7.004	7.412
Cysteine	1.000	Negl.	52.453	52.762	1.000	Negl.	52.762	52.875	0.923	Negl.	52.875	54.414	0.000	Negl.	54.414	57.842
Lactate	0.477	small	2695.663	2500.545	0.255	Negl.	2500.545	2394.215	1.000	Negl.	2394.215	2428.743	0.003	Negl.	2428.743	2567.153
Glycerol	0.450	small	54.696	49.956	0.648	Negl.	49.956	53.302	1.000	Negl.	53.302	52.731	0.471	Negl.	52.731	56.151
Citrate	1.000	Negl.	91.733	89.283	1.000	Negl.	89.283	88.324	1.000	Negl.	88.324	87.991	1.000	Negl.	87.991	89.558
Formate	1.000	Negl.	10.120	10.270	1.000	Negl.	10.270	10.025	1.000	Negl.	10.025	10.249	1.000	Negl.	10.249	10.352
Acetate	1.000	Negl.	32.610	33.654	0.997	Negl.	33.654	34.476	1.000	Negl.	34.476	35.698	1.000	Negl.	35.698	33.461
Glucose	1.000	Negl.	4626.419	4750.731	1.000	Negl.	4750.731	4793.079	0.418	Negl.	4793.079	4876.737	1.000	Negl.	4876.737	5006.300
Threonine	1.000	Negl.	193.539	192.584	1.000	Negl.	192.584	189.706	1.000	Negl.	189.706	186.906	1.000	Negl.	186.906	186.009
Creatinine	1.000	Negl.	67.015	66.787	1.000	Negl.	66.787	66.482	1.000	Negl.	66.482	65.898	0.569	Negl.	65.898	67.068
Phenylalanine	1.000	Negl.	63.541	61.169	1.000	Negl.	61.169	61.123	1.000	Negl.	61.123	60.768	1.000	Negl.	60.768	61.015
3-Hydroxybutyrate	1.000	Negl.	57.689	50.859	0.936	Negl.	50.859	52.794	1.000	Negl.	52.794	58.145	1.000	Negl.	58.145	60.220
Serine	1.000	Negl.	112.537	111.262	0.729	Negl.	111.262	109.140	1.000	Negl.	109.140	109.562	1.000	Negl.	109.562	109.579
Betaine	1.000	Negl.	48.653	49.150	1.000	Negl.	49.150	49.888	1.000	Negl.	49.888	49.849	1.000	Negl.	49.849	49.053
Tryptophan	1.000	Negl.	59.540	59.002	1.000	Negl.	59.002	58.482	1.000	Negl.	58.482	57.670	0.699	Negl.	57.670	56.650
Carnitine	1.000	Negl.	39.495	39.056	1.000	Negl.	39.056	38.953	1.000	Negl.	38.953	38.965	1.000	Negl.	38.965	38.782
Ornithine	1.000	Negl.	72.606	70.551	1.000	Negl.	70.551	69.322	1.000	Negl.	69.322	70.771	1.000	Negl.	70.771	72.221

Supplementary Table S3-1 : male
(2/3)

AgeGroup	40-44 : 45-49		40-44	45-49	45-49 : 50-54		45-49	50-54	50-54 : 55-59		50-54	55-59	55-59 : 60-64		55-59	60-64
Name	p.value	Cliff's delta	Mean	Mean	p.value	Cliff's delta	Mean	Mean	p.value	Cliff's delta	Mean	Mean	p.value	Cliff's delta	Mean	Mean
3-Methyl-2-Oxovalerate	1.000	Negl.	22.800	23.055	1.000	Negl.	23.055	22.987	1.000	Negl.	22.987	22.607	1.000	Negl.	22.607	22.631
2-Oxoisocaproate	1.000	Negl.	30.906	31.175	1.000	Negl.	31.175	31.139	1.000	Negl.	31.139	30.672	1.000	Negl.	30.672	30.598
3-Methyl-2-Oxobutyric.Acid	1.000	Negl.	9.736	9.896	1.000	Negl.	9.896	9.896	1.000	Negl.	9.896	9.668	1.000	Negl.	9.668	9.675
Glutamate	1.000	Negl.	60.741	62.909	1.000	Negl.	62.909	63.707	1.000	Negl.	63.707	63.163	1.000	Negl.	63.163	61.53
Pyruvate	0.000	Negl.	87.281	94.154	1.000	Negl.	94.154	94.998	0.775	Negl.	94.998	91.248	1.000	Negl.	91.248	88.638
Succinate	0.004	Negl.	7.412	7.772	1.000	Negl.	7.772	7.802	1.000	Negl.	7.802	7.845	1.000	Negl.	7.845	7.997
Cysteine	0.003	Negl.	57.842	60.373	1.000	Negl.	60.373	61.915	1.000	Negl.	61.915	62.614	0.000	Negl.	62.614	65.266
Lactate	0.941	Negl.	2567.153	2655.692	1.000	Negl.	2655.692	2664.209	1.000	Negl.	2664.209	2600.19	0.726	Negl.	2600.19	2540.873
Glycerol	0.023	Negl.	56.151	60.076	1.000	Negl.	60.076	62.068	1.000	Negl.	62.068	61.331	0.042	Negl.	61.331	64.64
Citrate	1.000	Negl.	89.558	91.980	1.000	Negl.	91.980	92.426	1.000	Negl.	92.426	93.219	0.000	small	93.219	98.67
Formate	0.988	Negl.	10.352	10.671	0.228	Negl.	10.671	11.305	1.000	Negl.	11.305	11.253	0.006	Negl.	11.253	11.734
Acetate	1.000	Negl.	33.461	33.670	1.000	Negl.	33.670	36.675	1.000	Negl.	36.675	36.21	0.000	Negl.	36.21	36.258
Glucose	0.496	Negl.	5006.300	5171.741	0.033	Negl.	5171.741	5371.064	1.000	Negl.	5371.064	5451.282	0.016	Negl.	5451.282	5556.627
Threonine	1.000	Negl.	186.009	188.744	1.000	Negl.	188.744	187.217	1.000	Negl.	187.217	184.495	0.024	Negl.	184.495	188.881
Creatinine	1.000	Negl.	67.068	67.293	1.000	Negl.	67.293	66.895	1.000	Negl.	66.895	66.831	0.001	Negl.	66.831	68.835
Phenylalanine	1.000	Negl.	61.015	61.279	1.000	Negl.	61.279	62.354	1.000	Negl.	62.354	62.984	0.000	Negl.	62.984	64.889
3-Hydroxybutyrate	1.000	Negl.	60.220	70.419	1.000	Negl.	70.419	73.479	1.000	Negl.	73.479	70.219	0.011	Negl.	70.219	79.653
Serine	1.000	Negl.	109.579	110.761	1.000	Negl.	110.761	111.929	1.000	Negl.	111.929	111.964	0.014	Negl.	111.964	114.318
Betaine	1.000	Negl.	49.053	48.496	1.000	Negl.	48.496	50.684	0.030	Negl.	50.684	52.477	0.210	Negl.	52.477	53.966
Tryptophan	1.000	Negl.	56.650	56.778	1.000	Negl.	56.778	56.152	1.000	Negl.	56.152	56.339	1.000	Negl.	56.339	56.079
Carnitine	1.000	Negl.	38.782	38.363	1.000	Negl.	38.363	38.684	1.000	Negl.	38.684	39.234	0.050	Negl.	39.234	40.334
Ornithine	1.000	Negl.	72.221	74.144	1.000	Negl.	74.144	74.193	1.000	Negl.	74.193	74.571	1.000	Negl.	74.571	74.445

Supplementary Table S3-1 : male
(3/3)

AgeGroup	60-64 : 65-69		60-64	65-69	65-69 : 70-74		65-69	70-74	70-74 : 75-79		70-74	75-79	75-79 : 80over		75-79	80over
Name	p.value	Cliff's delta	Mean	Mean	p.value	Cliff's delta	Mean	Mean	p.value	Cliff's delta	Mean	Mean	p.value	Cliff's delta	Mean	Mean
3-Methyl-2-Oxovalerate	1.000	Negl.	22.631	22.467	1.000	Negl.	22.467	22.194	1.000	Negl.	22.194	21.91	1.000	Negl.	21.91	22.657
2-Oxoisocaproate	1.000	Negl.	30.598	30.409	0.157	Negl.	30.409	29.846	1.000	Negl.	29.846	29.326	1.000	Negl.	29.326	29.663
3-Methyl-2-Oxobutyric.Acid	1.000	Negl.	9.675	9.662	1.000	Negl.	9.662	9.591	1.000	Negl.	9.591	9.324	1.000	Negl.	9.324	9.519
Glutamate	0.695	Negl.	61.53	60.169	0.000	Negl.	60.169	58.038	0.001	small	58.038	53.908	0.793	small	53.908	49.363
Pyruvate	1.000	Negl.	88.638	88.908	1.000	Negl.	88.908	89.95	0.000	medium	89.95	72.146	0.000	medium	72.146	54.118
Succinate	0.007	Negl.	7.997	8.210	0.000	Negl.	8.210	8.494	0.025	Negl.	8.494	8.098	1.000	small	8.098	7.599
Cysteine	0.003	Negl.	65.266	66.971	1.000	Negl.	66.971	68.241	1.000	Negl.	68.241	65.572	1.000	Negl.	65.572	63.323
Lactate	1.000	Negl.	2540.873	2524.743	0.831	Negl.	2524.743	2469.845	0.000	small	2469.845	2231.89	1.000	small	2231.89	2092.501
Glycerol	0.933	Negl.	64.64	66.591	0.001	Negl.	66.591	69.959	1.000	Negl.	69.959	74.745	0.003	medium	74.745	92.412
Citrate	0.000	Negl.	98.67	101.808	0.000	Negl.	101.808	106.212	1.000	Negl.	106.212	108.809	0.011	small	108.809	119.568
Formate	0.923	Negl.	11.734	11.933	0.000	Negl.	11.933	12.631	1.000	Negl.	12.631	12.512	1.000	Negl.	12.512	13.476
Acetate	1.000	Negl.	36.258	36.829	1.000	Negl.	36.829	37.238	0.000	small	37.238	40.349	0.071	small	40.349	47.207
Glucose	1.000	Negl.	5556.627	5538.297	1.000	Negl.	5538.297	5552.293	1.000	Negl.	5552.293	5693.861	1.000	Negl.	5693.861	5358.394
Threonine	0.239	Negl.	188.881	185.270	0.003	Negl.	185.270	180.742	1.000	Negl.	180.742	182.766	1.000	Negl.	182.766	185.922
Creatinine	1.000	Negl.	68.835	70.030	0.090	Negl.	70.030	71.233	0.079	Negl.	71.233	73.8	1.000	Negl.	73.8	76.17
Phenylalanine	1.000	Negl.	64.889	65.665	0.087	Negl.	65.665	66.467	1.000	Negl.	66.467	67.23	1.000	Negl.	67.23	66.505
3-Hydroxybutyrate	1.000	Negl.	79.653	84.190	1.000	Negl.	84.190	90.17	1.000	Negl.	90.17	99.34	0.270	small	99.34	136.492
Serine	1.000	Negl.	114.318	113.276	1.000	Negl.	113.276	113.311	1.000	Negl.	113.311	115.806	1.000	small	115.806	121.247
Betaine	1.000	Negl.	53.966	54.271	0.000	Negl.	54.271	56.321	1.000	Negl.	56.321	55.628	1.000	small	55.628	58.286
Tryptophan	1.000	Negl.	56.079	55.620	0.004	Negl.	55.620	54.554	1.000	Negl.	54.554	53.574	1.000	Negl.	53.574	52.24
Carnitine	1.000	Negl.	40.334	39.848	0.818	Negl.	39.848	39.195	0.025	Negl.	39.195	40.891	1.000	small	40.891	43.923
Ornithine	1.000	Negl.	74.445	75.223	1.000	Negl.	75.223	75.929	0.029	Negl.	75.929	72.803	1.000	Negl.	72.803	70.148

Supplementary Table S3-2 : female (1/3)

AgeGroup	20-24 : 25-29		20 - 24	25 - 29	25-29 : 30-34		25 - 29	30 - 34	30-34 : 35-39		30 - 34	35 - 39	35-39 : 40-44		35 - 39	40 - 44
Name	p.value	Cliff's delta	Mean	Mean	p.value	Cliff's delta	Mean	Mean	p.value	Cliff's delta	Mean	Mean	p.value	Cliff's delta	Mean	Mean
2-Oxoisocaproate	1.000	Negl.	27.695	26.632	0.006	Negl.	26.632	25.210	0.275	Negl.	25.210	24.463	1.000	Negl.	24.463	24.808
3-Methyl-2-Oxovalerate	1.000	Negl.	20.985	20.020	0.012	Negl.	20.020	18.963	0.008	Negl.	18.963	18.306	1.000	Negl.	18.306	18.606
Tryptophan	1.000	Negl.	52.692	51.123	1.000	Negl.	51.123	50.498	0.018	Negl.	50.498	49.095	1.000	Negl.	49.095	49.084
Leucine	1.000	Negl.	105.207	104.271	1.000	Negl.	104.271	101.905	0.010	Negl.	101.905	99.333	1.000	Negl.	99.333	100.165
2-Aminobutyrate	1.000	Negl.	16.061	15.876	1.000	Negl.	15.876	15.511	0.004	Negl.	15.511	15.032	1.000	Negl.	15.032	15.125
Valine	1.000	Negl.	194.210	190.592	1.000	Negl.	190.592	186.763	0.001	Negl.	186.763	179.860	1.000	Negl.	179.860	180.871
Isoleucine	1.000	Negl.	56.525	54.964	1.000	Negl.	54.964	53.452	0.000	Negl.	53.452	51.082	1.000	Negl.	51.082	52.079
Acetone	1.000	Negl.	4.808	5.290	1.000	Negl.	5.290	4.958	1.000	Negl.	4.958	5.226	0.042	Negl.	5.226	5.597
Lactate	1.000	Negl.	2202.081	2211.678	1.000	Negl.	2211.678	2184.782	1.000	Negl.	2184.782	2214.286	0.000	Negl.	2214.286	2100.113
Pyruvate	1.000	Negl.	88.846	95.760	1.000	Negl.	95.760	96.338	1.000	Negl.	96.338	98.361	0.000	small	98.361	89.855
Glycerol	1.000	Negl.	68.700	69.754	1.000	Negl.	69.754	68.529	1.000	Negl.	68.529	66.889	0.001	Negl.	66.889	72.025
Glucose	1.000	Negl.	4423.598	4511.178	1.000	Negl.	4511.178	4568.271	1.000	Negl.	4568.271	4581.241	0.000	small	4581.241	4735.030
Cysteine	1.000	Negl.	44.666	45.974	1.000	Negl.	45.974	47.402	1.000	Negl.	47.402	47.876	1.000	Negl.	47.876	48.450
Glutamine	1.000	Negl.	433.682	433.862	1.000	Negl.	433.862	441.519	1.000	Negl.	441.519	437.998	1.000	Negl.	437.998	443.658
Ornithine	1.000	Negl.	59.701	61.980	1.000	Negl.	61.980	62.343	1.000	Negl.	62.343	62.806	1.000	Negl.	62.806	61.350
Lysine	1.000	Negl.	119.340	121.441	1.000	Negl.	121.441	122.463	0.311	Negl.	122.463	119.657	1.000	Negl.	119.657	118.659
Tyrosine	1.000	Negl.	60.716	60.668	1.000	Negl.	60.668	60.016	1.000	Negl.	60.016	59.399	1.000	Negl.	59.399	59.742
Glutamate	0.616	small	38.877	41.738	1.000	Negl.	41.738	40.993	1.000	Negl.	40.993	42.125	1.000	Negl.	42.125	41.791
Citrate	1.000	Negl.	99.967	99.818	1.000	Negl.	99.818	101.654	1.000	Negl.	101.654	102.127	1.000	Negl.	102.127	104.174
Betaine	1.000	Negl.	41.208	40.300	1.000	Negl.	40.300	41.926	1.000	Negl.	41.926	40.817	1.000	Negl.	40.817	40.738
Histidine	1.000	Negl.	82.484	82.725	1.000	Negl.	82.725	82.822	1.000	Negl.	82.822	82.197	1.000	Negl.	82.197	83.407
Carnitine	1.000	Negl.	34.328	34.071	1.000	Negl.	34.071	33.596	1.000	Negl.	33.596	32.989	1.000	Negl.	32.989	33.746
Succinate	1.000	Negl.	7.058	7.156	1.000	Negl.	7.156	7.446	1.000	Negl.	7.446	7.624	1.000	Negl.	7.624	7.460
Acetate	1.000	Negl.	30.578	32.157	1.000	Negl.	32.157	32.001	1.000	Negl.	32.001	31.666	0.363	Negl.	31.666	33.021
Arginine	1.000	Negl.	44.227	44.435	1.000	Negl.	44.435	44.178	1.000	Negl.	44.178	43.342	0.882	Negl.	43.342	44.785
Creatine	1.000	Negl.	33.121	35.645	0.133	Negl.	35.645	38.170	0.069	Negl.	38.170	36.284	1.000	Negl.	36.284	36.474
Methionine	1.000	Negl.	26.124	25.990	1.000	Negl.	25.990	25.714	0.447	Negl.	25.714	25.171	1.000	Negl.	25.171	25.187
Serine	1.000	Negl.	111.812	112.735	1.000	Negl.	112.735	113.363	0.058	Negl.	113.363	110.917	1.000	Negl.	110.917	111.122
Uridine	1.000	Negl.	3.703	3.870	1.000	Negl.	3.870	3.941	1.000	Negl.	3.941	3.906	1.000	Negl.	3.906	3.906
3-Methyl-2-Oxobutyric.Acid	1.000	Negl.	9.262	9.148	0.525	Negl.	9.148	8.840	1.000	Negl.	8.840	8.611	1.000	Negl.	8.611	8.674
2-Hydroxybutyrate	1.000	Negl.	33.835	34.180	1.000	Negl.	34.180	32.912	0.317	Negl.	32.912	34.000	1.000	Negl.	34.000	34.927
Asparagine	1.000	Negl.	58.062	57.214	1.000	Negl.	57.214	58.014	1.000	Negl.	58.014	58.153	1.000	Negl.	58.153	58.770
Threonine	1.000	Negl.	180.002	182.272	1.000	Negl.	182.272	175.246	1.000	Negl.	175.246	174.919	1.000	Negl.	174.919	178.109
3-Hydroxyisobutyrate	1.000	Negl.	10.665	10.417	1.000	Negl.	10.417	10.068	1.000	Negl.	10.068	10.031	1.000	Negl.	10.031	10.122
Formate	1.000	Negl.	10.896	11.301	1.000	Negl.	11.301	10.760	1.000	Negl.	10.760	10.758	1.000	Negl.	10.758	10.748
Phenylalanine	1.000	Negl.	56.809	56.484	1.000	Negl.	56.484	55.988	1.000	Negl.	55.988	54.856	0.862	Negl.	54.856	56.086
Creatinine	1.000	Negl.	50.659	49.652	1.000	Negl.	49.652	50.537	1.000	Negl.	50.537	51.135	1.000	Negl.	51.135	51.736

Supplementary Table S3-2 : female (2/3)

AgeGroup	40-44 : 45-49		40 - 44	45 - 49	45-49 : 50-54		45 - 49	50 - 54	50-54 : 55-59		50 - 54	55 - 59	55-59 : 60-64		55 - 59	60 - 64
Name	p.value	Cliff's delta	Mean	Mean	p.value	Cliff's delta	Mean	Mean	p.value	Cliff's delta	Mean	Mean	p.value	Cliff's delta	Mean	Mean
2-Oxoisocaproate	1.000	Negl.	24.808	24.722	1.000	Negl.	24.722	24.247	1.000	Negl.	24.247	24.336	1.000	Negl.	24.336	24.303
3-Methyl-2-Oxovalerate	1.000	Negl.	18.606	18.515	1.000	Negl.	18.515	18.168	1.000	Negl.	18.168	18.105	1.000	Negl.	18.105	18.273
Tryptophan	1.000	Negl.	49.084	48.909	0.893	Negl.	48.909	49.749	1.000	Negl.	49.749	49.529	1.000	Negl.	49.529	49.588
Leucine	0.167	Negl.	100.165	102.655	1.000	Negl.	102.655	103.569	0.573	Negl.	103.569	105.412	1.000	Negl.	105.412	105.795
2-Aminobutyrate	0.057	Negl.	15.125	15.549	1.000	Negl.	15.549	15.753	0.041	Negl.	15.753	16.145	1.000	Negl.	16.145	16.296
Valine	0.106	Negl.	180.871	185.749	0.280	Negl.	185.749	189.056	0.294	Negl.	189.056	193.024	1.000	Negl.	193.024	194.429
Isoleucine	1.000	Negl.	52.079	53.359	1.000	Negl.	53.359	53.329	1.000	Negl.	53.329	53.965	1.000	Negl.	53.965	54.492
Acetone	0.626	Negl.	5.597	6.267	1.000	Negl.	6.267	6.415	1.000	Negl.	6.415	6.651	1.000	Negl.	6.651	6.709
Lactate	1.000	Negl.	2100.113	2119.683	1.000	Negl.	2119.683	2080.085	1.000	Negl.	2080.085	2054.619	0.200	Negl.	2054.619	2101.552
Pyruvate	1.000	Negl.	89.855	87.919	0.007	Negl.	87.919	83.198	1.000	Negl.	83.198	81.673	1.000	Negl.	81.673	82.880
Glycerol	1.000	Negl.	72.025	74.931	1.000	Negl.	74.931	72.848	1.000	Negl.	72.848	73.163	0.000	Negl.	73.163	77.464
Glucose	0.000	Negl.	4735.030	4881.395	0.005	Negl.	4881.395	4940.295	0.000	Negl.	4940.295	5106.167	0.227	Negl.	5106.167	5139.504
Cysteine	0.000	Negl.	48.450	51.090	0.000	Negl.	51.090	53.227	0.000	Negl.	53.227	55.946	0.000	Negl.	55.946	58.696
Glutamine	0.000	Negl.	443.658	459.383	0.000	Negl.	459.383	475.070	0.025	Negl.	475.070	483.196	1.000	Negl.	483.196	484.783
Ornithine	0.036	Negl.	61.350	63.782	0.000	Negl.	63.782	66.503	0.909	Negl.	66.503	67.742	1.000	Negl.	67.742	68.382
Lysine	0.000	Negl.	118.659	122.724	0.000	small	122.724	127.793	1.000	Negl.	127.793	129.081	1.000	Negl.	129.081	129.853
Tyrosine	0.029	Negl.	59.742	61.670	0.562	Negl.	61.670	63.040	0.021	Negl.	63.040	64.463	0.262	Negl.	64.463	65.428
Glutamate	0.000	Negl.	41.791	44.799	0.080	Negl.	44.799	46.396	0.004	Negl.	46.396	48.215	1.000	Negl.	48.215	48.412
Citrate	0.043	Negl.	104.174	107.407	1.000	Negl.	107.407	109.357	0.200	Negl.	109.357	111.376	0.054	Negl.	111.376	113.634
Betaine	1.000	Negl.	40.738	41.093	0.000	small	41.093	44.287	0.000	Negl.	44.287	46.532	0.108	Negl.	46.532	47.409
Histidine	1.000	Negl.	83.407	83.219	0.019	Negl.	83.219	84.855	0.046	Negl.	84.855	83.604	0.891	Negl.	83.604	82.728
Carnitine	0.181	Negl.	33.746	34.688	0.001	Negl.	34.688	35.987	0.049	Negl.	35.987	37.017	0.060	Negl.	37.017	37.740
Succinate	1.000	Negl.	7.460	7.491	0.037	Negl.	7.491	7.727	1.000	Negl.	7.727	7.698	0.092	Negl.	7.698	7.855
Acetate	1.000	Negl.	33.021	32.744	0.000	Negl.	32.744	35.112	0.065	Negl.	35.112	35.804	1.000	Negl.	35.804	36.187
Arginine	1.000	Negl.	44.785	45.598	0.000	small	45.598	48.855	1.000	Negl.	48.855	49.682	1.000	Negl.	49.682	48.845
Creatine	0.253	Negl.	36.474	37.805	0.000	Negl.	37.805	39.952	0.411	Negl.	39.952	41.360	0.434	Negl.	41.360	42.272
Methionine	0.461	Negl.	25.187	25.543	0.000	Negl.	25.543	26.234	1.000	Negl.	26.234	26.157	1.000	Negl.	26.157	26.024
Serine	1.000	Negl.	111.122	111.675	0.004	Negl.	111.675	114.241	1.000	Negl.	114.241	114.986	1.000	Negl.	114.986	114.669
Uridine	0.382	Negl.	3.906	4.001	0.009	Negl.	4.001	4.147	1.000	Negl.	4.147	4.218	1.000	Negl.	4.218	4.271
3-Methyl-2-Oxobutyric.Acid	1.000	Negl.	8.674	8.733	0.012	Negl.	8.733	8.412	1.000	Negl.	8.412	8.490	1.000	Negl.	8.490	8.513
2-Hydroxybutyrate	1.000	Negl.	34.927	35.782	1.000	Negl.	35.782	35.451	0.000	Negl.	35.451	37.225	0.138	Negl.	37.225	38.309
Asparagine	1.000	Negl.	58.770	58.900	1.000	Negl.	58.900	57.587	0.045	Negl.	57.587	56.282	1.000	Negl.	56.282	56.169
Threonine	0.119	Negl.	178.109	184.338	1.000	Negl.	184.338	180.601	0.043	Negl.	180.601	175.194	1.000	Negl.	175.194	175.365
3-Hydroxyisobutyrate	1.000	Negl.	10.122	10.380	1.000	Negl.	10.380	10.244	0.006	Negl.	10.244	10.566	0.009	Negl.	10.566	10.895
Formate	1.000	Negl.	10.748	10.611	1.000	Negl.	10.611	10.715	1.000	Negl.	10.715	10.942	0.001	Negl.	10.942	11.287
Phenylalanine	1.000	Negl.	56.086	56.853	1.000	Negl.	56.853	57.285	1.000	Negl.	57.285	57.930	0.001	Negl.	57.930	59.115
Creatinine	1.000	Negl.	51.736	52.637	1.000	Negl.	52.637	53.142	1.000	Negl.	53.142	53.210	1.000	Negl.	53.210	53.318

Supplementary Table S3-2 : female
(3/3)

AgeGroup	60-64 : 65-69		60 - 64	65 - 69	65-69 : 70-74		65 - 69	70 - 74	70-74 : 75-79		70 - 74	75 - 79	75-79 : over80		75 - 79	over 80
Name	p.value	Cliff's delta	Mean	Mean	p.value	Cliff's delta	Mean	Mean	p.value	Cliff's delta	Mean	Mean	p.value	Cliff's delta	Mean	Mean
2-Oxoisocaproate	1.000	Negl.	24.303	24.502	0.036	Negl.	24.502	24.001	0.003	small	24.001	25.188	1.000	Negl.	25.188	24.583
3-Methyl-2-Oxovalerate	1.000	Negl.	18.273	18.423	1.000	Negl.	18.423	18.318	0.003	small	18.318	19.168	1.000	Negl.	19.168	18.946
Tryptophan	1.000	Negl.	49.588	49.198	1.000	Negl.	49.198	49.029	1.000	Negl.	49.029	48.930	1.000	Negl.	48.930	47.780
Leucine	0.819	Negl.	105.795	107.095	1.000	Negl.	107.095	106.668	1.000	Negl.	106.668	108.568	1.000	Negl.	108.568	105.908
2-Aminobutyrate	0.014	Negl.	16.296	16.574	1.000	Negl.	16.574	16.643	1.000	Negl.	16.643	16.986	1.000	Negl.	16.986	16.805
Valine	0.012	Negl.	194.429	197.888	1.000	Negl.	197.888	198.782	1.000	Negl.	198.782	203.862	1.000	Negl.	203.862	203.949
Isoleucine	0.032	Negl.	54.492	55.707	1.000	Negl.	55.707	56.459	0.066	Negl.	56.459	59.102	1.000	Negl.	59.102	60.011
Acetone	1.000	Negl.	6.709	6.951	0.667	Negl.	6.951	6.581	0.000	small	6.581	8.640	1.000	Negl.	8.640	8.376
Lactate	1.000	Negl.	2101.552	2100.079	0.252	Negl.	2100.079	2141.317	0.001	small	2141.317	1999.748	1.000	small	1999.748	1835.904
Pyruvate	1.000	Negl.	82.880	83.610	0.003	Negl.	83.610	86.664	0.000	medium	86.664	66.189	1.000	small	66.189	58.941
Glycerol	0.001	Negl.	77.464	81.489	1.000	Negl.	81.489	83.104	0.000	small	83.104	95.123	1.000	Negl.	95.123	103.283
Glucose	1.000	Negl.	5139.504	5194.870	0.909	Negl.	5194.870	5276.945	1.000	Negl.	5276.945	5356.952	1.000	Negl.	5356.952	5360.525
Cysteine	0.001	Negl.	58.696	59.961	0.000	Negl.	59.961	61.891	1.000	Negl.	61.891	63.385	1.000	Negl.	63.385	63.906
Glutamine	1.000	Negl.	484.783	486.327	0.032	Negl.	486.327	492.110	1.000	Negl.	492.110	498.023	1.000	Negl.	498.023	502.171
Ornithine	1.000	Negl.	68.382	68.867	0.000	Negl.	68.867	70.975	0.000	small	70.975	66.846	1.000	Negl.	66.846	66.296
Lysine	1.000	Negl.	129.853	130.102	0.412	Negl.	130.102	131.605	1.000	Negl.	131.605	129.615	1.000	Negl.	129.615	126.329
Tyrosine	0.093	Negl.	65.428	66.548	0.000	Negl.	66.548	68.888	1.000	Negl.	68.888	68.485	1.000	Negl.	68.485	69.275
Glutamate	1.000	Negl.	48.412	48.349	0.048	Negl.	48.349	49.617	0.001	small	49.617	45.934	1.000	Negl.	45.934	47.789
Citrate	0.005	Negl.	113.634	115.523	1.000	Negl.	115.523	116.494	0.613	Negl.	116.494	120.617	1.000	Negl.	120.617	121.859
Betaine	1.000	Negl.	47.409	47.859	1.000	Negl.	47.859	47.846	1.000	Negl.	47.846	49.395	1.000	Negl.	49.395	49.693
Histidine	1.000	Negl.	82.728	82.571	1.000	Negl.	82.571	81.905	1.000	Negl.	81.905	82.458	1.000	Negl.	82.458	81.656
Carnitine	1.000	Negl.	37.740	37.694	0.024	Negl.	37.694	36.916	0.000	small	36.916	39.358	1.000	Negl.	39.358	40.046
Succinate	1.000	Negl.	7.855	7.960	0.000	Negl.	7.960	8.338	0.000	small	8.338	7.738	1.000	Negl.	7.738	7.413
Acetate	0.030	Negl.	36.187	37.230	1.000	Negl.	37.230	37.009	0.000	small	37.009	40.915	1.000	small	40.915	47.021
Arginine	1.000	Negl.	48.845	49.095	0.133	Negl.	49.095	49.940	0.016	Negl.	49.940	52.849	1.000	Negl.	52.849	52.418
Creatine	1.000	Negl.	42.272	42.514	1.000	Negl.	42.514	42.733	1.000	Negl.	42.733	43.546	1.000	Negl.	43.546	41.560
Methionine	1.000	Negl.	26.024	26.047	1.000	Negl.	26.047	26.149	0.064	Negl.	26.149	25.460	1.000	Negl.	25.460	25.238
Serine	1.000	Negl.	114.669	114.695	1.000	Negl.	114.695	114.512	0.326	Negl.	114.512	117.341	1.000	Negl.	117.341	119.866
Uridine	1.000	Negl.	4.271	4.292	1.000	Negl.	4.292	4.234	1.000	Negl.	4.234	4.378	1.000	Negl.	4.378	4.269
3-Methyl-2-Oxobutyric.Acid	1.000	Negl.	8.513	8.611	1.000	Negl.	8.611	8.530	1.000	Negl.	8.530	8.621	1.000	Negl.	8.621	8.578
2-Hydroxybutyrate	0.288	Negl.	38.309	39.156	1.000	Negl.	39.156	38.463	0.211	Negl.	38.463	41.134	1.000	Negl.	41.134	37.638
Asparagine	1.000	Negl.	56.169	56.045	1.000	Negl.	56.045	56.784	1.000	Negl.	56.784	56.357	1.000	Negl.	56.357	56.222
Threonine	1.000	Negl.	175.365	174.237	0.003	Negl.	174.237	170.104	1.000	Negl.	170.104	173.155	1.000	Negl.	173.155	173.248
3-Hydroxyisobutyrate	0.000	Negl.	10.895	11.268	1.000	Negl.	11.268	11.475	1.000	Negl.	11.475	11.766	1.000	Negl.	11.766	11.307
Formate	0.000	Negl.	11.287	11.737	0.135	Negl.	11.737	12.120	0.282	Negl.	12.120	12.975	1.000	Negl.	12.975	13.908
Phenylalanine	0.000	Negl.	59.115	60.277	0.000	Negl.	60.277	61.984	1.000	Negl.	61.984	63.216	1.000	Negl.	63.216	64.822
Creatinine	1.000	Negl.	53.318	53.684	0.013	Negl.	53.684	54.829	0.000	small	54.829	58.579	1.000	Negl.	58.579	61.136

Supplementary Table S3 : Supplementary Table S3-1 shows the results of age group comparisons at DateDiff0 for males and Supplementary Table S3-2 for females. The p-values and effect size interpretations of the Wilcoxon tests performed between age groups for each metabolite are displayed, together with the mean values for the age groups. ('Negl.' in the table stands for negligible). Between age groups where significant differences have been identified, cells are highlighted in orange if the younger age group has a larger mean value.
Table column names '20-24:25-29, 25-29:30-34, 30-34:35-39, 35-39:40-44, 40-44:45-49, 45-49:50-54, 50-54:55-59, 55-59:60-64, 60-64:65-69, 65-69:70-74, 70-74. The '75-79, 75-79:80over' indicates the age group compared. For example, [30-34:35-39] represents a comparison between the 30-34 age group and the 35-39 age group. If this value is less than 0.05, it indicates that there is a significant difference between the groups and is displayed in red.
The effect size is based on the calculated effect size, with letters coloured green if the interpretation criterion is small size or larger, and if the confidence interval does not cross zero, the cell is coloured green as there is a difference between the two groups compared.

Supplementary Table S4-1 : male
(1/2)

Betaine	VIF	β	StdErr	p.value		Simple Slope Analysis	simple slope	standard error	p.value	
(Intercept)	-	52.959	0.175	0.000	**					
Age	1.522	0.187	0.014	0.000	**					
BMIGroup (High)	1.009	-2.563	0.303	0.000	**					
Age × BMIGroupHigh	1.525	0.066	0.023	0.004	**	Low BMIGroupHigh (-1 SD)	0.178	0.016	0.000	**
						High BMIGroupHigh (+1 SD)	0.24	0.016	0.000	**

Creatinine	VIF	β	StdErr	p.value		Simple Slope Analysis	simple slope	standard error	p.value	
(Intercept)	-	69.109	0.177	0.000	**					
Age	1.522	0.133	0.014	0.000	**					
BMIGroup (High)	1.008	1.355	0.307	0.000	**					
Age × BMIGroupHigh	1.525	0.064	0.023	0.006	**	Low BMIGroupHigh (-1 SD)	0.124	0.016	0.000	**
						High BMIGroupHigh (+1 SD)	0.185	0.016	0.000	**

Cysteine	VIF	β	StdErr	p.value		Simple Slope Analysis	simple slope	standard error	p.value	
(Intercept)	-	63.429	0.197	0.000	**					
Age	1.522	0.367	0.015	0.000	**					
BMIGroup (High)	1.009	4.494	0.342	0.000	**					
Age × BMIGroupHigh	1.525	-0.076	0.026	0.004	**	Low BMIGroupHigh (-1 SD)	0.377	0.018	0.000	**
						High BMIGroupHigh (+1 SD)	0.305	0.017	0.000	**

Glycerol	VIF	β	StdErr	p.value		Simple Slope Analysis	simple slope	standard error	p.value	
(Intercept)	-	63.299	0.352	0.000	**					
Age	1.522	0.424	0.027	0.000	**					
BMIGroup (High)	1.009	3.987	0.61	0.000	**					
Age × BMIGroupHigh	1.525	-0.151	0.047	0.001	**	Low BMIGroupHigh (-1 SD)	0.445	0.031	0.000	**
						High BMIGroupHigh (+1 SD)	0.302	0.031	0.000	**

Serine	VIF	β	StdErr	p.value		Simple Slope Analysis	simple slope	standard error	p.value	
(Intercept)	-	112.196	0.193	0.000	**					
Age	1.522	0.116	0.015	0.000	**					
BMIGroup (High)	1.008	-0.767	0.334	0.022	*					
Age × BMIGroupHigh	1.525	0.052	0.026	0.043	*	Low BMIGroupHigh (-1 SD)	0.108	0.017	0.000	**
						High BMIGroupHigh (+1 SD)	0.157	0.017	0.000	**

Tryptophan	VIF	β	StdErr	p.value		Simple Slope Analysis	simple slope	standard error	p.value	
(Intercept)	-	55.905	0.124	0.000	**					
Age	1.522	-0.086	0.01	0.000	**					
BMIGroup (High)	1.009	0.795	0.215	0.000	**					
Age × BMIGroupHigh	1.525	0.04	0.016	0.015	*	Low BMIGroupHigh (-1 SD)	-0.091	0.011	0.000	**
						High BMIGroupHigh (+1 SD)	-0.054	0.011	0.000	**

Tyrosine	VIF	β	StdErr	p.value		Simple Slope Analysis	simple slope	standard error	p.value	
(Intercept)	-	71.075	0.18	0.000	**					
Age	1.522	0.221	0.014	0.000	**					
BMIGroup (High)	1.009	5.568	0.311	0.000	**					
Age × BMIGroupHigh	1.525	-0.056	0.024	0.019	*	Low BMIGroupHigh (-1 SD)	0.229	0.016	0.000	**
						High BMIGroupHigh (+1 SD)	0.176	0.016	0.000	**

2-Oxoisocaproate	VIF	β	StdErr	p.value		Simple Slope Analysis	simple slope	standard error	p.value	
(Intercept)	-	30.671	0.085	0.000	**					
Age	1.522	-0.052	0.007	0.000	**					
BMIGroup (High)	1.009	1.562	0.147	0.000	**					
Age × BMIGroupHigh	1.525	-0.023	0.011	0.039	*	Low BMIGroupHigh (-1 SD)	-0.049	0.007	0.000	**
						High BMIGroupHigh (+1 SD)	-0.071	0.007	0.000	**

3-Hydroxybutyrate	VIF	β	StdErr	p.value		Simple Slope Analysis	simple slope	standard error	p.value	
(Intercept)	NA	78.936	1.004	0.000	**					
Age	1.522	0.877	0.078	0.000	**					
BMIGroup (High)	1.009	-11.213	1.738	0.000	**					
Age × BMIGroupHigh	1.525	-0.435	0.133	0.001	**	Low BMIGroupHigh (-1 SD)	0.937	0.089	0.000	**
						High BMIGroupHigh (+1 SD)	0.526	0.089	0.000	**

Supplementary Table S4-1 : male
(2/2)

3-Hydroxyisobutyrate	VIF	β	StdErr	p.value		Simple Slope Analysis	simple slope	standard error	p.value	
(Intercept)	-	12.634	0.049	0.000	**					
Age	1.522	0.035	0.004	0.000	**					
BMIGroup (High)	1.009	1.215	0.085	0.000	**					
Age × BMIGroupHigh	1.525	-0.027	0.006	0.000	**	Low BMIGroupHigh (-1 SD)	0.039	0.004	0.000	**
						High BMIGroupHigh (+1 SD)	0.013	0.004	0.002	**

3-Methyl-2-Oxobutyric.Acid	VIF	β	StdErr	p.value		Simple Slope Analysis	simple slope	standard error	p.value	
(Intercept)	-	9.7	0.027	0.000	**					
Age	1.522	-0.01	0.002	0.000	**					
BMIGroup (High)	1.009	0.622	0.047	0.000	**					
Age × BMIGroupHigh	1.525	-0.009	0.004	0.016	*	Low BMIGroupHigh (-1 SD)	-0.009	0.002	0.000	**
						High BMIGroupHigh (+1 SD)	-0.017	0.002	0.000	**

Asparagine	VIF	β	StdErr	p.value		Simple Slope Analysis	simple slope	standard error	p.value	
(Intercept)	-	59.816	0.153	0.000	**					
Age	1.522	0.024	0.012	0.042	*					
BMIGroup (High)	1.009	-2.133	0.265	0.000	**					
Age × BMIGroupHigh	1.525	0.088	0.02	0.000	**	Low BMIGroupHigh (-1 SD)	0.012	0.014	0.378	
						High BMIGroupHigh (+1 SD)	0.095	0.014	0.000	**

Lactate	VIF	β	StdErr	p.value		Simple Slope Analysis	simple slope	standard error	p.value	
(Intercept)	-	2522.049	8.388	0.000	**					
Age	1.522	-1.67	0.648	0.010	*					
BMIGroup (High)	1.009	148.668	14.529	0.000	**					
Age × BMIGroupHigh	1.525	-3.201	1.112	0.004	**	Low BMIGroupHigh (-1 SD)	-1.231	0.747	0.099	
						High BMIGroupHigh (+1 SD)	-4.253	0.74	0.000	**

Glycine	VIF	β	StdErr	p.value		Simple Slope Analysis	simple slope	standard error	p.value	
(Intercept)	-	196.135	0.483	0.000	**					
Age	1.522	-0.211	0.037	0.000	**					
BMIGroup (High)	1.009	-11.525	0.836	0.000	**					
Age × BMIGroupHigh	1.525	0.28	0.064	0.000	**	Low BMIGroupHigh (-1 SD)	-0.25	0.043	0.000	**
						High BMIGroupHigh (+1 SD)	0.015	0.043	0.734	

Proline	VIF	β	StdErr	p.value		Simple Slope Analysis	simple slope	standard error	p.value	
(Intercept)	-	177.985	0.532	0.000	**					
Age	1.522	-0.152	0.041	0.000	**					
BMIGroup (High)	1.009	8.005	0.921	0.000	**					
Age × BMIGroupHigh	1.525	0.181	0.071	0.010	*	Low BMIGroupHigh (-1 SD)	-0.177	0.047	0.000	**
						High BMIGroupHigh (+1 SD)	-0.007	0.047	0.888	

Threonine	VIF	β	StdErr	p.value		Simple Slope Analysis	simple slope	standard error	p.value	
(Intercept)	-	186.071	0.546	0.000	**					
Age	1.522	-0.141	0.042	0.001	**					
BMIGroup (High)	1.009	-3.068	0.946	0.001	**					
Age × BMIGroupHigh	1.525	0.21	0.072	0.004	**	Low BMIGroupHigh (-1 SD)	-0.17	0.049	0.000	**
						High BMIGroupHigh (+1 SD)	0.029	0.048	0.551	

2-Hydroxybutyrate	VIF	β	StdErr	p.value		Simple Slope Analysis	simple slope	standard error	p.value	
(Intercept)	-	38.566	0.187	0.000	**					
Age	1.522	0.103	0.014	0.000	**					
BMIGroup (High)	1.009	2.47	0.323	0.000	**					
Age × BMIGroupHigh	1.525	-0.088	0.025	0.000	**	Low BMIGroupHigh (-1 SD)	0.115	0.017	0.000	**
						High BMIGroupHigh (+1 SD)	0.032	0.016	0.050	

Supplementary Table S4-2 : female

Citrate	VIF	β	StdErr	p.value		Simple Slope Analysis	simple slope	standard error	p.value	
(Intercept)	-	111.456	0.228	0.000	**					
Age	1.458	0.409	0.018	0.000	**					
BMIGroup (High)	1.002	-3.656	0.394	0.000	**					
Age × BMIGroupHigh	1.458	0.138	0.032	0.000	**	Low BMIGroupHigh (-1 SD)	0.39	0.021	0.000	**
						High BMIGroupHigh (+1 SD)	0.521	0.022	0.000	**

Cysteine	VIF	β	StdErr	p.value		Simple Slope Analysis	simple slope	standard error	p.value	
(Intercept)	-	55.764	0.134	0.000	**					
Age	1.458	0.397	0.011	0.000	**					
BMIGroup (High)	1.002	2.815	0.233	0.000	**					
Age × BMIGroupHigh	1.458	0.04	0.019	0.035	*	Low BMIGroupHigh (-1 SD)	0.391	0.012	0.000	**
						High BMIGroupHigh (+1 SD)	0.429	0.013	0.000	**

Glutamate	VIF	β	StdErr	p.value		Simple Slope Analysis	simple slope	standard error	p.value	
(Intercept)	-	46.191	0.145	0.000	**					
Age	1.458	0.195	0.011	0.000	**					
BMIGroup (High)	1.002	7.974	0.251	0.000	**					
Age × BMIGroupHigh	1.458	-0.088	0.02	0.000	**	Low BMIGroupHigh (-1 SD)	0.207	0.013	0.000	**
						High BMIGroupHigh (+1 SD)	0.124	0.014	0.000	**

Leucine	VIF	β	StdErr	p.value		Simple Slope Analysis	simple slope	standard error	p.value	
(Intercept)	-	104.086	0.218	0.000	**					
Age	1.458	0.156	0.017	0.000	**					
BMIGroup (High)	1.002	7.502	0.378	0.000	**					
Age × BMIGroupHigh	1.458	-0.086	0.031	0.005	**	Low BMIGroupHigh (-1 SD)	0.167	0.02	0.000	**
						High BMIGroupHigh (+1 SD)	0.087	0.021	0.000	**

Serine	VIF	β	StdErr	p.value		Simple Slope Analysis	simple slope	standard error	p.value	
(Intercept)	-	114.219	0.176	0.000	**					
Age	1.457	0.073	0.014	0.000	**					
BMIGroup (High)	1.002	-2.189	0.304	0.000	**					
Age × BMIGroupHigh	1.458	0.118	0.025	0.000	**	Low BMIGroupHigh (-1 SD)	0.057	0.016	0.000	**
						High BMIGroupHigh (+1 SD)	0.168	0.017	0.000	**

Valine	VIF	β	StdErr	p.value		Simple Slope Analysis	simple slope	standard error	p.value	
(Intercept)	-	191.302	0.399	0.000	**					
Age	1.458	0.411	0.031	0.000	**					
BMIGroup (High)	1.002	15.606	0.691	0.000	**					
Age × BMIGroupHigh	1.458	-0.187	0.056	0.001	**	Low BMIGroupHigh (-1 SD)	0.437	0.036	0.000	**
						High BMIGroupHigh (+1 SD)	0.261	0.038	0.000	**

2-Aminobutyrate	VIF	β	StdErr	p.value		Simple Slope Analysis	simple slope	standard error	p.value	
(Intercept)	-	16.011	0.033	0.000	**					
Age	1.458	0.035	0.003	0.000	**					
BMIGroup (High)	1.002	1.276	0.057	0.000	**					
Age × BMIGroupHigh	1.458	-0.014	0.005	0.002	**	Low BMIGroupHigh (-1 SD)	0.037	0.003	0.000	**
						High BMIGroupHigh (+1 SD)	0.024	0.003	0.000	**

Lactate	VIF	β	StdErr	p.value		Simple Slope Analysis	simple slope	standard error	p.value	
(Intercept)	-	2093.556	6.216	0.000	**					
Age	1.458	-1.338	0.489	0.006	**					
BMIGroup (High)	1.002	136.637	10.768	0.000	**					
Age × BMIGroupHigh	1.458	-2.845	0.873	0.001	**	Low BMIGroupHigh (-1 SD)	-0.946	0.565	0.094	
						High BMIGroupHigh (+1 SD)	-3.629	0.59	0.000	**

Asparagine	VIF	β	StdErr	p.value		Simple Slope Analysis	simple slope	standard error	p.value	
(Intercept)	-	56.985	0.135	0.000	**					
Age	1.458	-0.063	0.011	0.000	**					
BMIGroup (High)	1.002	-2.000	0.234	0.000	**					
Age × BMIGroupHigh	1.458	0.071	0.019	0.000	**	Low BMIGroupHigh (-1 SD)	-0.072	0.012	0.000	**
						High BMIGroupHigh (+1 SD)	-0.005	0.013	0.668	

Supplementary Table S4 : Supplementary Table S4-1 presents the results of multiple regression analysis by metabolite for males and Supplementary Table S4-2 for females. The results of the analysis using BMI and age as independent variables and metabolite concentration as dependent variable, and only metabolites for which significant main effects and interactions were found are listed. The rightmost column of the table for each metabolite represents the results of a simple gradient analysis in the high BMI group; VIF : Variance Inflation Factor, b : regression coefficient, * in the table indicates the significance level (**: $p<0.01$, *: $p<0.05$).

Supplementary Table S5-1 : male

Citrate	VIF	β	StdErr	p.value	
(Intercept)	-	100.186	0.354	0.000	**
Age	3.462	0.521	0.025	0.000	**
postprandial time (Any Time)	1.001	-3.73	0.417	0.000	**
Age × Any Time	3.46	-0.085	0.03	0.004	**

Formate	VIF	β	StdErr	p.value	
(Intercept)	-	10.812	0.067	0.000	**
Age	3.449	0.035	0.005	0.000	**
postprandial time (Any Time)	1.001	1.069	0.079	0.000	**
Age × Any Time	3.447	0.036	0.006	0.000	**

Methionine	VIF	β	StdErr	p.value	
(Intercept)	-	28.484	0.069	0.000	**
Age	3.462	-0.013	0.005	0.008	**
postprandial time (Any Time)	1.001	0.633	0.081	0.000	**
Age × Any Time	3.46	-0.013	0.006	0.020	*

Pyruvate	VIF	β	StdErr	p.value	
(Intercept)	-	90.286	0.514	0.000	**
Age	3.462	-0.082	0.037	0.026	*
postprandial time (Any Time)	1.001	1.576	0.604	0.009	*
Age × Any Time	3.46	0.179	0.043	0.000	**

2-Oxoisocaproate	VIF	β	StdErr	p.value	
(Intercept)	-	31.897	0.116	0.000	**
Age	3.462	-0.063	0.008	0.000	**
postprandial time (Any Time)	1.001	-1.944	0.137	0.000	**
Age × Any Time	3.46	0.038	0.01	0.000	**

3-Methyl-2-Oxobutyric.Acid	VIF	β	StdErr	p.value	
(Intercept)	-	10.033	0.038	0.000	**
Age	3.462	-0.015	0.003	0.000	**
postprandial time (Any Time)	1.001	-0.488	0.044	0.000	**
Age × Any Time	3.46	0.015	0.003	0.000	**

3-Methyl-2-Oxovalerate	VIF	β	StdErr	p.value	
(Intercept)	-	22.946	0.082	0.000	**
Age	3.462	-0.045	0.006	0.000	**
postprandial time (Any Time)	1.001	-0.47	0.097	0.000	**
Age × Any Time	3.46	0.028	0.007	0.000	**

Supplementary Table S5-2 : female

Acetate	VIF	β	StdErr	p.value	
(Intercept)	-	33.63	0.155	0.000	**
Age	2.896	0.112	0.012	0.000	**
postprandial time (Any Time)	1.006	2.061	0.194	0.000	**
Age × Any Time	2.886	0.057	0.015	0.000	**

Betaine	VIF	β	StdErr	p.value	
(Intercept)	-	43.554	0.166	0.000	**
Age	2.896	0.175	0.013	0.000	**
postprandial time (Any Time)	1.006	2.845	0.206	0.000	**
Age × Any Time	2.886	0.047	0.016	0.003	**

Formate	VIF	β	StdErr	p.value	
(Intercept)	-	10.543	0.047	0.000	**
Age	2.878	0.011	0.004	0.002	**
postprandial time (Any Time)	1.006	1.189	0.059	0.000	**
Age × Any Time	2.867	0.034	0.004	0.000	**

Glutamine	VIF	β	StdErr	p.value	
(Intercept)	-	470.966	0.86	0.000	**
Age	2.896	1.285	0.066	0.000	**
postprandial time (Any Time)	1.006	3.58	1.071	0.001	**
Age × Any Time	2.886	0.25	0.082	0.002	**

Leucine	VIF	β	StdErr	p.value	
(Intercept)	-	101.053	0.276	0.000	**
Age	2.896	0.092	0.021	0.000	**
postprandial time (Any Time)	1.006	5.586	0.344	0.000	**
Age × Any Time	2.886	0.088	0.026	0.001	**

Ornithine	VIF	β	StdErr	p.value	
(Intercept)	-	65.778	0.195	0.000	**
Age	2.896	0.165	0.015	0.000	**
postprandial time (Any Time)	1.006	3.281	0.242	0.000	**
Age × Any Time	2.886	0.063	0.018	0.001	**

Phenylalanine	VIF	β	StdErr	p.value	
(Intercept)	-	55.922	0.14	0.000	**
Age	2.896	0.115	0.011	0.000	**
postprandial time (Any Time)	1.006	4.443	0.174	0.000	**
Age × Any Time	2.886	0.048	0.013	0.000	**

Succinate	VIF	β	StdErr	p.value	
(Intercept)	-	8.164	0.024	0.000	**
Age	2.896	0.017	0.002	0.000	**
postprandial time (Any Time)	1.006	-0.397	0.03	0.000	**
Age × Any Time	2.886	0.005	0.002	0.039	*

Tyrosine	VIF	β	StdErr	p.value	
(Intercept)	-	60.578	0.194	0.000	**
Age	2.896	0.157	0.015	0.000	**
postprandial time (Any Time)	1.006	6.32	0.241	0.000	**
Age × Any Time	2.886	0.084	0.018	0.000	**

Valine	VIF	β	StdErr	p.value	
(Intercept)	-	182.233	0.503	0.000	**
Age	2.896	0.298	0.039	0.000	**
postprandial time (Any Time)	1.006	15.448	0.627	0.000	**
Age × Any Time	2.886	0.119	0.048	0.013	*

2-Aminobutyrate	VIF	β	StdErr	p.value	
(Intercept)	-	15.393	0.041	0.000	**
Age	2.896	0.025	0.003	0.000	**
postprandial time (Any Time)	1.006	1.063	0.051	0.000	**
Age × Any Time	2.886	0.015	0.004	0.000	**

3-Hydroxyisobutyrate	VIF	β	StdErr	p.value	
(Intercept)	-	10.194	0.044	0.000	**
Age	2.896	0.024	0.003	0.000	**
postprandial time (Any Time)	1.006	0.989	0.055	0.000	**
Age × Any Time	2.886	0.013	0.004	0.002	**

3-Methyl-2-Oxovalerate	VIF	β	StdErr	p.value	
(Intercept)	-	17.627	0.053	0.000	**
Age	2.896	-0.039	0.004	0.000	**
postprandial time (Any Time)	1.006	1.152	0.065	0.000	**
Age × Any Time	2.886	0.032	0.005	0.000	**

3-Methyl-2-Oxobutyric.Acid	VIF	β	StdErr	p.value	
(Intercept)	-	8.641	0.026	0.000	**
Age	2.896	-0.014	0.002	0.000	**
postprandial time (Any Time)	1.006	-0.078	0.032	0.014	*
Age × Any Time	2.886	0.012	0.002	0.000	**

Creatine	VIF	β	StdErr	p.value	
(Intercept)	-	39.334	0.184	0.000	**
Age	2.896	0.201	0.014	0.000	**
postprandial time (Any Time)	1.006	2.189	0.229	0.000	**
Age × Any Time	2.886	-0.042	0.017	0.015	*

Supplementary Table S5 : Supplementary Table S5-1 presents the results of multiple regression analyses by metabolite for males and Supplementary Table S5-1 for females. Only metabolites for which significant main effects and interactions were found are listed. b : regression coefficient, VIF : Variance Inflation Factor, * in the table indicates the significance level (**: p<0.01, *: p<0.01), significance level (**: p<0.01, *: p<0.05).

Supplementary Table S6

Types of cohort studies	ToMMo : Tohoku University Tohoku Medical MegabankOrganization	jMorp2022 Baseline (Non pregnant)
Community-Based Cohort Study	Number	Number
Specific health check-up joint participation type	40,260	22,420
Community Support Center type	13,744	13,373
Birth and Three-Generation Cohort Study	Number	Number
pregnant women (family role: mother)	43,677	0
women's partners (family role: father)	7,228	2,194
their parents (family role: grandparents)	7,537	2,540
their children (Sibling of newborn: Adults 20 years and over)	2,317	2
total	114,763	40,529

Supplementary Table S6 : The number of participants in a community-based cohort study, birth and three-generation cohort study, and the number of participants of the baseline study, excluding pregnant female.

Supplementary Table S7

Supplementary Table S7-1 : male

DateDiff0 (DateDiff1)	Group range	N	Min.	median	Max.
Low	< 21.844 (< 21.841)	2,986 (843)	14.62 (14.69)	20.58(20.56)	21.84 (21.84)
Middle	21.844 - 25.834 (21.841-25.698)	5,929 (1,681)	21.85 (21.84)	23.71 (23.56)	25.83 (25.70)
High	25.834 < (25.698 <)	2,989 (841)	25.84 (25.70)	27.53 (27.39)	50.44 (51.59)

Supplementary Table S7-2 : female

DateDiff0 (DateDiff1)	Group range	N	Min.	median	Max.
Low	< 20.047 (< 19.989)	4,056 (1,585)	11.96 (13.76)	18.84 (18.83)	20.05 (19.99)
Middle	20.047 - 24.581 (19.989 - 24.426)	8,079 (3,167)	20.05 (19.99)	22.07 (21.91)	24.58 (24.42)
High	24.581 < (24.426 <)	4,044 (1,584)	24.58 (24.43)	26.64 (26.54)	52.78 (42.10)

Supplementary Table S7 : Supplementary Table S7-1 presents summary information on BMI in males and Supplementary Table S7-2 in females; BMI is divided into three groups based on quartiles, with row 1 for the low group, row 2 for the intermediate group and row 3 for the high group; range and sample size, statistical indices are shown. Information on DateDiff1 is given in parentheses ( ).
